# Comparing Walker's (2008) skull trait sex estimation standard to proteomic sex estimation for a group of South Asian individuals

**DOI:** 10.1016/j.fsisyn.2023.100450

**Published:** 2024-01-02

**Authors:** Laura M. Rogers, Siân E. Halcrow, Torsten Kleffmann, Charlotte L. King

**Affiliations:** a Department of Anatomy, School of Biomedical Sciences, University of Otago, Dunedin, New Zealand; b Department of Biochemistry, School of Biomedical Sciences, University of Otago, Dunedin, New Zealand

**Keywords:** Forensic anthropology, Sexual dimorphism, Sex estimation, Population variation

## Abstract

This research assesses the potential for misidentification of sex in individuals of South Asian ancestry using the Walker (2008) morphological skull sex estimation standard [1]. Chromosomal sex was assessed using proteomic analysis targeting sex chromosome-specific amylogenic peptides. Results showed that the Walker method produced incorrect classification for 36.7 % of individuals. Overwhelmingly, those incorrectly assigned were chromosomally male. Misidentification was due to males within the group having lower trait scores (i.e., more gracile traits) than the standard would predict. There was also a high level of overlap in trait scores between male and females indicating reduced expression of sexual dimorphism. The use of established multivariate statistical techniques improved accuracy of sex estimation in some cases, but larger osteological data sets from South Asian individuals are required to develop population-specific standards. We suggest that peptide analysis may provide a useful tool for the forensic anthropologist when assessing sex in populations without population specific osteological standards.

## 1 Introduction/background

Chromosomal sex is a key factor in the reconstruction of identity from skeletal remains in forensic and bioarcheological contexts [[1], [2], [3], [4], [5], [6], [7], [8], [9]]. Osteological estimation of sex commonly falls into two categories: non-metric; and metric sex estimation. Of these, non-metric sex estimation is most readily used as it is easy to apply [10,11], while metric sex estimation requires specialized anthropometric equipment restricting its use to the availability of such tools [10]. Features of the pelvis are considered the most accurate non-metric indicators [1,12,13], however pelvic features are not always present or well preserved enough to aid in sex estimation. In such cases, certain sexually dimorphic non-metric features of the skull are therefore used for sex estimation [14]. Metric analysis of some post-cranial bones has shown improved accuracy in comparison with non-metric analysis of the skull in American Black and White populations [12].

In general, non-metric techniques are based on the scored expression of sexually dimorphic traits. The standards for scoring these traits have been created using observations of relatively large skeletal collections of known sex [15]. This allows the researcher to understand what gracile expression (typically associated with ‘female’ morphology) of a trait looks like relative to the robust expression (typically associated with ‘male’ morphology). Individuals of unknown sex can then be compared to these graded scales to document whether the morphology of the remains is stereotypically male or stereotypically female [10,15,16]. However, many of the non-metric standards currently in use are based on collections derived from American or European populations and do not encompass the range of morphological expression seen globally [17]. It is likely that these generalized standards are used more widely than may be appropriate, and numerous studies have now begun to quantify the potential inaccuracies that the application of these methods cause [10,[18], [19], [20], [21], [22], [23], [24], [25], [26], [27]]. We know that there is significant morphological variation among population groups due to differences in the genetic, environmental, and social influences acting upon them [[28], [29], [30], [31]]. This population-based variation in morphology can also correlate with how sexual dimorphism manifests in the skeleton as sexually dimorphic skeletal features are actively affected by both the internal and external influences listed above [10,13,17,19,28,[32], [33], [34]]. Perception of sexually dimorphic features are also affected by an observer's preconceived ideas of what is male-like versus female-like; concepts that are often tied to social-cultural expectations [35,36]. It is therefore important that sex estimation techniques consider population-specific factors that may affect non-metric trait expression.

Asian populations have been underrepresented within anatomical research globally, with most morphological studies comprising small sample size or limited ancestral groups, despite Asia's vast size and population variation [37]. More specifically, research into the expression of sexual dimorphism in South Asian groups is lacking, although previous studies of sexual dimorphism in other regions of Asia have shown there are differences in trait expression relative to groups from European regions [13,24,26]. Evaluation of non-metric sex estimation methods in Thai, Japanese, and Filipino populations, for example, has shown individuals have less phenotypic sexual dimorphism compared with U.S. Black and U.S. White groups, suggesting that morphological sex differences in some Asian populations are more nuanced [27,28]. Both male and female individuals from these Asian populations are noted as being more generally gracile suggesting that males may be misidentified as female when analyzed using traditional sex estimation standards [13,26].

Walker's standard [10] for sexually dimorphic trait expression in the skull is one of the most commonly applied skull-based sex estimation standards in forensics and bioarcheology [27,28]. The standard presents five skull features; the nuchal crest, the mastoid process, the supra-orbital margin, the supra-orbital ridge, and the mental eminence. An ordinal scoring scale from 1 to 5 is used indicating the most gracile to most robust expression respectively.

These traits were first identified as sexually dimorphic in 1970 by Acsádi and Nemeskéri and subsequently included in Buikstra and Ubelaker's (1994) popular standards publication [15,38]. Walker (2008) revised this standard, finding that the illustrations produced by Acsádi and Nemeskéri (1970) were not representative of the variation seen globally. New diagrams were produced, and the grading system was converted to the 1–5 scale used today. Acsádi and Nemeskéri (1970) graded morphology on a scale of −2 to +2, however, this scale implied that a score of ‘0’ would inherently always equal the middle cut off between males and females. In actuality, the cut point between male and female scores varies between populations and it is uncommon for the middle value, ‘3’ in the case of this standard to be truly ambiguous. This scoring system was created following observations of skeletal collections comprised of individuals largely of European and African descent [17,25]. Asian individuals were not represented in this initial sample collection, although a small number of Native American individuals were included and erroneously considered by some to be appropriate proxies for Asian skeletal morphology [25,39,40]. A growing number of studies have shown that Walker's sex estimation standard does not adequately represent the sexual dimorphism presented by several populations, resulting in incorrect sex estimation for many individuals [18,24,27,41,42]. In spite of this, these methods remain the go-to for many anthropologists due to the perceived benefits of the method [43].

In this study we assessed the reliability of Walker's scoring method [10] on a collection of individuals with South Asian ancestry held in a teaching museum at the University of Otago. These individuals are part of the Anatomy Museum's collection. they were collected historically and continue to be used for teaching purposes today. The results of analysis of sex chromosome-specific enamel protein isoforms were used to provide us with known chromosomal sex information from which the results of Walker's method [10] could be compared. Chromosomal linked peptide sex estimation has been undertaken to contribute to the development of osteobiographic profiles for individuals within this collection. Osteobiographies are an integral step in gathering pieces of information about who individuals were in life to restore a sense of personhood for the people in these collections [44,45].

The analysis of sex chromosome-specific protein isoforms by mass spectrometry is a relatively new method gaining traction in the field of bioarcheology [[46], [47], [48], [49], [50], [51], [52], [53], [54]]. The technique has been less adopted within forensic anthropology, which has primarily focused on DNA based methods for sex estimation [55]. The protein amelogenin dominates the literature on mass spectrometry-based sex estimation as the only sex-linked protein identified in skeletal remains [56,57]. Amelogenin is expressed on both the X and Y chromosomes, encoding for the proteins AMELX and AMELY respectively [25]. Variations in the amino acid sequences of these isoforms allow for the sex of an individual to be identified based on the presence or absence of peptides from AMELY detected by mass spectrometry (AMELX/Y-MS) [25,27].

Despite being a relatively new technique, peptide analysis has significant potential to change the way that sex estimation is undertaken in forensic and biological anthropology. The technique is both more cost-effective and easier to undertake than other molecular sex estimation techniques (e.g. DNA) [48,55]. In addition, amelogenin is found within dental enamel, where it aids in mineralization. The resistance of dental enamel to post-depositional diagenetic alteration means that amelogenin is much more likely to be preserved within archaeological and forensic contexts than organic molecules in bone tissue [49,52,58]. As such, amelogenin has been detected in the enamel of both contemporary and ancient individuals of varying ages, including teeth from perinatal infants [[47], [48], [49],^59^]. This technique is minimally destructive, requiring a microscopic amount of dental enamel.

### 1.1 A note on the terminological use of sex in this study

Chromosomal sex is a primary characteristic referring to the biological differences between females and males, determined by the presence or absence of a Y chromosome [35,36]. This is not the definition of gender, as although chromosomal sex and gender are often intwined, they are also distinct [60]. Gender is defined as a social construct that can manifest in a variety of ways, which can have varying meanings cross-culturally [7,[61], [62], [63]]. It is important to avoid viewing individuals in a binary light and acknowledge the sociocultural complexity of both sex and gender [64]. Although individual remains may present as morphologically and/or chromosomally one sex, the individual may not necessarily have identified with preconceived ideas or practices associated with that sex. Therefore, a wide range of social implications should be assessed when undertaking anthropological and forensic research rather than a sole reliance on markers of chromosomal sex [55,62,65].

## 2 Materials and methods

This study focuses on 34 skeletal adult individuals of South Asian ancestry housed within the Department of Anatomy at the University of Otago, New Zealand. These individuals were acquired historically, likely through the Kolkata bone trade [66,67]. The history of the Kolkata bone trade and its impacts have been outlined previously [66,68]. The trade resulted in both the colonially sanctioned and later illegal removal of human remains out of India to global buyers [66,[69], [70], [71]]. Collection of human remains in such a manner has not been an uncommon practice as large parts of physical anthropology collections comprise the skeletons of Indigenous people that were removed from British colonies [66,72,73]. Many of these remains were collected to measure and identify physical distinctions of race, with intentions rooted in racist ideology to “Other” non-western groups [[72], [73], [74], [75]]. After the 1832 Anatomy Act was introduced to combat grave robbing in Britain [66,67,69], continued increase in demand for anatomical study skeletons led to the outsourcing of remains from British governed areas such as the Indian subcontinent, establishing the Kolkata bone trade [66]. As a result of the Bengal Famine in 1934-1944 thousands of deaths per week provided bone traders with access to large quantities of remains [66,76]. The system was increasingly commercialized to maximize exports through theft of remains or exploitation of disenfranchised families of the deceased [67,68]. In response to intensive graverobbing and alleged murders to meet demand, several skeleton export bans were implemented, including the 1985 ban of all Indian skeletal remains, which remains in effect today [66,76]. Despite the bans, reports of illegal exportation of human remains have continued [77]. The exportation of allegedly up to 15,000 skeletons and 50,000 skulls per year during the height of the bone trade has produced vast human remain collections in both institutions and private collections worldwide [66].

It is acknowledged that the historical collection of these individuals does not necessarily align with contemporary best practice standards [73,78,79]. This project is part of research to understand these individuals’ stories and presents an important first step in recognizing their humanity and giving them back their voices in the present day.

Sex estimation was undertaken for each individual following Walker's scoring standard [10]. As categorical scoring can be influenced by factors such as observer experience and time pressure, we tested for intra and inter-observer error [80,81]. Sex was estimated twice by LR in sessions approximately one-month apart, and scores were compared to those obtained by SH in a separate recording session. All individuals were estimated to be adult based on spheno-occipital synchondrosis fusion [63,82]. Both age and sex were estimated observing the skull only due to the likely inclusion of more than one individual within the articulated skeletons in this collection. The co-mingling of articulated remains was a known practice during the era in that these bones were traded as complete skeletons had a higher monetary value, encouraging the substitution of bones where necessary [83].

Sex was determined using peptide (amelogenin) analysis following a protocol based on established standards [[47], [48], [49]]. Peptides were extracted from dental enamel via acid etching. Each tooth was firstly washed with 3 % H_2_O_2_ for 30 s to remove any debris from the enamel surface. The H_2_O_2_ was then decanted, and the tooth was rinsed using MilliQ. The enamel surface was then held in 5 % HCl for 2 min, to dissolve a small amount of enamel from the tooth's surface into solution (called an acid etch). This etching process was done twice with the secondary etch being retained for analysis. Each tooth was then rinsed again with MilliQ water to remove any remaining acid from the enamel surface.

Peptides were isolated from the HCl solution using solid phase extraction. A ZipTip was conditioned firstly with 100 % acetonitrile followed by an equilibrium solution (formic acid 0.1 %; acetonitrile 1 %; in MilliQ) through the ZipTip three times each. The etch solution was then drawn through the tip 15 times to allow the peptides within the sample to bind to the resin within the tip. Any exogenous material from the etch solution was cleared by pulling wash solution (formic acid 0.1 %; acetonitrile 1 %; in MilliQ) through the tip three times. The peptides were then eluted from the tip using 60 μL of an elution buffer (formic acid 0.1 %; acetonitrile 60 %; in MilliQ). The eluted samples were vacuum dried in a centrifugal vacuum concentrator (Savant, SpeedVac SC100) operated at standard speed without heating for approximately 90 min.

The samples were later solubilized (formic acid 0.1 %; acetonitrile 5 %; in MilliQ). Samples were agitated to ensure complete solubilization before being centrifuged (10 min; 30,000 g; 20 °C) to remove any remaining debris. A sample volume of 5 μL was loaded for analysis by nano-flow reversed phase liquid chromatography (nRPLC) coupled to Qq-TOF mass spectrometry (5600+ TripleTOF, AB Sciex). Each sample was analyzed by targeted parallel reaction monitoring (PRM), and untargeted data dependent acquisition (DDA). The list of targeted peptides and their *m*/*z* values is given in Table 1. A blank analysis was run between different samples to avoid carry-over of AMELY-specific signals. The targeted PRM sample runs were analyzed using the Skyline software (Macoss Lab Software, v 21.2). The area under the curve of at least two peptide specific fragment ions was extracted and used for detection and quantification of AMELY and AMELX peptides. The presence and intensity of the AMELX peptides SIRPPYPSY and YEVLYTPLK were used for the assessment of sufficient peptide extraction from the enamel. Untargeted DDA runs were searched against the human amino acid sequence database (Swiss-Prot [September 21, 2020] canonical 20,375 protein count) for quality control and the evaluation of untargeted AMELY/X signals. Peptides targeted in the PRM run, their corresponding amino acid sequences, and the sex chromosome(s) they were associated with are given in Table 1.

**Table 1:** Amelogenin peptides targeted by mass spectrometry.

Peptide mass to charge ratio (m/z)	Amino sequence	Amelogenin isoforms	Links to chromosomal sex
483.7393++	S**M**IRPPYS	AMELY	Present only in males
432.2258++	SMIRPPY	AMELY	Present only in males
440.2233++	S**M**IRPPY	AMELY	Present only in males
540.2796++	SIRPPYPSY	AMELX	Present in both males and females
415.2320++	SIRPPYP	AMELX	Present in both males and females
481.7815++	YEVLTPLK	AMELX	Present in both males and females
723.8619++	LPPHPGHPGYI**N**F	AMEL_XY	Present in both males and females
468.7449++	TPLKWYQ	AMEL_XY	Present in both males and females

**Note**: Amino acids underlined are modified: (**M**) oxidation of methionine, (**N**) oxidation of asparagine

### 2.1 Statistical analysis

Accuracy of sex estimation (correct classification overall and by individual trait expression) was established by the comparison to the proteomic sex estimation results. Trait frequencies were generated following Walker's method [10]. These were based on how often a trait score was assigned to each of the sexes (number of individuals of a sex given a certain score for a trait/the total amount of individuals of that sex in the study group). The probability of a score being indicative of either sex was then calculated based on these frequencies (probability score indicating male = % males/(% females + % males), probability score = female = % females/(% females + % males)). Mean trait scores between male and female individuals were compared using a *t*-test.

To assess the accuracy of each skull trait for sex estimation (using proteomic sex estimation as chromosomal sex), their discriminatory power, and whether accuracy could be increased using statistical modeling, we applied univariate and multivariate statistics. Walker's discriminant function equations [10], designed to impose statistical weighting based on population specific data, were also applied to assess whether sex estimation accuracy could be improved by placing more power on certain traits. We recognize that the ideal in this situation would have been to create our own population specific discriminant function equations in order to take into account population-specific variation. However, the small sample size limited our ability to create equations with any statistical power.

All statistical analysis was carried out using SPSS Statistics software (IBM SPSS Statistics V. 28.0.1.0 (142)). Intra- and interobserver error was assessed using the Cohens kappa measure of agreement [84,85].

## 3 Results

### 3.1 Inter/intra observer error

Intra- and interobserver error analysis indicated that this had little effect on the sex estimation outcome. Of the 160 scores compared for intra-observer error, 152 of these were scored within 1 score between the first and second round of marking (95.0 %). 48.1 % were given the same score. 28 individuals were given scores that indicated sex in both rounds of marking. Of these individuals, there was agreement on sex estimation for 26 of these individuals (92.8 %). The Cohens kappa measure of agreement indicated that the nuchal crest, the supra-orbital ridge, and the mental eminence had fair agreement among their scores (Table 2). The mastoid process and the supra-orbital margin had moderate agreement. There was statistically significant agreement between the scores.

**Table 2:** Intra-observer score analysis.

Trait	Kappa value	Significance (p)
Nuchal crest	0.329	<0.001
Mastoid process	0.468	<0.001
Supra-orbital margin	0.408	<0.001
Supra-orbital ridge	0.350	<0.001
Mental eminence	0.298	<0.001

For inter-observer error comparison, of the seven individuals that both observers scored, 87.7 % were scored within one of each other and 22.4 % received the same score. The overall sex estimation outcome was the same in six out of the seven individuals.

### 3.2 Comparison of morphological and proteomic sex estimation

Using Walker's cranial standard [10], 32 of the 34 individuals had sex that was able to be estimated (had a majority of trait scores either under or over a 3), while 32 individuals yielded good peptide results. Full details of amelogenin peak intensity and the spectra associated with peptide results are given in Supplementary Table 1 and Supplementary figures (1–180). An example of both a male and a female result are given in Fig. 1. Two individuals were excluded (7 and 13) as the samples had very low peptide intensity for all forms, shown in Fig. 2. A comparison of osteological and proteomic sex for each individual is given in Table 3.

**Fig. 1:**
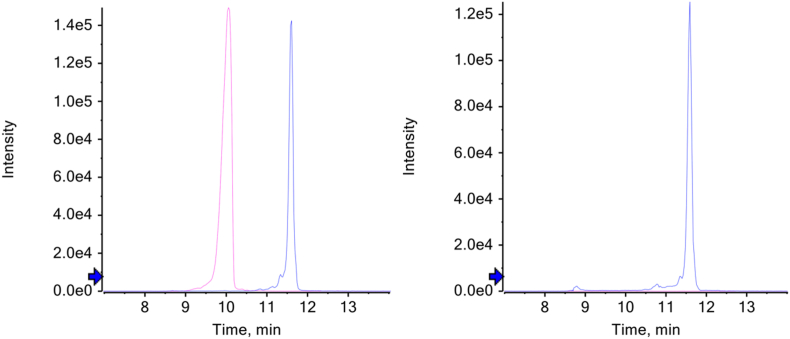
Extracted ion chromatograms of fragment ions y5 at *m*/*z* 645.372 (pink) for SMIRPPY (Y chromosome-linked) and b6 at *m*/*z* 714.393 (blue) for SIRPPYPSY (X chromosome-linked) in sample 41 (right) and sample 44 (left). Sample 44 is male, while sample 41 is female. Blue arrow indicates the intensity threshold for annotating peaks. (For interpretation of the references to color in this figure legend, the reader is referred to the Web version of this article.)

**Fig. 2:**
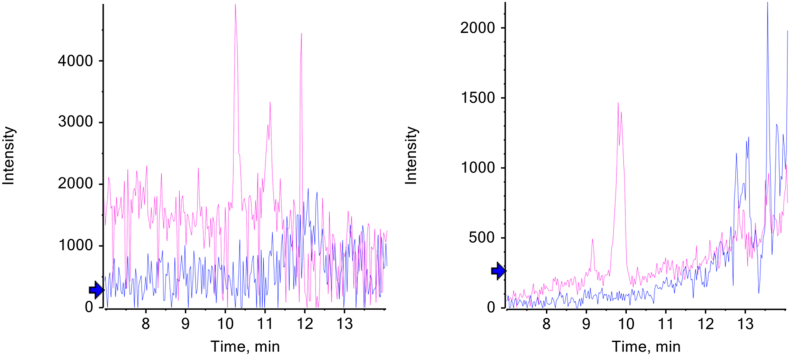
Extracted ion chromatograms at *m*/*z* 440.22 (pink) for S**M**IRPPY (Y chromosome-liked) 540.28 (blue) for SIRPPYPSY (X chromosome-linked) in sample 7 (left) and sample 44 (right). Both individuals were excluded from further analysis as peptide intensity is low. Blue arrow indicates the intensity threshold for annotating peaks. (For interpretation of the references to color in this figure legend, the reader is referred to the Web version of this article.)

**Table 3:** Results of sex estimation.

Individual skeletal ID	Osteological sex estimation [10]	Proteomic sex estimation
1	M	M
2	F	M
4	F	F
6	F	M
7	excluded	–
9	M	M
10	F	M
11	F	F
12	F	M
13	excluded	–
14	F	F
15	M	M
16	F	F
17	indeterminant	M
18	indeterminant	M
19	M	M
20	F	F
21	F	M
22	F	M
26	F	M
31	F	F
32	F	F
36	F	M
38	F	F
39	F	M
40	F	F
41	F	F
42	F	F
43	F	F
44	M	M
46	F	M
54.85	F	F
N/A (SJ)	F	F
N/A (LR)	M	M

Note: indeterminant indicates an individual that did not receive a majority of scores either above or below 3 and therefore sex could not be estimated. Excluded indicates that proteomic sex estimation results were inconclusive.

Individuals for whom no sex could be determined by peptide analysis and/or had an indeterminant sex estimation using Walker [10] were excluded from the sample during further statistical analysis. Morphological estimation was deemed indeterminant when an individual did not receive a majority of scores of either above or below 3. The excluded peptide results, shown in Fig. 2, as noted previously, were excluded due to having low peptide intensity. This left 30 individuals remaining in the sample group.

### 3.3 Walker's standard accuracy for this sample

19 of the 30 (63.33 %) individuals were correctly classified by sex using Walker's standard [10] when using the proteomic analysis results as chromosomal sex. However, this osteological classification accuracy varied depending on chromosomal sex of the individual. Of the 17 males analyzed, only 6 (35.53 %) were classified correctly, whereas all 13 of the females were classified correctly.

Assessment of which traits were most likely to produce an accurate sex estimation indicated that the supraorbital margin was the least accurate trait for sex estimation in this sample and the mental eminence was the most accurate (Table 4).

**Table 4:** Accuracy of sex estimation based on specific osteological traits.

Skull trait = Sex	Correctly classified	Male correctly classified	Female correctly classified	Sex bias
Nuchal crest	68.00 %	33.33 %	100 %	−66.67
Mastoid process	66.67 %	25.00 %	100 %	−75.00
Supra-orbital margin	60.87 %	27.27 %	91.67 %	−64.40
Supra-orbital ridge	80.00 %	62.50 %	100.00 %	−37.50
Mental eminence	84.21 %	66.67 %	100.00 %	−33.33
Multivariate	63.33 %	35.53 %	100.00 %	−64.47

Note: Sex bias indicates the balance in accuracy of sex estimation between males and females (negative value = males more often incorrectly classified). Multivariate indicates the classification accuracy when sex was estimated by observing all trait scores together.

The mean trait scores were statistically different between males and females for all traits (Table 5). All female trait score means are ≤2.3, as the standard would predict for female morphology. However, for 3 of the 5 traits in the male group the mean scores fall under 3, and all values are less than 3.5. This is less than the robust morphology required to be predicted as male using the Walker scoring system [10]. The mean supra-orbital ridge score was 3.3, which while also low on the scoring system, is a result of the wide range of scores for males in the sample group for this trait, which ranged from 1.5 to 5.

**Table 5:** Mean trait scores for males vs. females.

Skull trait	Male score range	Male group mean	Male std. dev	Female score range	Female group mean	Female std. dev	Difference in group means	Sig.
Nuchal crest	1–4.5	2.7	0.976	1–2	1.4	0.400	1.3	<0.001*
Mastoid process	2–4	2.7	0.671	1–3	1.8	0.805	0.9	0.002*
Supra-orbital margin	2–4.5	2.8	0.647	1–3.5	1.9	0.786	0.9	0.001*
Supra-orbital ridge	1.5–5	3.3	0.871	1–3	1.5	0.764	1.8	<0.001*
Mental eminence	2.5–4	3.1	0.436	1–3	2.3	0.560	0.8	<0.001*

Note: * indicates significant differences between male and female group means (p < 0.05, *t*-test).

Male gracility in this sample is supported by the calculation of trait score cut points as per Walker [10]. These scores represent the lowest scores at which males were more likely to be represented than females. Cut points were at 3 or lower for every trait (Table 6).

**Table 6:** Trait frequencies and probabilities of those scores indicating a person was male/female.

Trait/group	Score = 1	Score = 1/2	Score = 2	Score = 2/3	Score = 3	Score = 3/4	Score = 4	Score = 4/5	Score = 5
**Nuchal Crest**									
**Male %**	11.76	5.88	17.65	**17.65**	29.41	5.88	11.76	5.88	0.00
**Female %**	23.08	38.46	23.08	**0.00**	0.00	7.69	0.00	0.00	0.00
**Male probability**	0.33	0.13	0.43	**1.00**	1.00	0.43	1.00	1.00	–
**Female probability**	0.66	0.87	0.57	**0.00**	0.00	0.57	0.00	0.00	–
**Mastoid Process**									
**Male %**	0.00	0.00	**41.18**	0.00	52.94	0.00	11.76	0.00	0.00
**Female %**	38.46	7.69	**38.46**	0.00	23.08	0.00	0.00	0.00	0.00
**Male probability**	0.00	0.00	**0.52**	–	0.70	–	1.00	–	–
**Female probability**	1.00	1.00	**0.48**	–	0.30	–	0.00	–	–
**Supra-orbital Margin**									
**Male %**	0.00	0.00	23.53	**29.41**	35.29	17.65	0.00	5.88	0.00
**Female %**	30.77	0.00	46.15	**0.00**	15.38	7.69	0.00	0.00	0.00
**Male probability**	0.00	–	0.34	**1.00**	0.70	0.70	–	1.00	–
**Female probability**	1.00	–	0.66	**0.00**	0.30	0.30	–	0.00	–
**Supra-orbital ridge**									
**Male %**	0.00	5.88	11.76	0.00	**47.65**	17.65	5.88	5.88	11.76
**Female %**	61.54	7.69	15.38	0.00	**23.08**	0.00	0.00	0.00	0.00
**Male probability**	0.00	0.43	0.43	–	**0.67**	1.00	1.00	1.00	1.00
**Female probability**	1.00	0.57	0.57	–	**0.33**	0.00	0.00	0.00	0.00
**Mental Eminence**									
**Male %**	0.00	0.00	0.00	17.65	**52.94**	23.53	11.76	0.00	0.00
**Female %**	7.69	0.00	38.46	30.77	**30.77**	0.00	0.00	0.00	0.00
**Male probability**	0.00	–	0.00	0.34	**0.63**	1.00	1.00	–	–
**Female probability**	1.00	–	1.00	0.66	**0.37**	0.00	0.00	–	–

Note: The ‘cut point’ scores (the point at which the probability of a score being indicative of female switches to male) for each score are bolded.

The application of the population-specific discriminant function equations created by Walker [10] are shown in Table 7. In most instances, these equations created higher overall correct classification rates (53.12 %–93.75 %) in comparison with the initial classification rate of 63.33 % (Table 4). Sex bias (a measure of accurate classification equality between the sexes) generally reduced with the application of discriminant function equations in comparison with the sex bias observed when using all traits as indicators with no weighting (64.47 %, table). This indicates that these equations can be useful as a method of introducing some population specificity to the standard by weighting traits.

**Table 7:** Results of sex estimation using population-specific discriminant function equations [10].

Discriminant function	Total correctly classified (%)	Males correctly classified (%)	Females correctly classified (%)	Sex bias (%)
**American/English population-based equations**				
Y = glabella X -1.375 + mastoid X -1.185 + mental X -1.151 + 9.128	90.62	94.74	84.61	10.12
Y = glabella X -1.568 + mastoid X -1.459 + 7.434	90.62	94.74	84.61	10.12
Y = glabella X -1.525 + mental X -1.485 + 7.372	93.75	94.74	92.31	2.43
Y = mental X -1.629 + mastoid X -1.415 + 7.382	84.37	94.74	69.23	25.51
Y = orbit X -1.007 + mental X -1.850 + 6.018	78.12	100.00	46.15	53.85
Y = orbit X -0.7 + mastoid X -1.559 + 5.329	71.87	68.42	76.92	−8.50
**Native American population-based equations**				
Y = orbit X -0.499 + mental X -0.606 + 3.414	53.12	21.05	100.00	−78.95
Y = mental X -0.576 + mastoid X -1.136 + 4.765	71.87	57.89	92.31	−34.41
Y = glabella X -0.797 + mastoid X -1.085 + 5.025	93.75	94.74	92.31	2.43

Note: Sex bias = % males correctly classified - % females correctly classified.

## 4 Discussion

For this South Asian skeletal collection, the Walker [10] sex estimation standard provided an overall accuracy of 63.33 %. This is lower than the classification rates documented by Walker when applied to Native American, African American, European American, and English individuals (69–83 %) [10]. In terms of predicting a binary outcome, our results certainly reflect a low percentage of accuracy. Sex bias was considerably higher in this South Asian sample group (−33.33 to −75.00) than reported by Walker [10] for American/English individuals (5.5 to −19.9).

The high sex bias and low accuracy of classification using this method appears to be due to the high misclassification of males. Analysis of trait scores indicates that this misclassification reflects the more gracile expression of traits by males in the group than considered indicative of stereotypically male morphology by Walker's standard [10]. The trait with the highest mean score for males in the study sample was the supra-orbital ridge with a mean score of 3.3. This shows that most males from this sample had scores less than the 4 or 5 required for a sex estimation to indicate male. Less sexual dimorphism compared with Walker's standard [10] was likely also a factor in the reduced accuracy of the standard as males and females were often both scored on the more gracile end of the standard's scale, reducing the discriminatory power of the scoring method.

Reduced sexual dimorphism has been identified in some Southeast Asian populations as a cause of inaccurate sex estimation [24], and similar low levels of accuracy have been documented in various other studies of non-White, non-American/European populations [24,41,42]. This study therefore adds to the literature highlighting the inadequacies of non-representative standards for sex estimation. The question remains as to what is an acceptable level of accuracy is for osteological sex estimation methods. Previous assessments of the Walker sex estimation standard by Jilala et al. [42] in Tanzania and Krüger et al. [41] in South Africa have suggested that their observed accuracy rates of as low as 47 % and 68 % respectively and demonstrate that the technique is not fit for purpose. Following the findings of Jilala et al. [42] and Krüger et al. [41], and based on our study, we suggest that the Walker standard [10] also needs to be used with caution in South Asian populations. The potential misclassification of sex is particularly relevant for the field of forensics, where knowledge of the accuracy and associated error associated with techniques is paramount for admissibility in court [22].

Both intra- and inter-observer tests indicated that the mental eminence had the lowest agreement despite being the most accurate trait for indicating sex. The nuchal crest showed the second lowest intra-observer agreement and was the second lowest in sex prediction accuracy, however, this trait had the second highest inter-observer agreement. Both assessor bias and experience as well as ambiguous descriptions are likely causes of some Walker standard [10] traits to be more prone to observer disagreement [81]. Lewis and Garvin (2016) found that in their sample population of U.S. Black and White individuals, the mental eminence had the lowest inter-observer agreement. For their study the mental eminence also had the lowest intra-observer agreement for one observer while having the highest agreement for another. Similarly in this study, the mental eminence had the lowest intra-and inter-observer agreement. This demonstrates the inherent limitations of observational based methods [81].

### 4.1 Where to from here?

From these results we suggest that if the Walker standard [10] is to be used for sex estimation on populations where it has been shown to produce less accurate results, adaptations need to be made to ensure increased accuracy. Using a weighting system where traits that provide the most accuracy for sex estimation in a given population are given more weight may aid in this. However, any weighting systems used would need to be population specific as there is variation in trait accuracy among different populations [10,[18], [19], [20], [21], [22],^27^]. For example, previous research shows that in ‘gracile populations’ not all skeletal morphology traits are relatively more gracile. Instead, some traits may be more gracile (relative to Walker's standard) while others may be closer to what the standard predicts which is reflected in varying trait accuracy between populations (Table 8). This can be seen in studies looking at Thai and Filipino individuals which indicated cut points between male and female scores were less than 3 in every trait except for the mastoid process where the cut point was 3 [24].

**Table 8:** Skull morphological traits ranked in order of classification accuracy for various populations.

Population (as described by publications)	Most accurate (1) to least accurate (5) predictor trait in the study					Study notes	Combined trait accuracy
	1	2	3	4	5		
South Asian (this study)	Mental eminence (84.21 %)	Supra-orbital ridge (80.00 %)	Nuchal crest (68.00 %)	Mastoid process (66.67 %)	Supra-orbital margin (60.87 %)		63.33 %
American/English) [10]	Supra-orbital ridge (82.6 %)	Mastoid process (78.6 %)	Mental eminence (76.6 %)	Nuchal crest (71.4 %)	Supra-orbital margin (68.8 %)		88 %
Medieval English [18]	Mental eminence (72.7 %)	Supra-orbital ridge (69.2 %)	Mastoid process (63.5 %)	Nuchal crest (62.9 %)	Supra-orbital margin (57.9 %)		76.7 %
U.S. Blacks, U.S. Whites, Medieval Nubians & Native American [19]	Supra-orbital ridge & mastoid process noted as most reliable (no values given)		–	–	Nuchal crest noted as least accurate (no values given)		–
Japanese [27]	Supra-orbital ridge (76.94 %)	Supra-orbital margin (71.53 %)	Mental eminence (61.57 %)	Nuchal crest (56.54 %)	Mastoid process (55.91 %)		69.26 %
Thai [27]	Supra-orbital ridge (79.12 %)	Mastoid process (72.32 %)	Mental eminence (66.90 %)	Supra-orbital margin (65.93 %)	Nuchal crest (64.01 %)		69.70 %
Brazilian [20]	Supra-orbital ridge (80.73 %)	Mastoid process (73.44 %)	Supra-orbital margin (72.92 %)	Mental eminence (70 %)	*-*	*Nuchal crest not observed*	*Traits observed only individually*
African American [21]	Supra-orbital ridge (82.52 %)	Mastoid process (76.70 %)	Mental eminence (72.81 %)	Nuchal crest (70.87 %)	Supra-orbital margin (52.43 %)		88.34 %
European American [21]	Supra-orbital ridge (80.58 %)	Mastoid process (79.61 %)	Mental eminence (77.67 %)	Nuchal crest (74.76 %)	Supra-orbital margin (72.81 %)		87.38 %
English [21]	Supra-orbital ridge (85.91 %)	Mastoid process (81.69 %)	Mental eminence (80.28 %)	Nuchal crest (73.24 %)	Supra-orbital margin (67.60 %)		94.37 %
Modern White European [22]	Mastoid process (92 %)	Supra-orbital ridge (86 %)	Supra-orbital margin (76 %)	–	–	*Nuchal crest & mental eminence not observed*	*Traits observed only individually*
South African [23]	Supra-orbital ridge (60.29 %)	Mastoid process (54.07 %)	Mental eminence (53.85 %)	Supra-orbital margin (48.20 %)	Nuchal crest (48.15 %)		*57.78 %*

Discriminant function equations are a way of factoring in the effect of population variation of different morphological traits. Walker's publication included the creation of logistic discriminant function equations as a way of combining the standard with multivariate statistics that weigh the best sex predictors for the reference sample [10]. In this study our small sample size precluded the creation of population specific discriminant function equations, however, we noted that application of equations created for American/English and Native American populations did increase accuracy and decrease sex bias. Therefore, it is likely that the creation of South Asian specific discriminant function equations based on larger scale observations would produce greater accuracy in sex-classification. As large collections of known sex skeletal data, preferably with well documented ancestry to account for variation, are required for this, the ability to create discriminant function equations is limited. Other methods of introducing population specificity include the optimized summed scored attributes (OSSA) method, a technique created for ancestry estimation and later applied to sex estimation [26,86]. The method works by creating sectioning points based on trait score frequency distribution in a known sex sample group which can then be applied to unknown sex individuals [26]. Large sample groups are required to produce population specific OSSA scores; however, we applied the scores generated by Tallman & Go's (2018) based on Japanese and Thai samples and found accuracy rates of 93.75 % and 90.25 % respectively. The accuracy of using Tallman & Go's (2018) scores was not any higher than the application of Walker's (2008) American/English or Native American derived discriminant function equations (93.75 %). This suggests that more population specific data is required than simply the application of wider Asia-based sample groups [10]. With its large geographical area and diverse selective pressures, it is unsurprising that we see this variation between populations within Asia [87].

### 4.2 Could proteomic sex estimation become the norm?

Although the use of proteomic analysis for sex estimation negates the impact of population variation seen in morphological based methods, this technique is both more laborious and requires access to costly technology. The establishment of collaborative relationships between institutions who possess the technology required for this analysis would allow for greater accessibility. However, in contexts with many unidentified individuals the cost of analysis may preclude the use of peptide-based sex determination. In these contexts, perhaps a combined approach, with initial osteological analysis undertaken to estimate sex, and follow-up proteomic analysis to ground-truth results and/or provide insight into the sex of individuals with more intermediate morphology may be appropriate.

The major benefit of proteomic analysis is its reliability [57]. However, as with all scientific techniques there are some known limitations that may impact results. Variations in peptide intensity between samples is expected, however, in some cases intensity can be very low (particularly for historic or taphonomically altered skeletal material). Low intensity may cause AMELY false negatives should levels not exceed detection threshold. This problem can be avoided using stable isotope labeling. Labeling is an established method of relatively quantifying peptides within a sample, by measuring absolute amounts and estimating the limit of detection [[88], [89], [90]]. A secondary limitation of proteomic analysis is amino acid modification, a technical error that can impact result outcome [91]. Methionine is a readily oxidizable amino acid and can be erroneously oxidized during sample preparation [92]. In certain amylogenic isoforms, methionine is present in an oxidized state while in others it is not. This accidental oxidation reaction may be the cause for some variation in the spectra results for the target AMELY SMIRPPY (−432.2258++). However, during this analysis multiple amino acid sequences are targeted for each peptide to ensure a result redundancy. Lastly, genetic mutations resulting in isoform deletion may impact spectra results. There have been documented cases of AMELY allele deletion in dental enamel which is problematic for this analysis as it would cause males to be classified as females. AMELY deletion has a reported prevalence of up to 10 % in Indian subcontinent populations, though the factuality of this has been debated [56,93]. It is therefore worth considering that the number of male individuals may be underestimated when the technique is applied to South Asian populations. AMELX allele deletions have also been documented in South Chinese populations, though with a very low frequency (0.037 %) [94,95]. This deletion would exclude an individual from analysis.

Although enamel peptide analysis is considered a destructive method it requires only a minute amount of enamel, with the etching of the tooth surface often invisible to the naked eye. Because of this we feel peptide analysis is highly advantageous in getting accurate sex results with minimal tissue loss, compared with aDNA analysis that requires destruction of a small amount of bone or tooth tissue. Despite this, implementation of any kind of destructive method, no matter how minimally invasive should require cultural consultation for any context.

### 4.3 Future research for morphological sex estimation

When considering future development of morphological sex estimation methods, there are a number of issues that must be addressed. Collections such as those used in this research are the subject of increasing ethical scrutiny and debate as to whether human remains collected historically without consent should still be used for teaching, research, and/or on display [28,68,96].

We acknowledge that the historic nature of skeletal collections (such as the one used in this study) may also mean that data is geographically limited and may not be strongly representative of contemporary remains as variation in sexual dimorphism can develop within small timeframes [10,97]. A future step would therefore be to undertake more data collection drawing upon a temporally and geographically wider sample in order to attempt to mitigate these limitations. Alternative methods of generating morphological data may help to address this issue. The increased accessibility of data from living peoples through medical imaging such as magnetic resonance imaging (MRI), could allow for large non-metric datasets to be obtained. While collecting MRI data from contemporary populations would be more costly than the use of skeletal collections, it may provide useful additional data from which population-specific standards can be developed. Work of this kind has already been successfully attempted with mental eminence expression [98]. Use of MRI would, by necessity, be limited to areas where the technology and resources are available. This may create sample biases; however, live imaging of skeletal morphology would negate the use of skeletal collections that may be associated with ethical challenges (notwithstanding the need for informed consent for modern human subject research) while allowing for more diverse people groups to be observed, including those where there are few or no known accessible skeletal collections available.

In addition to the testing of established standards, this study also had another aim; to add to our understanding of the people who make up the legacy collection in the W.D. Trotter Museum. This study provides the foundation for unveiling these peoples’ stories and understanding sex is an important first step in this process. While sex is not necessarily equivalent to gender, knowing chromosomal sex allows us to start thinking about the skeletal remains as people with biological attributes and identities.

As discussed in the introduction, continuing to work with and undertake research on historical collections is an ethically ambiguous area. There are those who advocate for the wholesale reburial and/ordestruction of these remains [66,99], while others argue that the reburial or destruction of these remains is also ethically difficult due to our uncertainty over where these people originated from and their own cultural/religious views on the body [68]. We do not have the answers to these complex issues, but we do believe that treating these people with respect, and acknowledging their humanity should be the foundation of everything we do. Ultimately, we aim to fully reconstruct these people's lives using biological evidence from their skeletons, so that while they are within our care we can consider them as real people, much as we do the cadaveric and skeletal material from our donors in the present day.

## 5 Conclusion

This research has identified discrepancies between osteological sex estimation from a central skull morphological sex estimation standard [10] and proteomic sex estimation in a South Asian skeletal collection. Chromosomal males were often incorrectly classified following the standard, because they generally presented with more gracile trait expression than predicted by the standard. These results align with previous literature assessing the accuracy of the standard when applied to other populations and suggest that more needs to be done to address the lack of representation in morphology-based analysis methods. Proteomic analysis provides an innovative and relatively straightforward way in which sex can be determined without the issue of bias associated with application of osteological standards. However, we acknowledge that it may not be realistically available in some contexts. Further research should focus on gathering more population-specific data on sexually dimorphic trait expression. This would allow either the creation of population specific standards or discriminant function equations which could be applied to better represent the diversity of human sexual dimorphism.

## Ethics statement

Approval from the Department of Anatomy Body Ethics Committee was gained prior to the commencement of this research.

## CRediT authorship contribution statement

**Laura M. Rogers:** Data curation, Formal analysis, Investigation, Methodology, Writing – original draft, Writing – review & editing. **Siân E. Halcrow:** Conceptualization, Investigation, Methodology, Project administration, Supervision, Writing – review & editing. **Torsten Kleffmann:** Methodology, Writing – review & editing. **Charlotte L. King:** Conceptualization, Methodology, Resources, Supervision, Writing – review & editing.

## Declaration of competing interest

The authors declare that they have no known competing financial interests or personal relationships that could have appeared to influence the work reported in this paper.
