# Supplementary material for: Comparing Walker's (2008) skull trait sex estimation standard to proteomic sex estimation for a group of South Asian individuals
Source: Forensic Sci Int Synerg. 2024 Jan 2;8:100450. doi: 10.1016/j.fsisyn.2023.100450 (PMC10837481; doi:10.1016/j.fsisyn.2023.100450)
Supplement: Multimedia component 1 [file mmc1.docx]

**Table 1. Amelogenin isoform peak intensity**

| INDIVIDUAL | AMELY SMIRPPYS - 483.7393++ | AMELY SMIRPPY - 432.2258++ | AMELY SMIRPPY - 440.2233++ | AMELX SIRPPYPSY - 540.2796++ | AMELX SIRPPYP - 415.2320++ | AMELX YEVLTPLK - 481.7815++ | AMELXY LPPHPGHPGYINF - 723.8619++ | AMELXY TPLKWYQ - 468.7449++ |
| --- | --- | --- | --- | --- | --- | --- | --- | --- |
| 1 | 874824.63 | 1590690.88 | 3478992.00 | 1887540.88 | 12101488.00 | 3805364.50 | 502117.50 | 393424.84 |
| 2 | 586815.13 | 1951215.75 | 2872249.50 | 1681005.38 | 10396624.00 | 3422929.50 | 762425.00 | 462191.66 |
| 4 | 950.07 | 273.86 | 18224.20 | 1714283.13 | 6034242.00 | 3471229.00 | 294221.00 | 301325.13 |
| 6 | 434061.81 | 75156.09 | 2401119.00 | 1841832.50 | 7868828.50 | 2922844.00 | 897948.63 | 215663.02 |
| 7 | 772.61 | 52.32 | 13317.46 | 292.21 | 111.48 | 26.70 | 922.03 | 248.16 |
| 9 | 584108.69 | 435608.50 | 1522700.00 | 945099.88 | 6426667.00 | 2509988.50 | 29139.20 | 155787.56 |
| 13 | 495.24 | 21.72 | 5283.69 | 317.66 | 4187.96 | 2775.67 | 195.83 | 23.49 |
| 14 | 8230.63 | 18992.14 | 3133.06 | 234502.70 | 2096506.00 | 1304737.25 | 80695.21 | 19353.89 |
| 15 | 144543.23 | 109.10 | 1022816.13 | 301830.31 | 2379940.00 | 1393561.00 | 12051.66 | 2688.20 |
| 16 | 1087.50 | 168.05 | 5788.18 | 1381642.75 | 5710697.00 | 3059654.00 | 237522.38 | 6339.97 |
| 17 | 109632.88 | 8208.37 | 427078.13 | 99318.62 | 900382.63 | 508605.50 | 2345.95 | 489.22 |
| 20 | 904.27 | 157.95 | 12917.03 | 1357455.38 | 4720092.00 | 2780562.25 | 408631.72 | 210606.28 |
| 21 | 10092.79 | 659.24 | 47668.84 | 9702.35 | 27585.47 | 9934.72 | 2811.50 | 329.95 |
| 22 | 424528.44 | 25458.32 | 1714225.13 | 597596.38 | 3836535.25 | 1979173.88 | 387677.31 | 1740.07 |
| 26 | 446661.72 | 9852.80 | 2185767.00 | 728205.63 | 7315608.00 | 2454880.75 | 203681.95 | 1567.07 |
| 36 | 639667.38 | 57699.46 | 1643784.00 | 639221.50 | 5602626.00 | 2753762.00 | 2092827.88 | 6509.72 |
| 38 | 2655.50 | 727.22 | 9632.08 | 1587647.25 | 9128430.00 | 3103972.25 | 188853.19 | 191664.80 |
| 39 | 545803.13 | 228509.16 | 2006913.88 | 409358.94 | 4423161.00 | 2394783.75 | 6772.90 | 87068.23 |
| 40 | 744.51 | 4182.41 | 22686.04 | 2468275.25 | 11795402.00 | 3758711.75 | 1860010.13 | 362779.13 |
| 41 | 69.74 | 1219.50 | 13903.61 | 2132607.00 | 12396242.00 | 4690838.50 | 627778.63 | 229484.83 |
| 42 | 1100.96 | 880.88 | 8491.72 | 1406819.13 | 9222843.00 | 2730989.00 | 318065.09 | 162157.42 |
| 44 | 805175.63 | 2443.29 | 4250109.00 | 2484323.25 | 9913714.00 | 2771690.00 | 831176.75 | 2523.48 |
| 46 | 638940.38 | 825007.38 | 2204445.00 | 720352.69 | 5892315.00 | 3659273.75 | 847663.50 | 187410.75 |
| 54.5 | 522.51 | 2375.05 | 14009.50 | 402637.41 | 2497369.00 | 1455001.00 | 378728.72 | 32922.01 |
| LR | 612285.31 | 421706.97 | 3066872.00 | 1359421.75 | 5397980.00 | 3001938.50 | 729800.13 | t305341.  50 |
| SJ | 702335.44 | 171997.94 | 2015065.25 | 469084.59 | 6338319.00 | 2832528.50 | 100748.34 | 101810.48 |

**AMELY Ion chromatograms:**

**Figure 1. Ion chromatogram of amino acid sequence SMIRPPYS - 483.7393++ (AMELY), individual 1.**


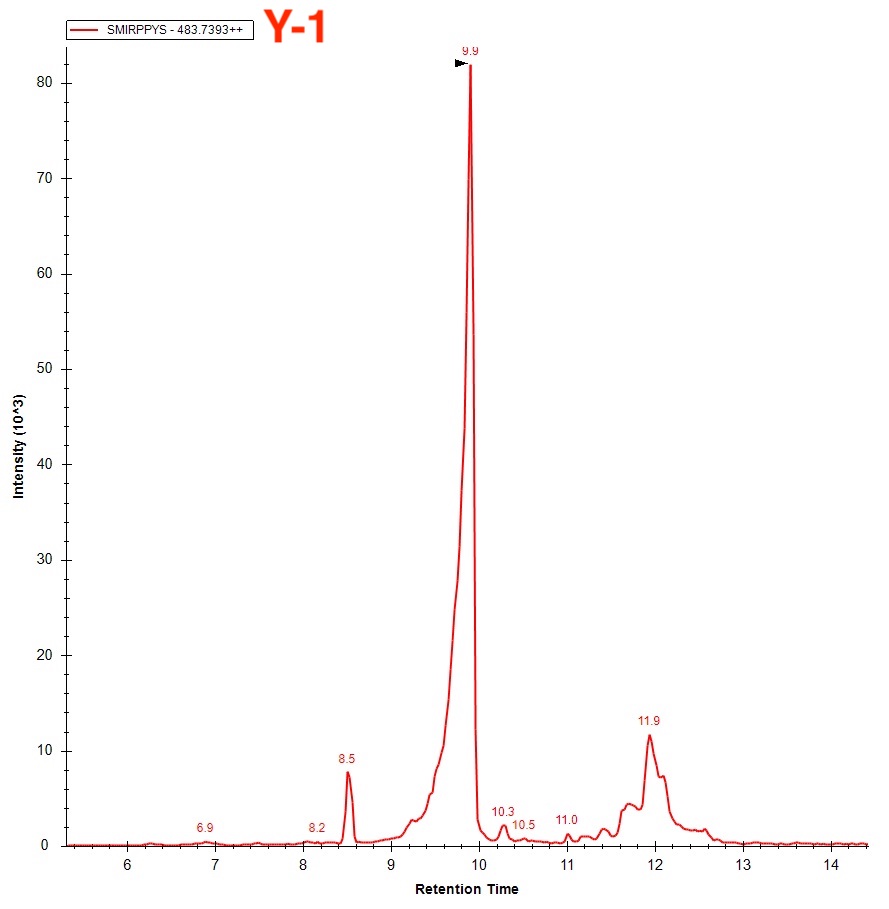


**Figure 2. Ion chromatogram of amino acid sequence SMIRPPYS - 483.7393++ (AMELY), individual 2.**
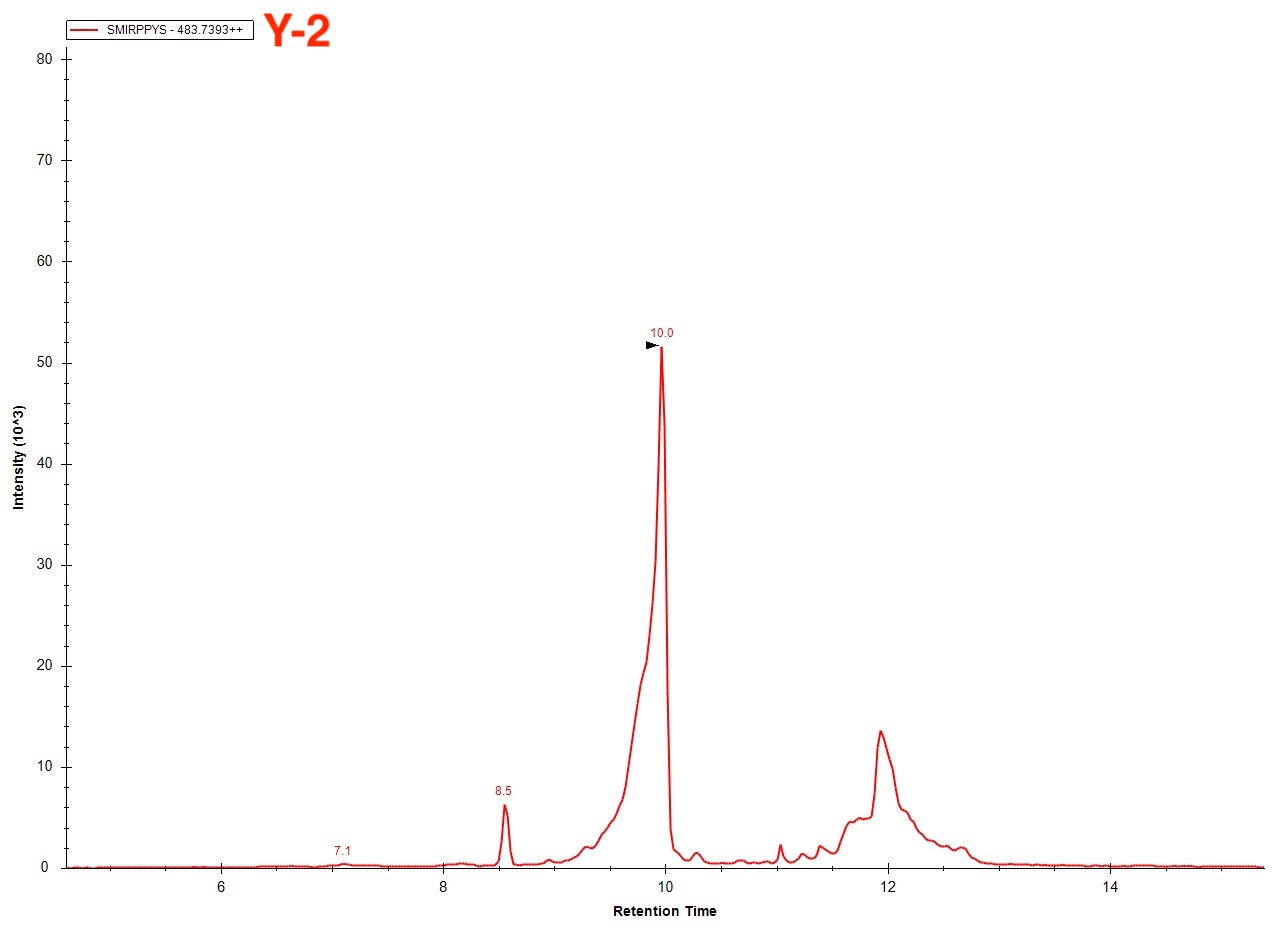


**Figure 3. Ion chromatogram of amino acid sequence SMIRPPYS - 483.7393++ (AMELY), individual 4.**
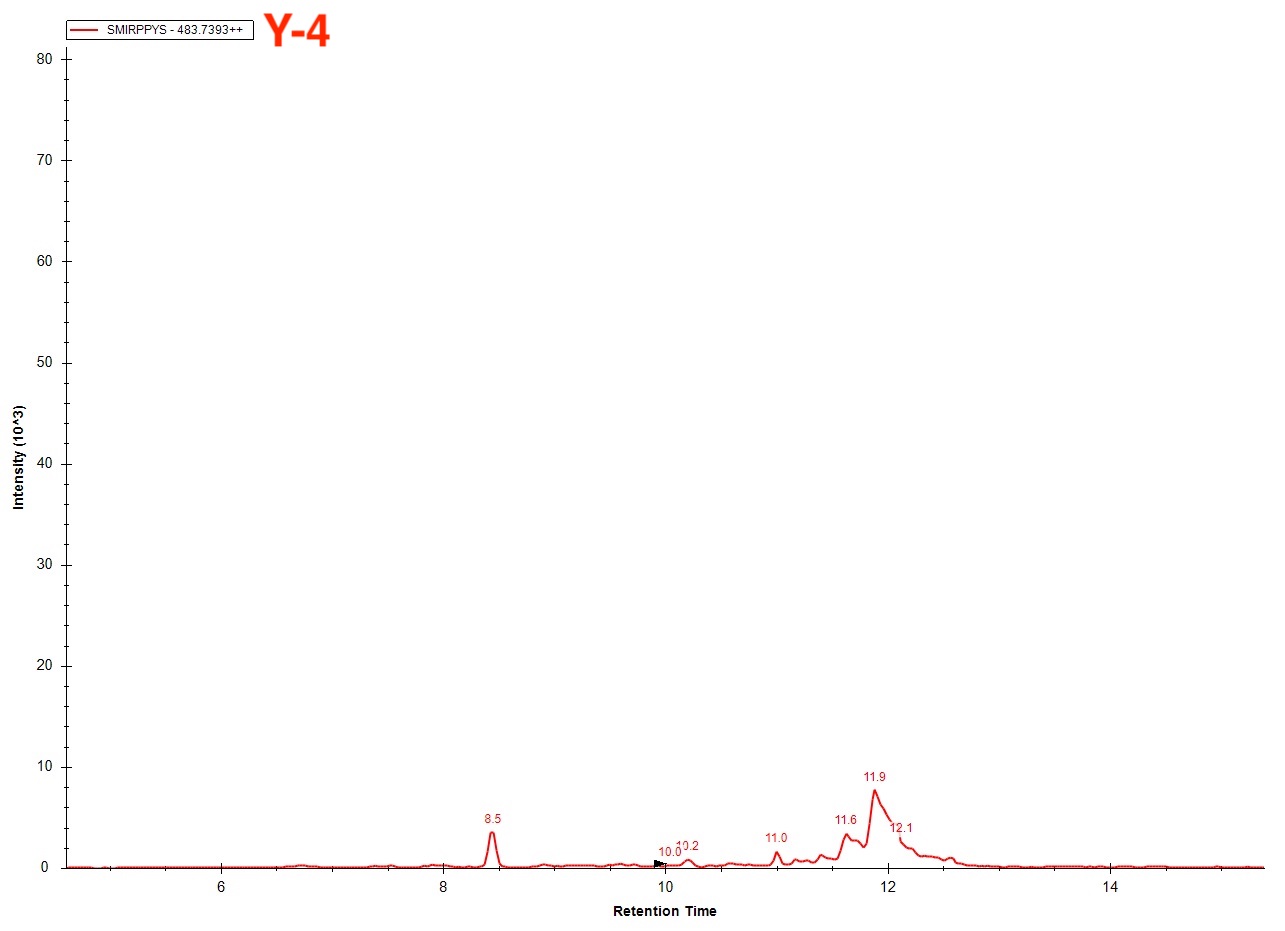


**Figure 4. Ion chromatogram of amino acid sequence SMIRPPYS - 483.7393++ (AMELY), individual 6.**
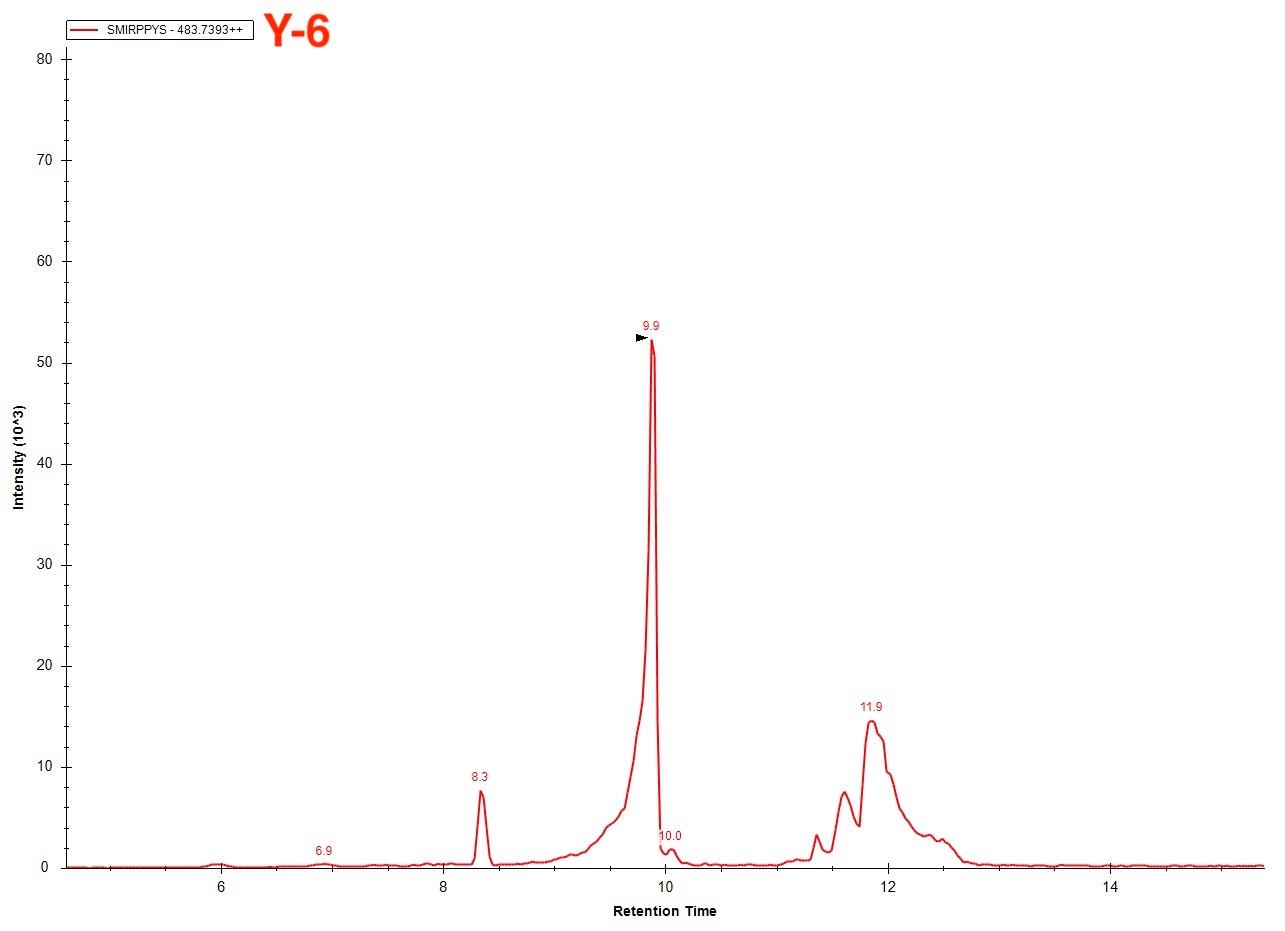


**Figure 5. Ion chromatogram of amino acid sequence SMIRPPYS - 483.7393++ (AMELY), individual 7.**
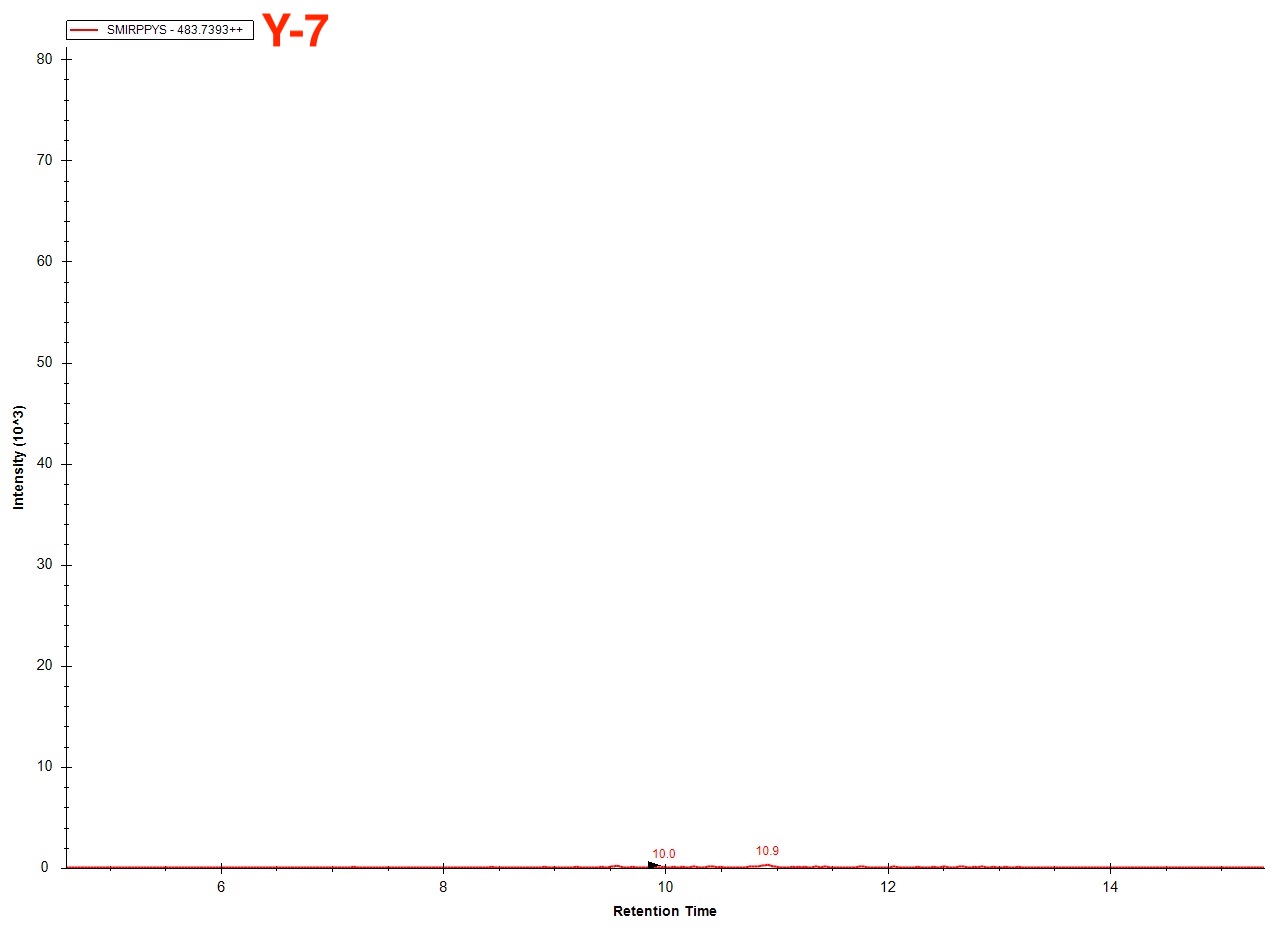


**Figure 6. Ion chromatogram of amino acid sequence SMIRPPYS - 483.7393++ (AMELY), individual 9.**


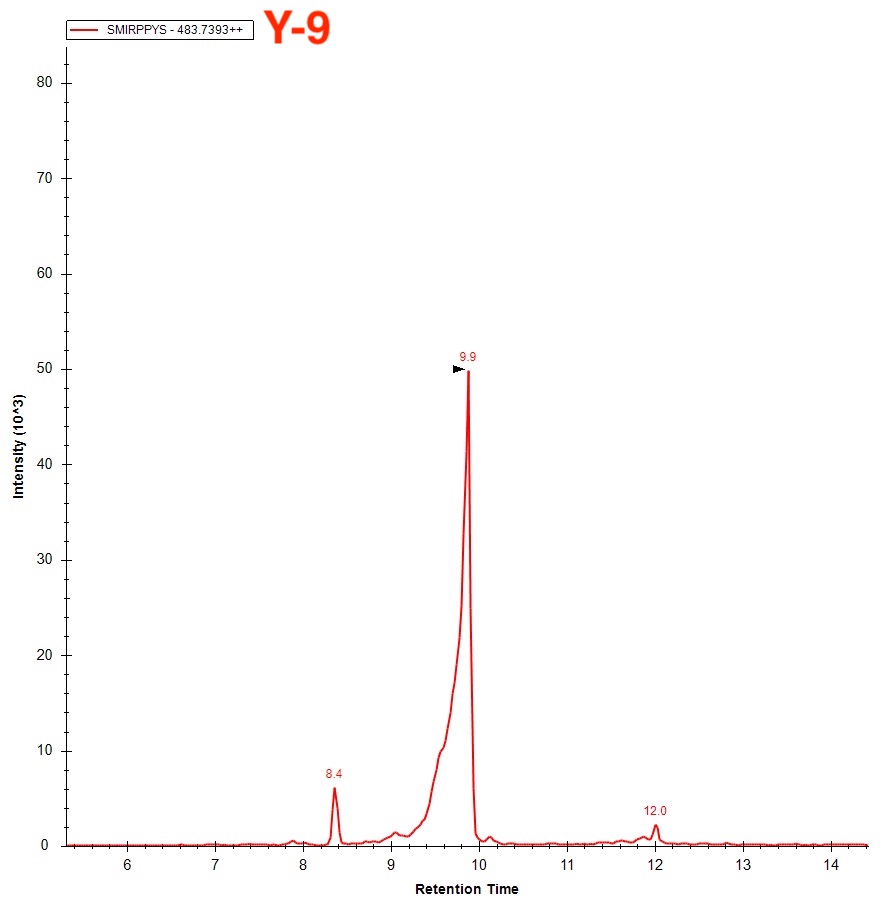


**Figure 7. Ion chromatogram of amino acid sequence SMIRPPYS - 483.7393++ (AMELY), individual 13.**


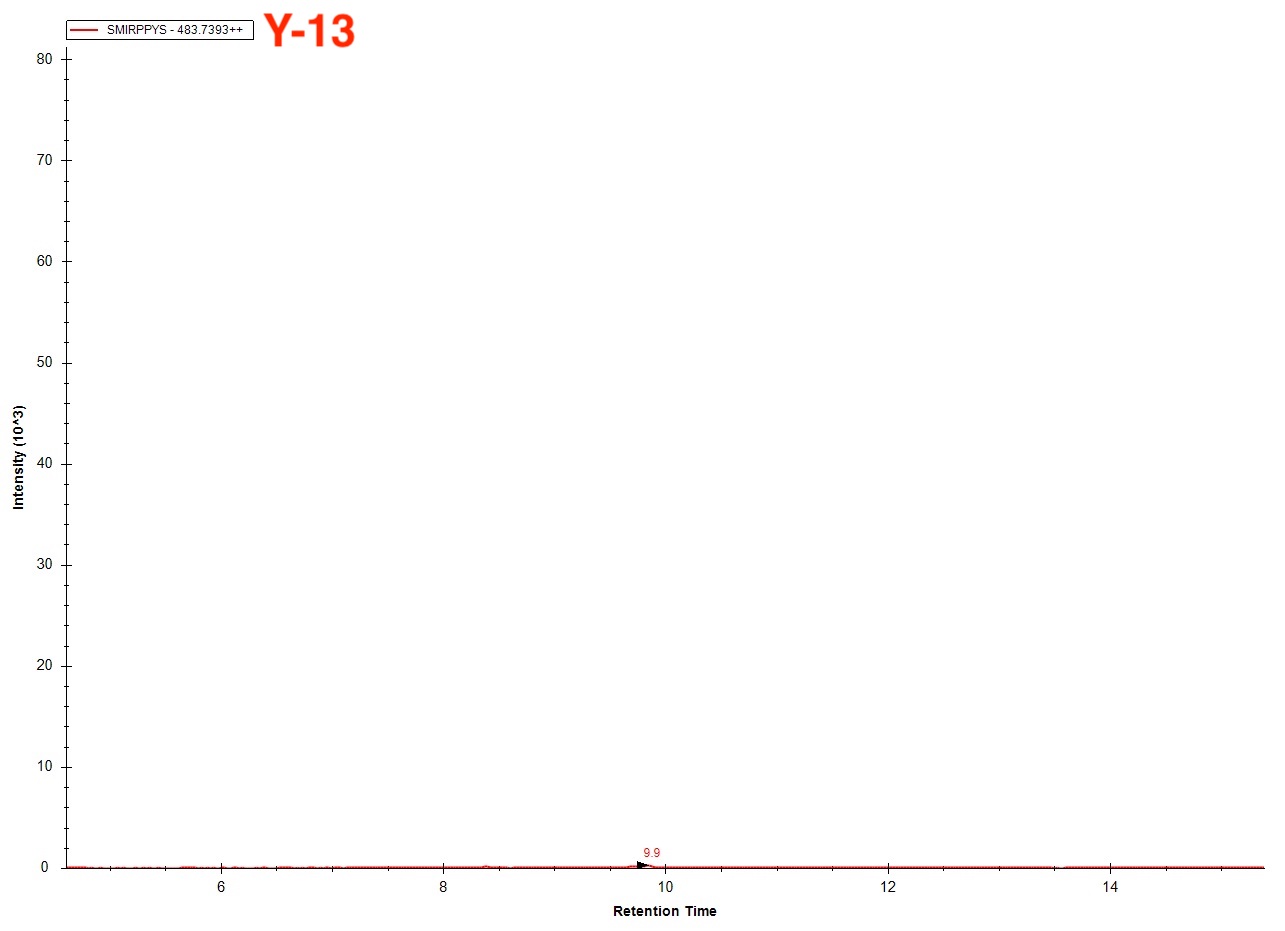


**Figure 8. Ion chromatogram of amino acid sequence SMIRPPYS - 483.7393++ (AMELY), individual 14.**


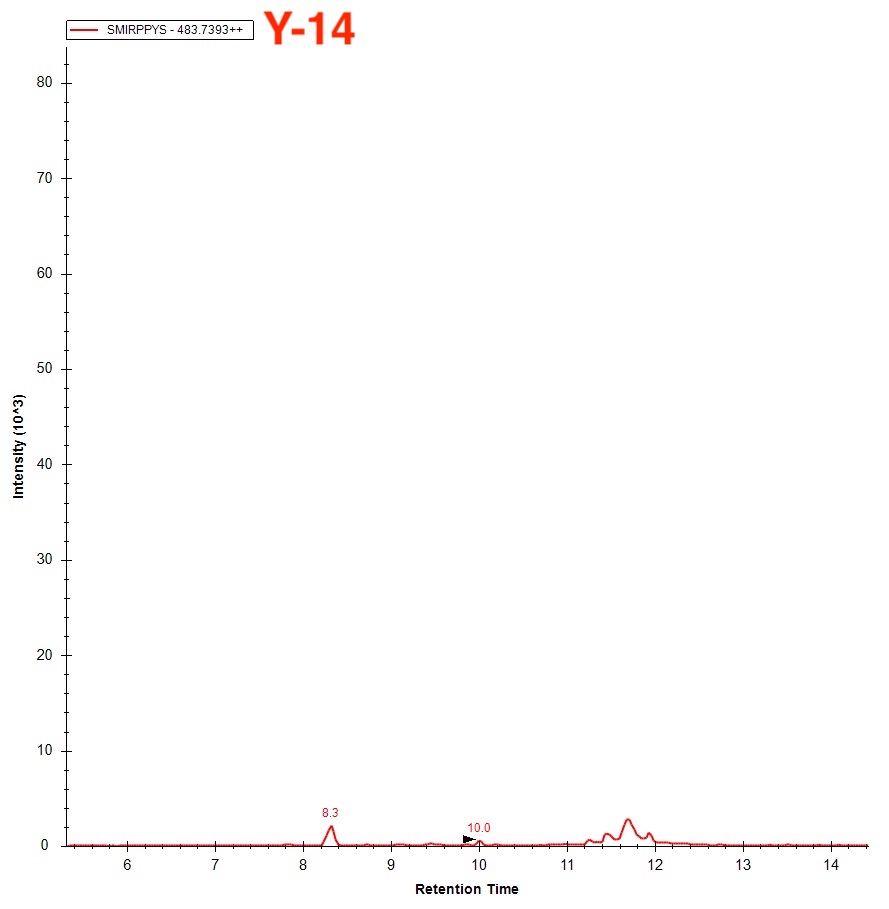


**Figure 9. Ion chromatogram of amino acid sequence SMIRPPYS - 483.7393++ (AMELY), individual 15.**
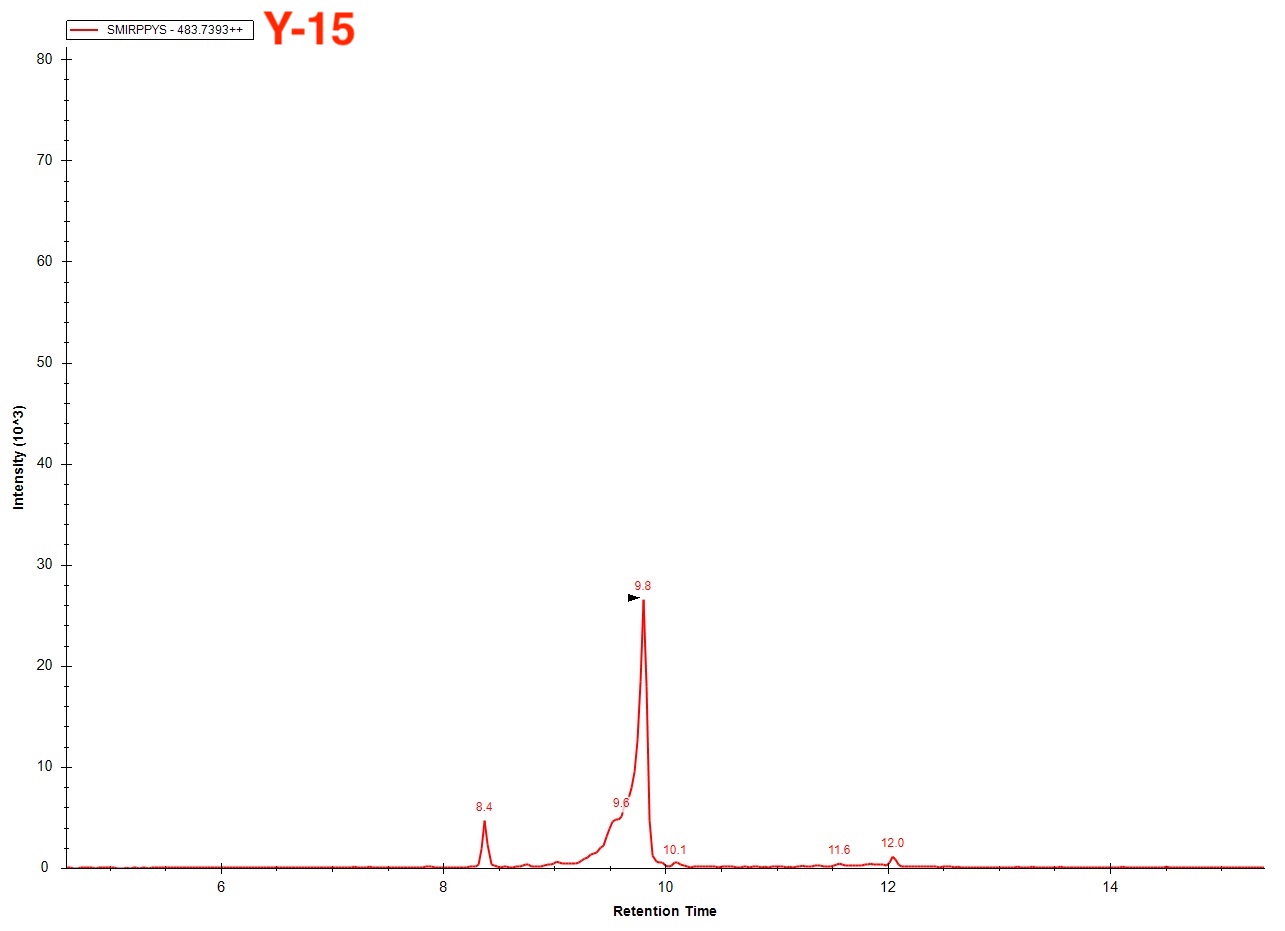


**Figure 10. Ion chromatogram of amino acid sequence SMIRPPYS - 483.7393++ (AMELY), individual 16.**
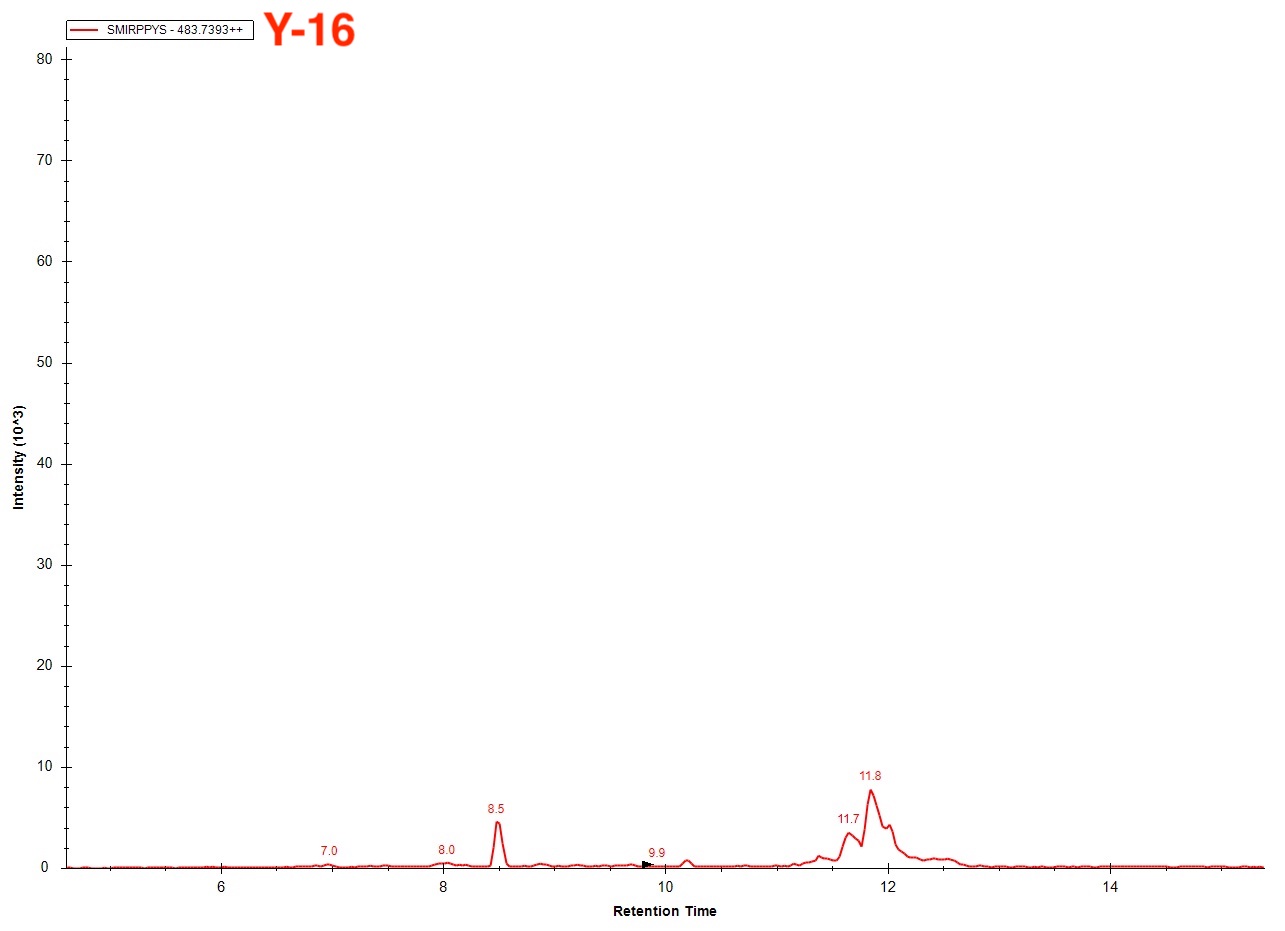


**Figure 11. Ion chromatogram of amino acid sequence SMIRPPYS - 483.7393++ (AMELY), individual 17.**
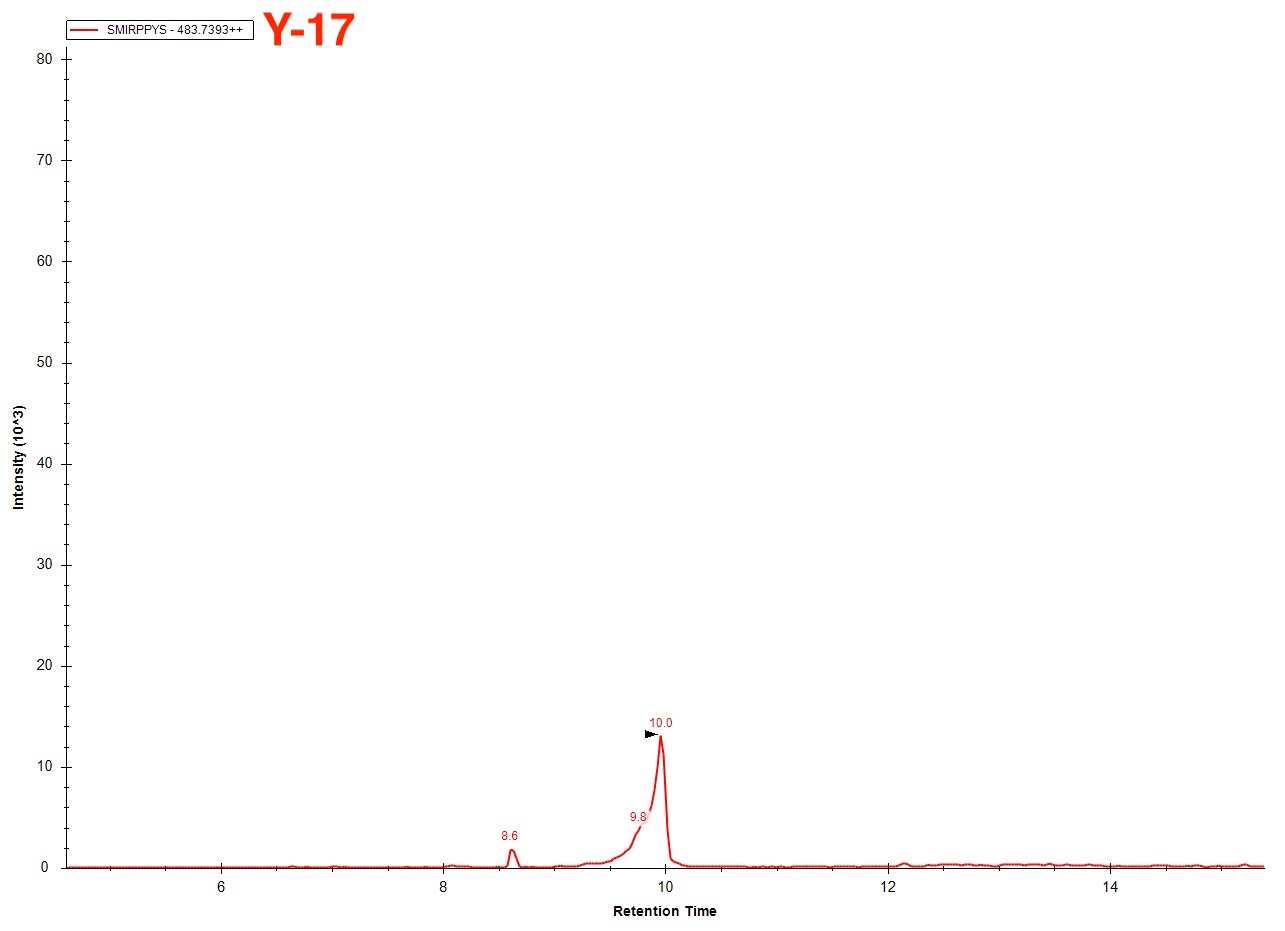


**Figure 12. Ion chromatogram of amino acid sequence SMIRPPYS - 483.7393++ (AMELY), individual 20.**
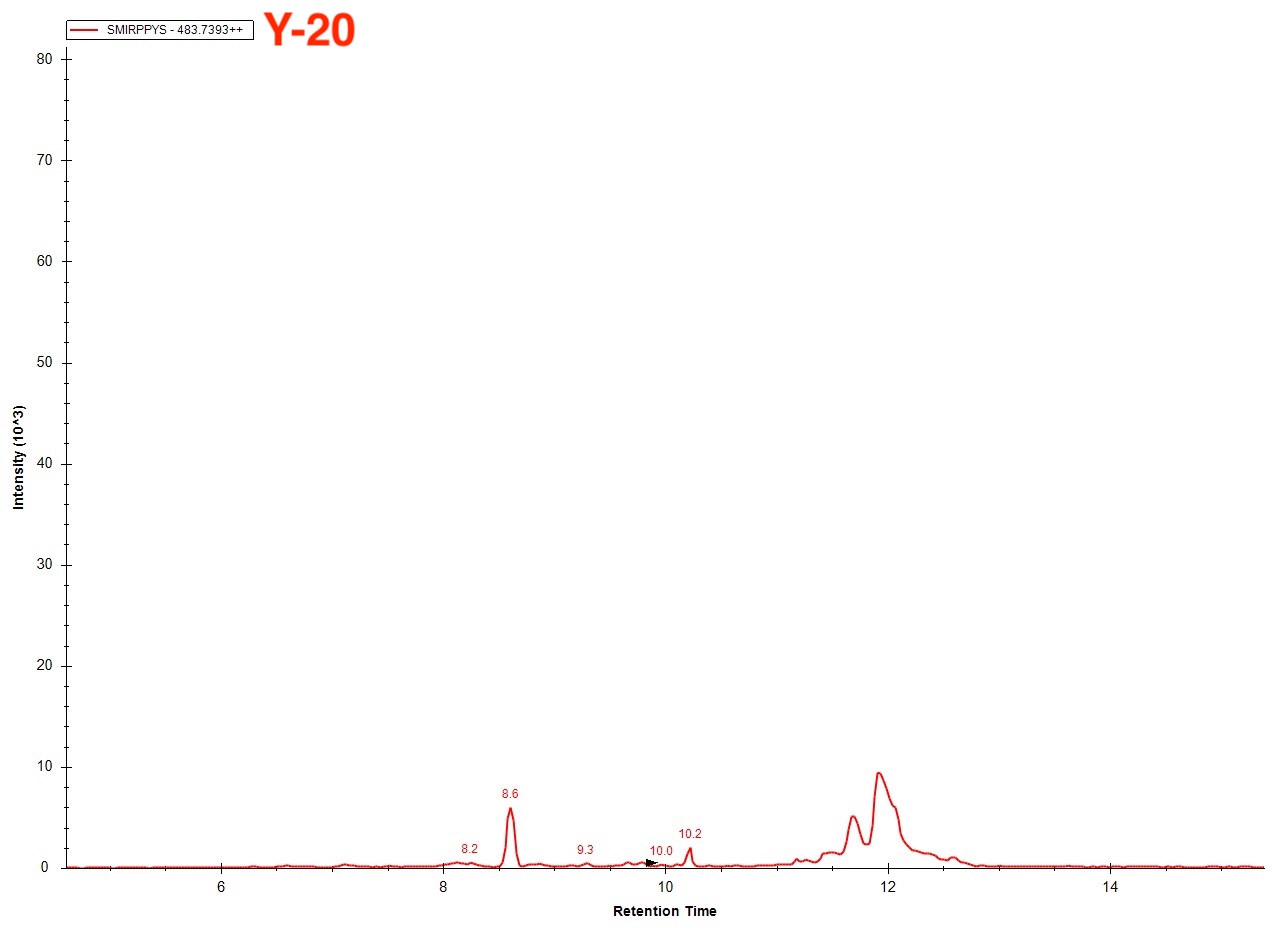


**Figure 13. Ion chromatogram of amino acid sequence SMIRPPYS - 483.7393++ (AMELY), individual 21.**
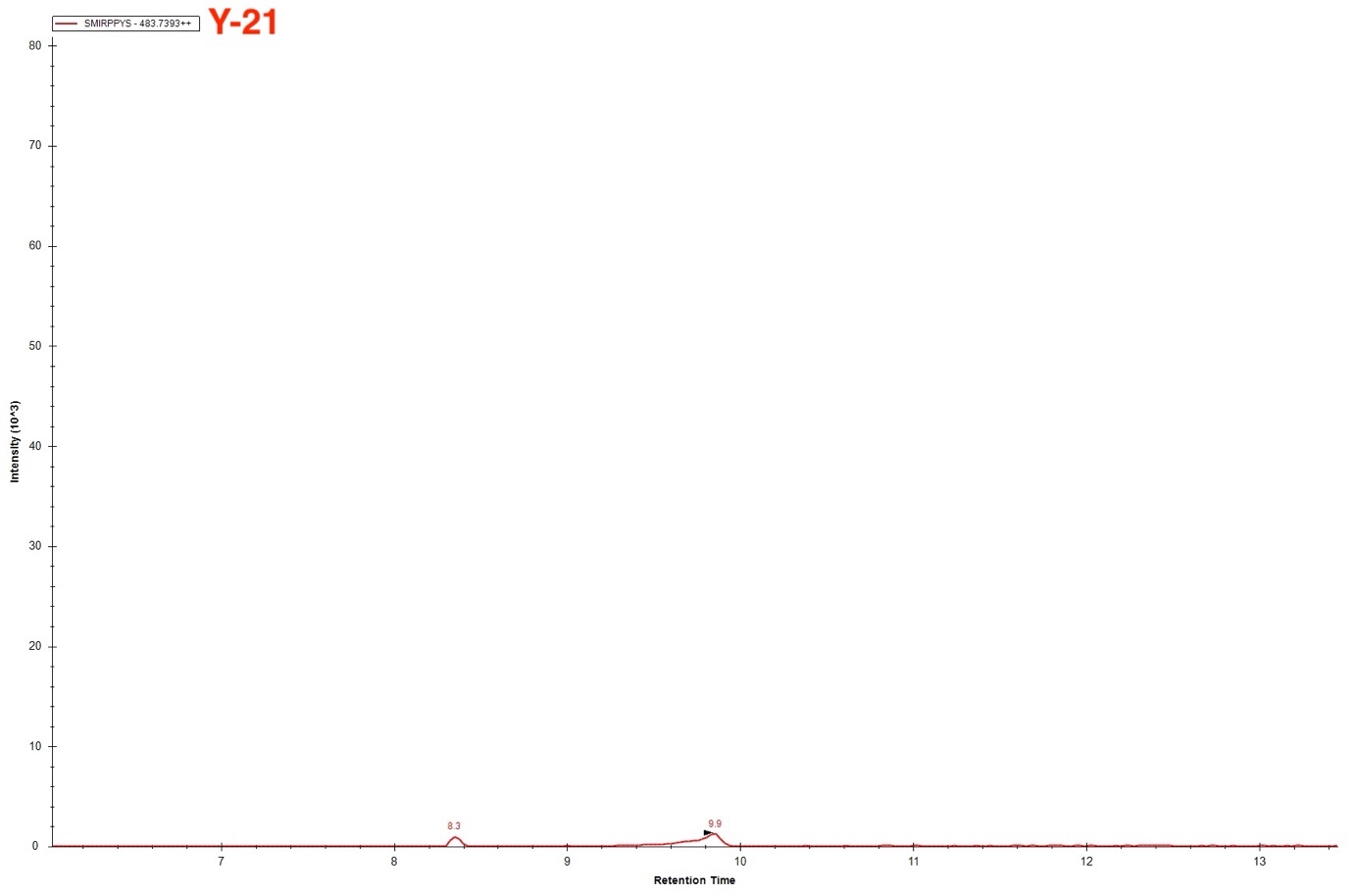


**Figure 14. Ion chromatogram of amino acid sequence SMIRPPYS - 483.7393++ (AMELY), individual 22.**
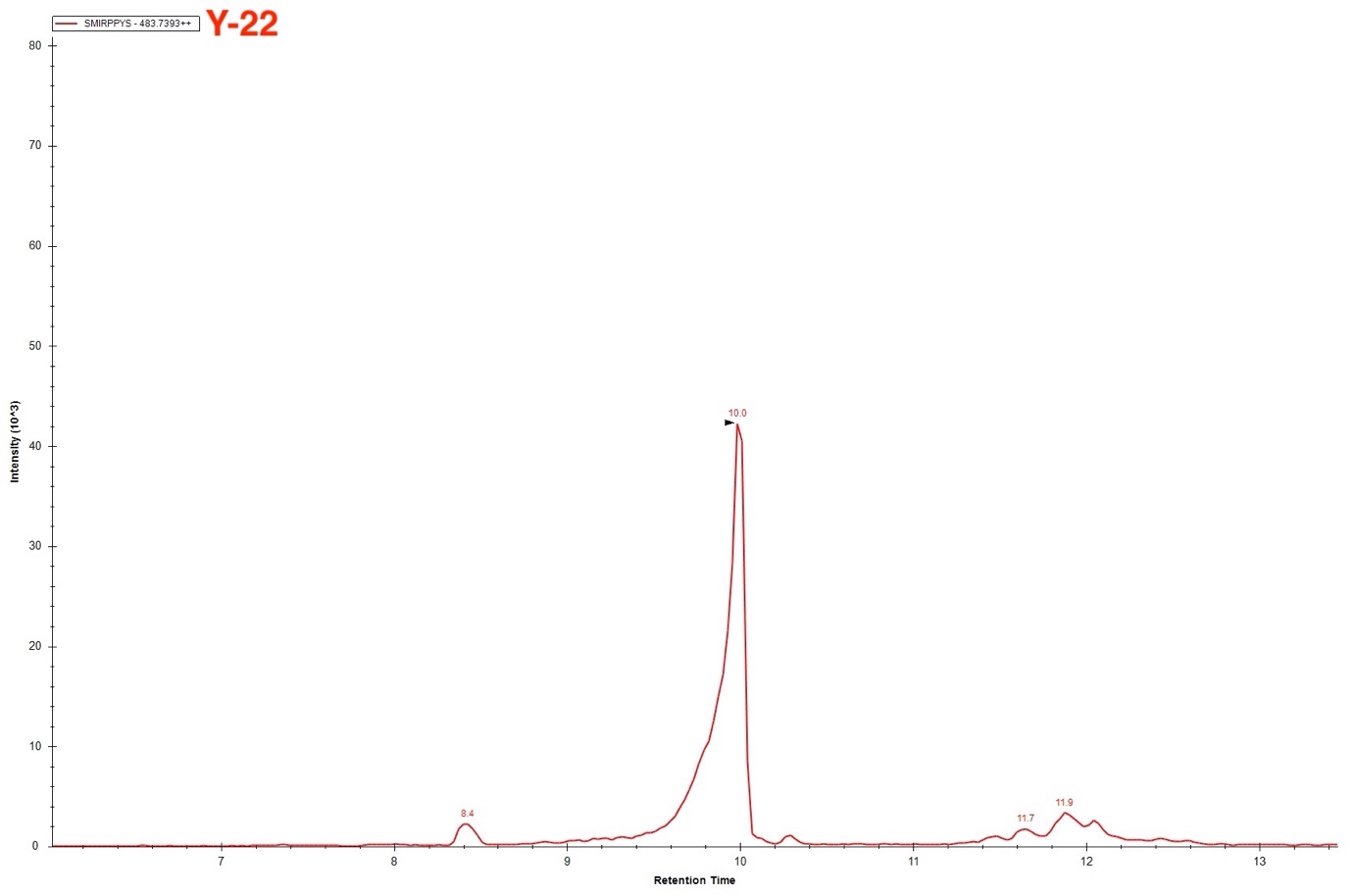


**Figure 15. Ion chromatogram of amino acid sequence SMIRPPYS - 483.7393++ (AMELY), individual 26.**
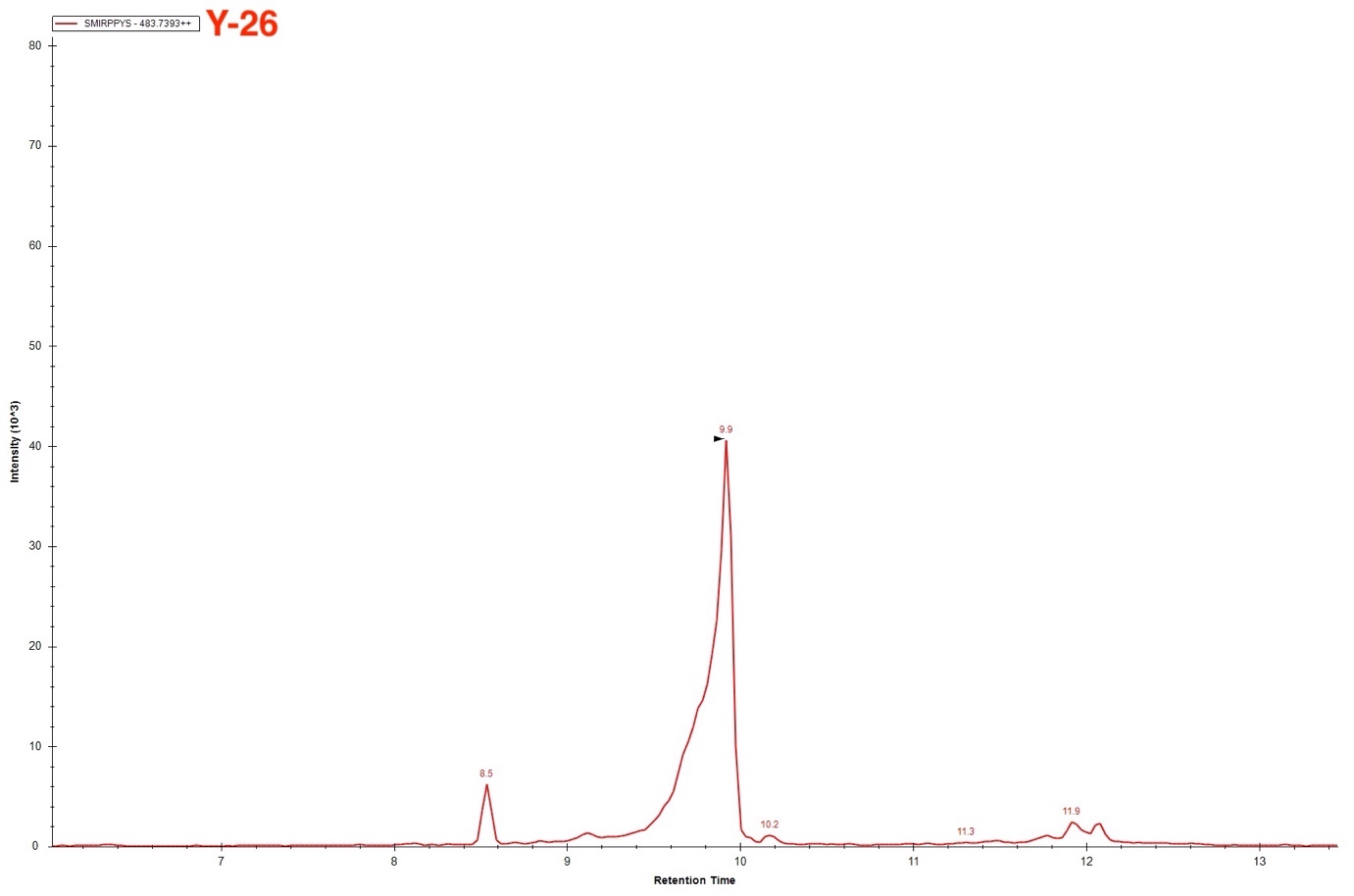


**Figure 16. Ion chromatogram of amino acid sequence SMIRPPYS - 483.7393++ (AMELY), individual 36.**
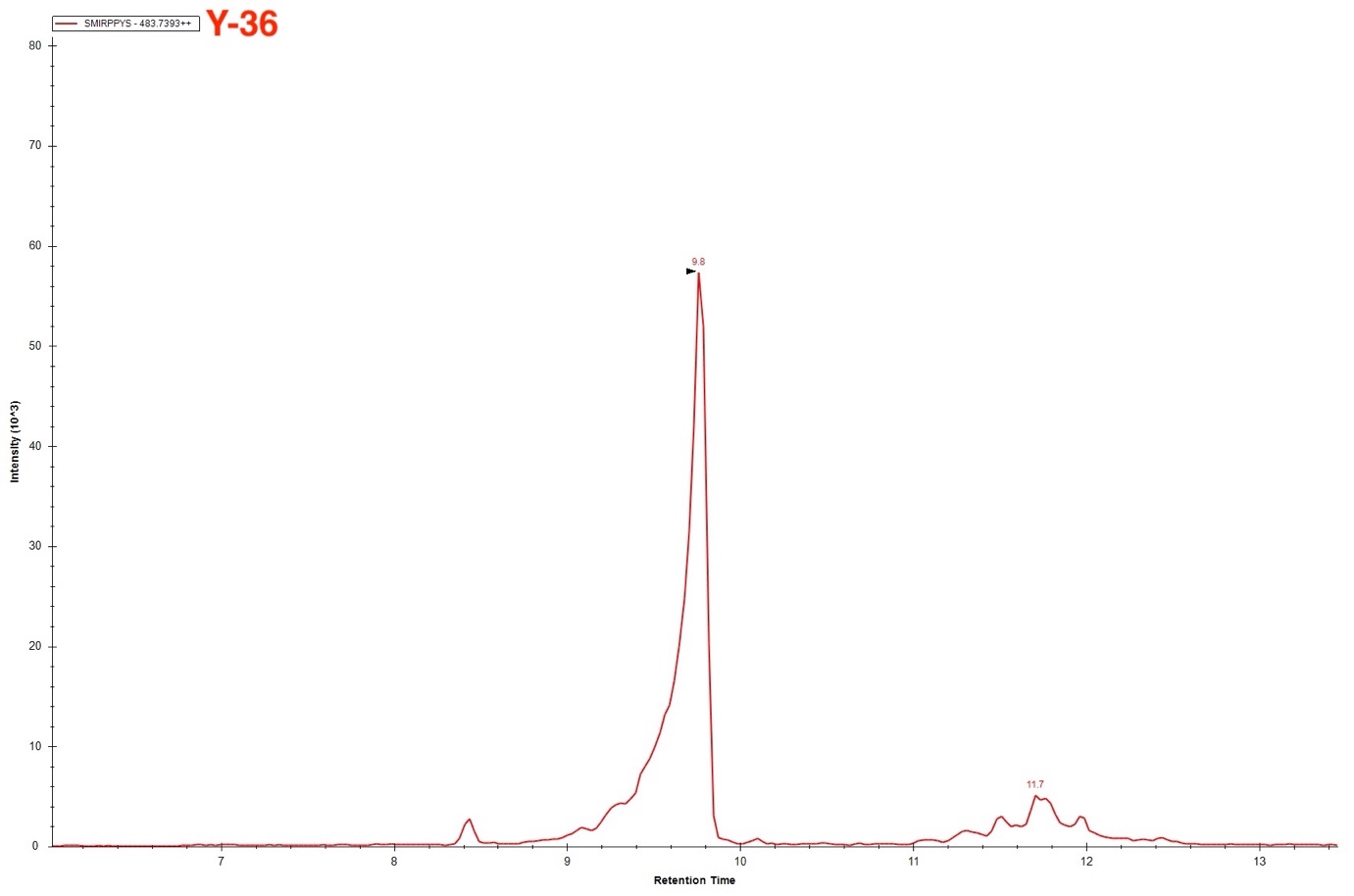


**Figure 17. Ion chromatogram of amino acid sequence SMIRPPYS - 483.7393++ (AMELY), individual 38.**
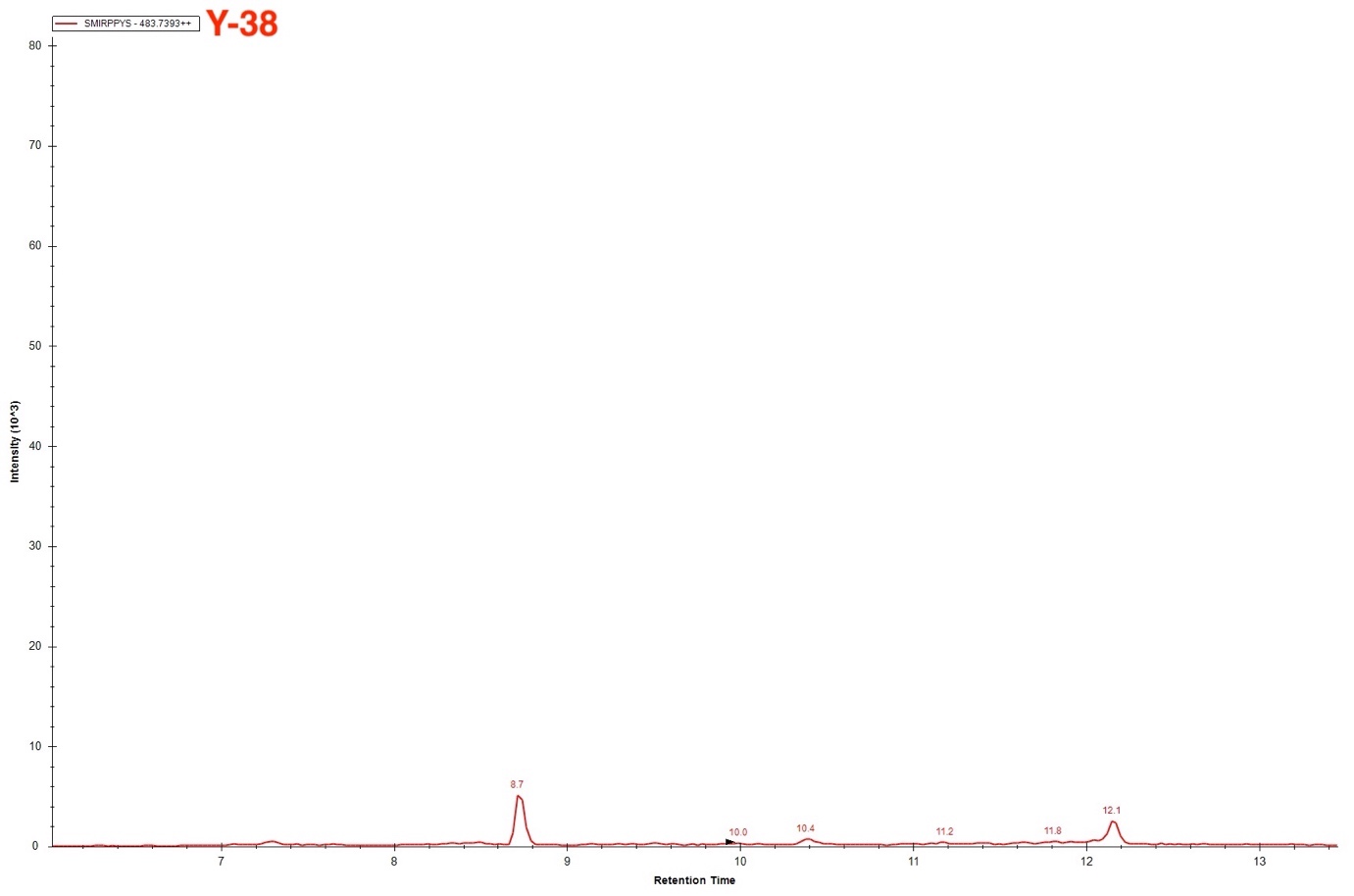


**Figure 18. Ion chromatogram of amino acid sequence SMIRPPYS - 483.7393++ (AMELY), individual 39.**
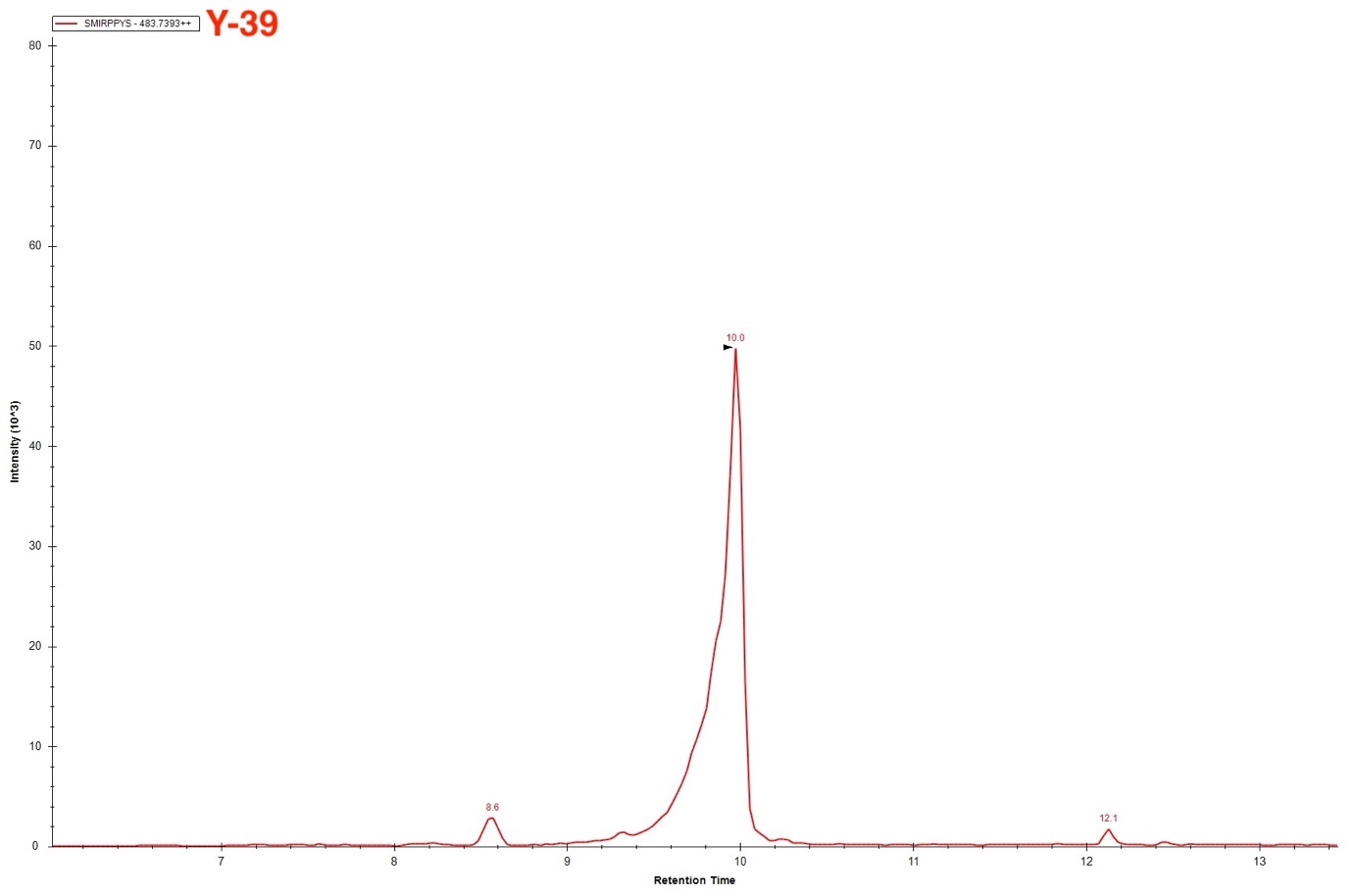


**Figure 19. Ion chromatogram of amino acid sequence SMIRPPYS - 483.7393++ (AMELY), individual 40.**
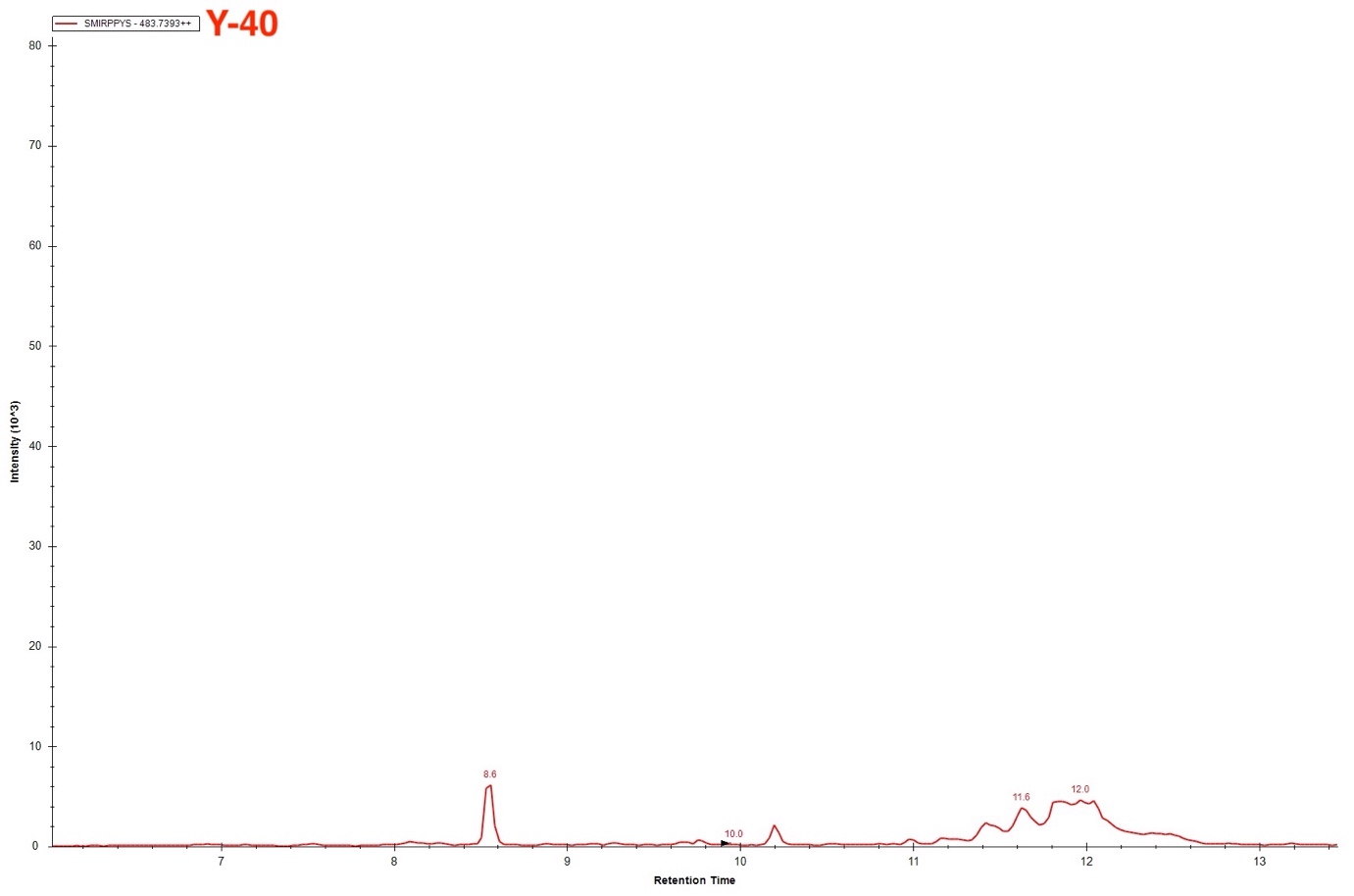


**Figure 20. Ion chromatogram of amino acid sequence SMIRPPYS - 483.7393++ (AMELY), individual 41.**
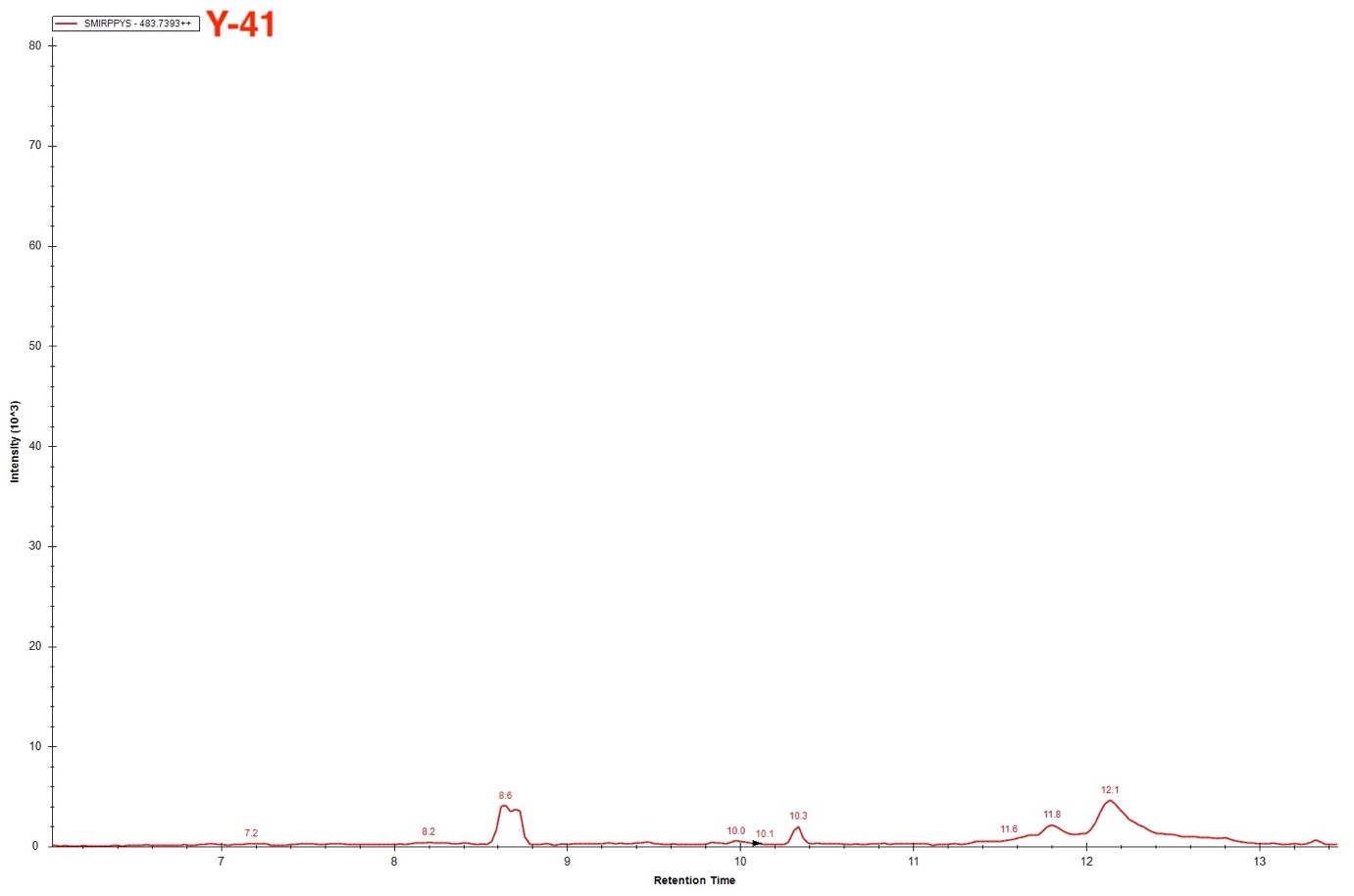


**Figure 21. Ion chromatogram of amino acid sequence SMIRPPYS - 483.7393++ (AMELY), individual 42.**
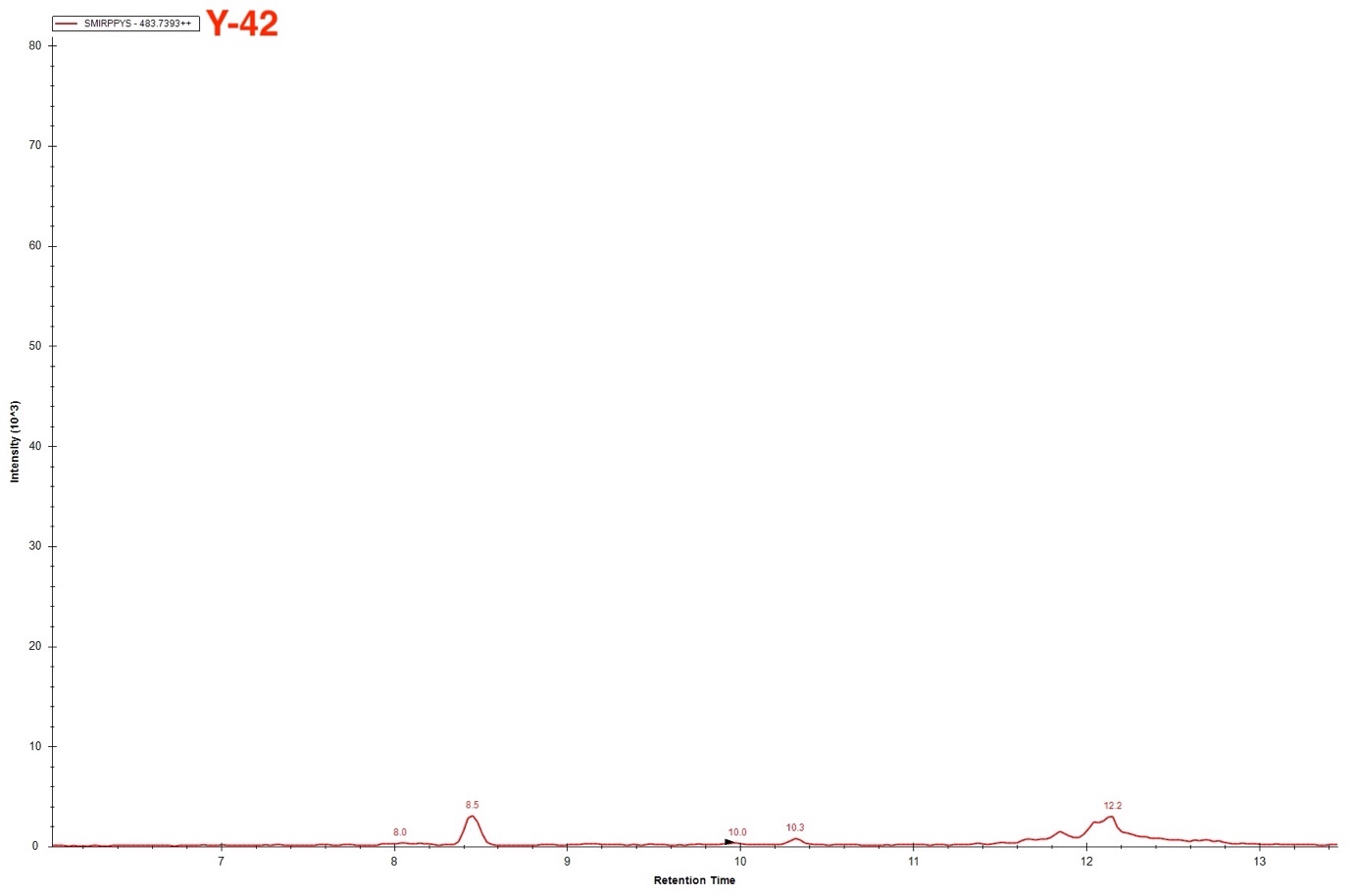


**Figure 22. Ion chromatogram of amino acid sequence SMIRPPYS - 483.7393++ (AMELY), individual 44.**
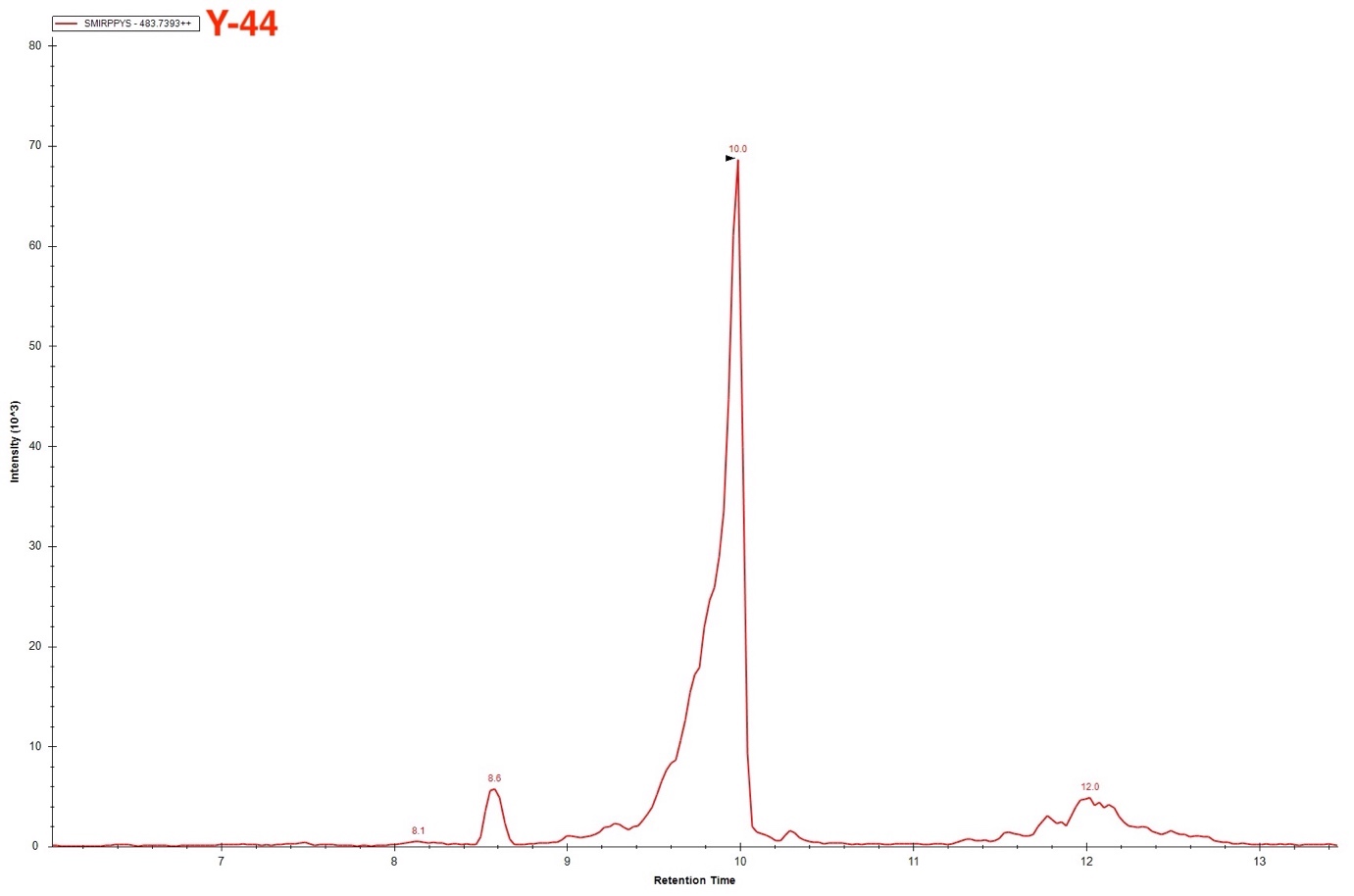


**Figure 23. Ion chromatogram of amino acid sequence SMIRPPYS - 483.7393++ (AMELY), individual 46.**
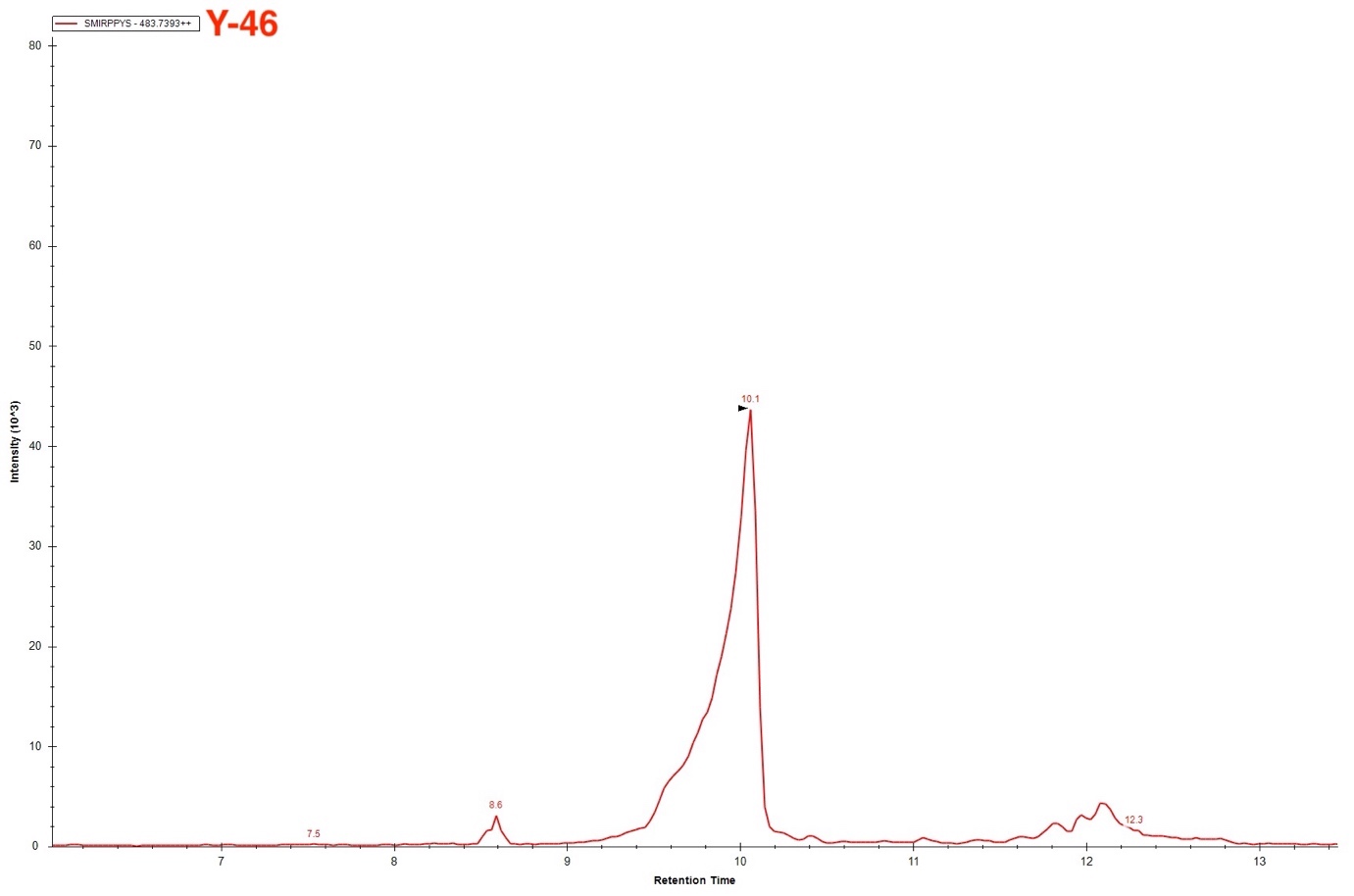


**Figure 24. Ion chromatogram of amino acid sequence SMIRPPYS - 483.7393++ (AMELY), individual 54.5**
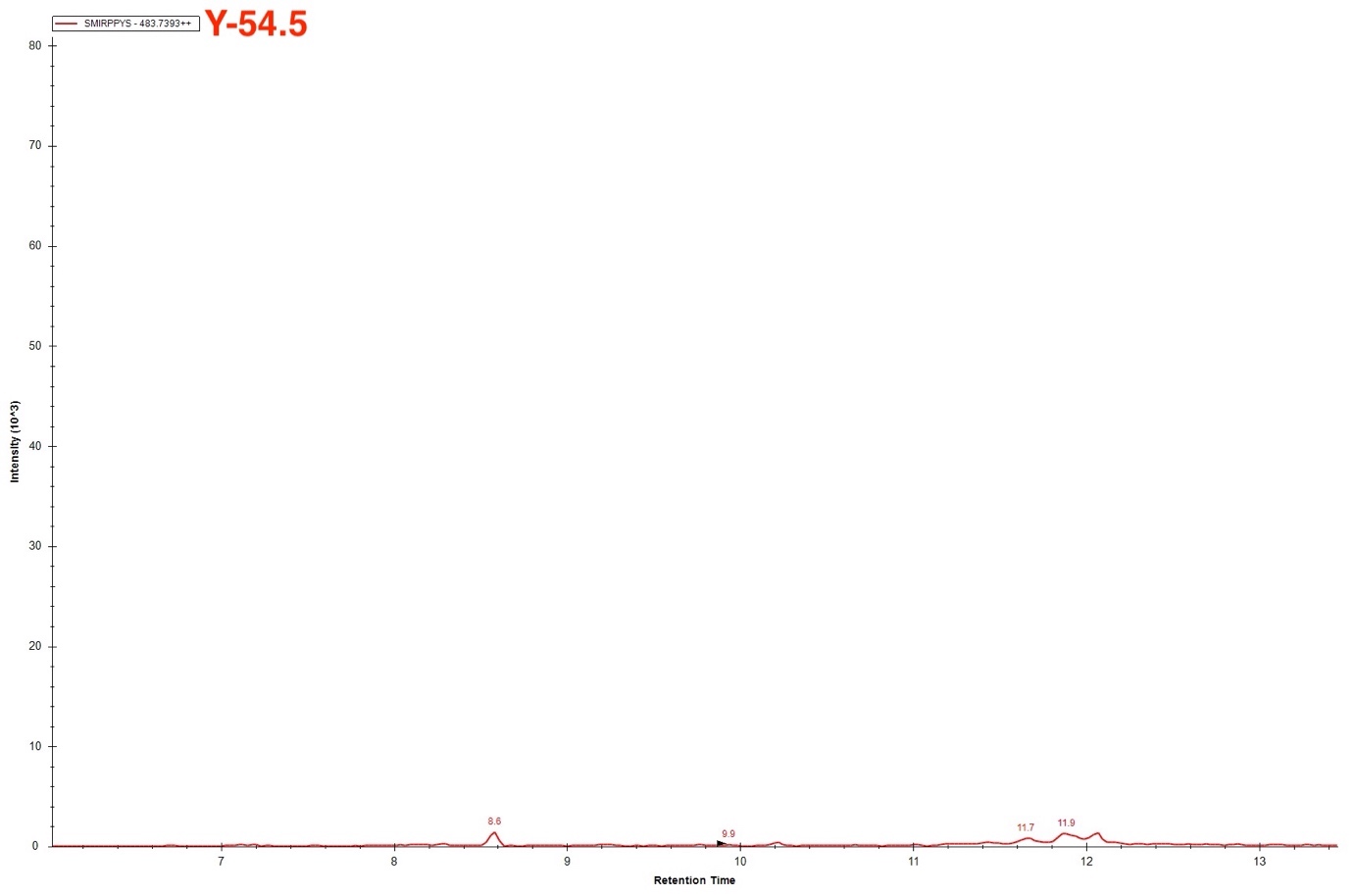


**Figure 25. Ion chromatogram of amino acid sequence SMIRPPYS - 483.7393++ (AMELY), individual SLR.**
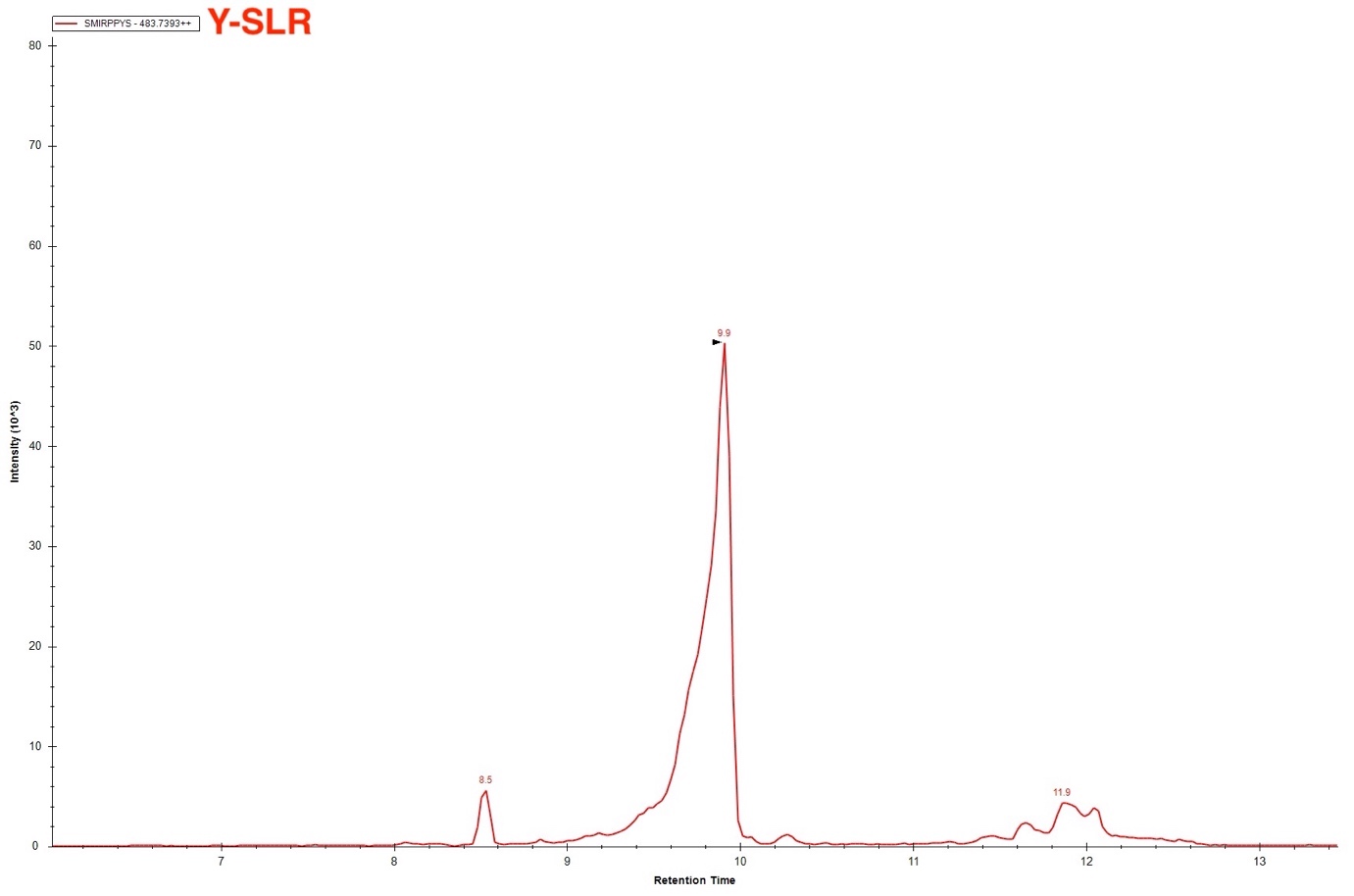


**Figure 26. Ion chromatogram of amino acid sequence SMIRPPYS - 483.7393++ (AMELY), individual SSJ.**


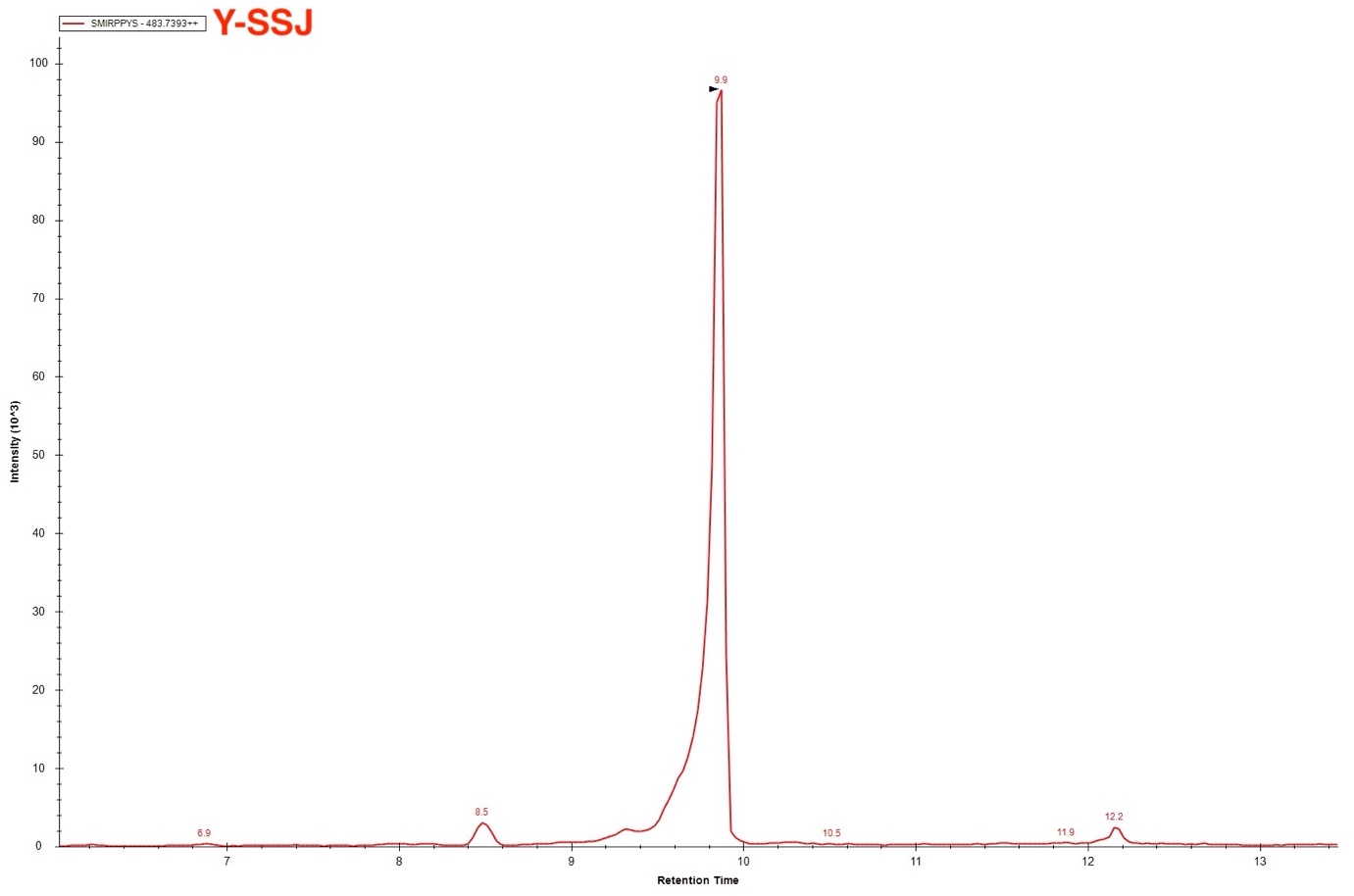


**Figure 27. Ion chromatogram of amino acid sequence SMIRPPY - 440.2233++ (AMELY), individual 1.**


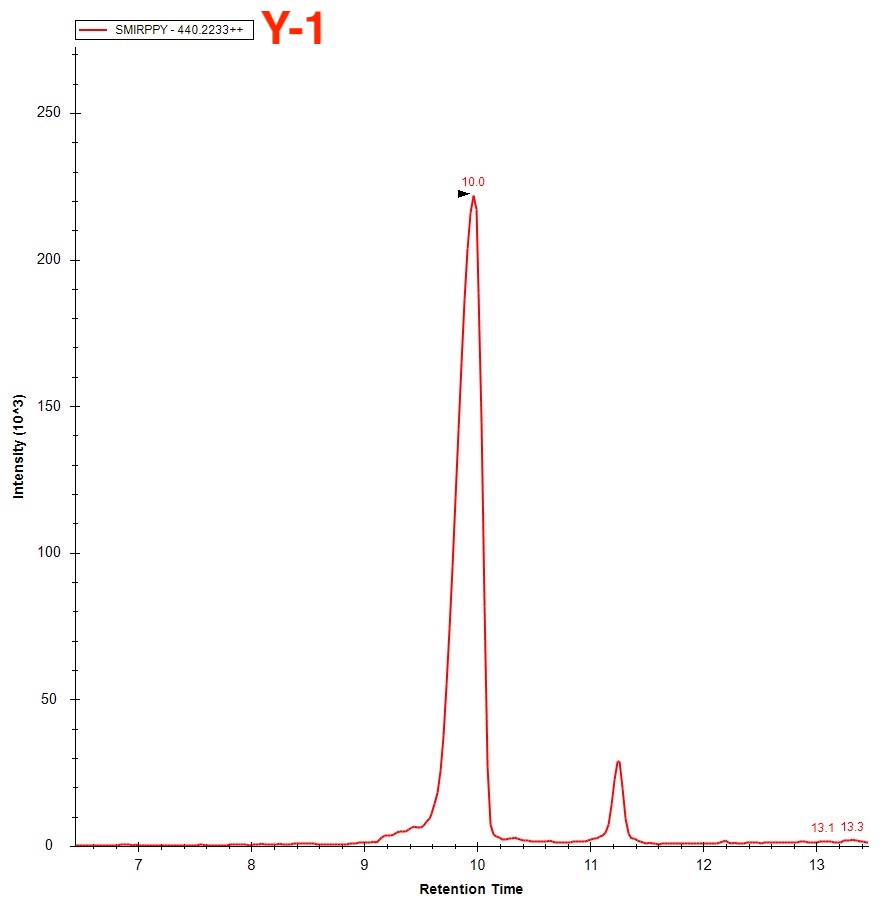


**Figure 28. Ion chromatogram of amino acid sequence SMIRPPY - 440.2233++ (AMELY), individual 2.**
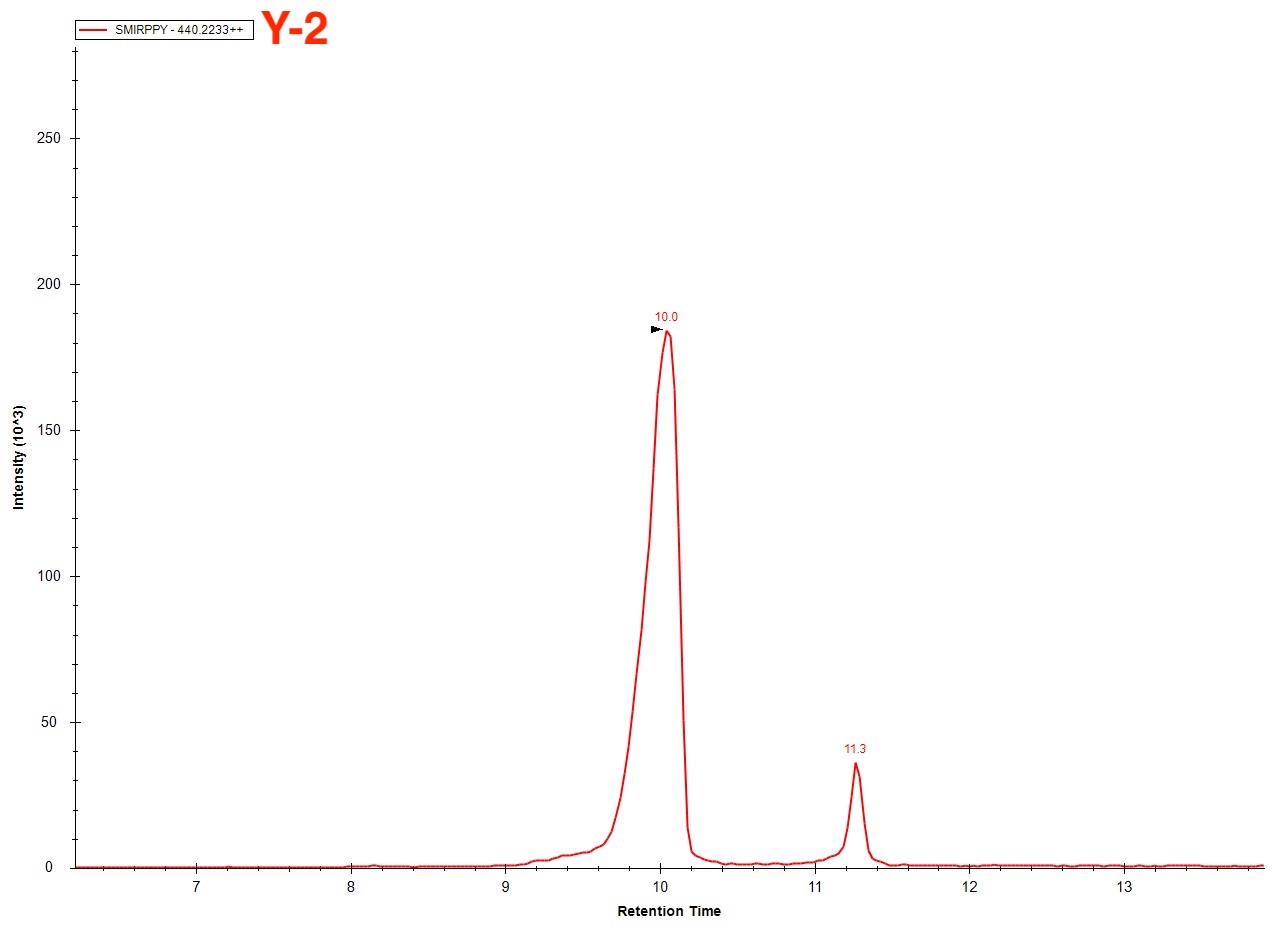


**Figure 29. Ion chromatogram of amino acid sequence SMIRPPY - 440.2233++ (AMELY), individual 4.**


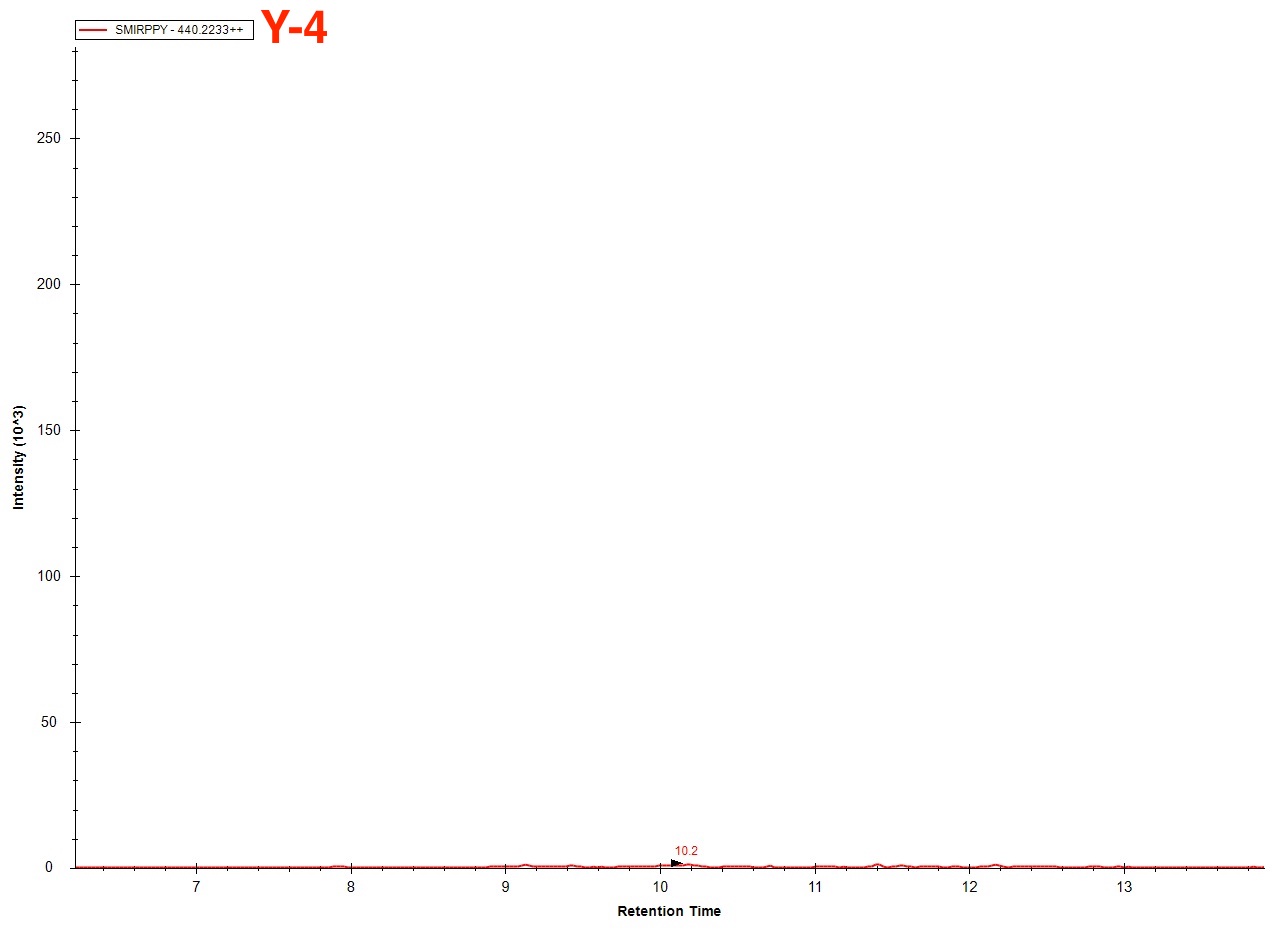


**Figure 30. Ion chromatogram of amino acid sequence SMIRPPY - 440.2233++ (AMELY), individual 6.**
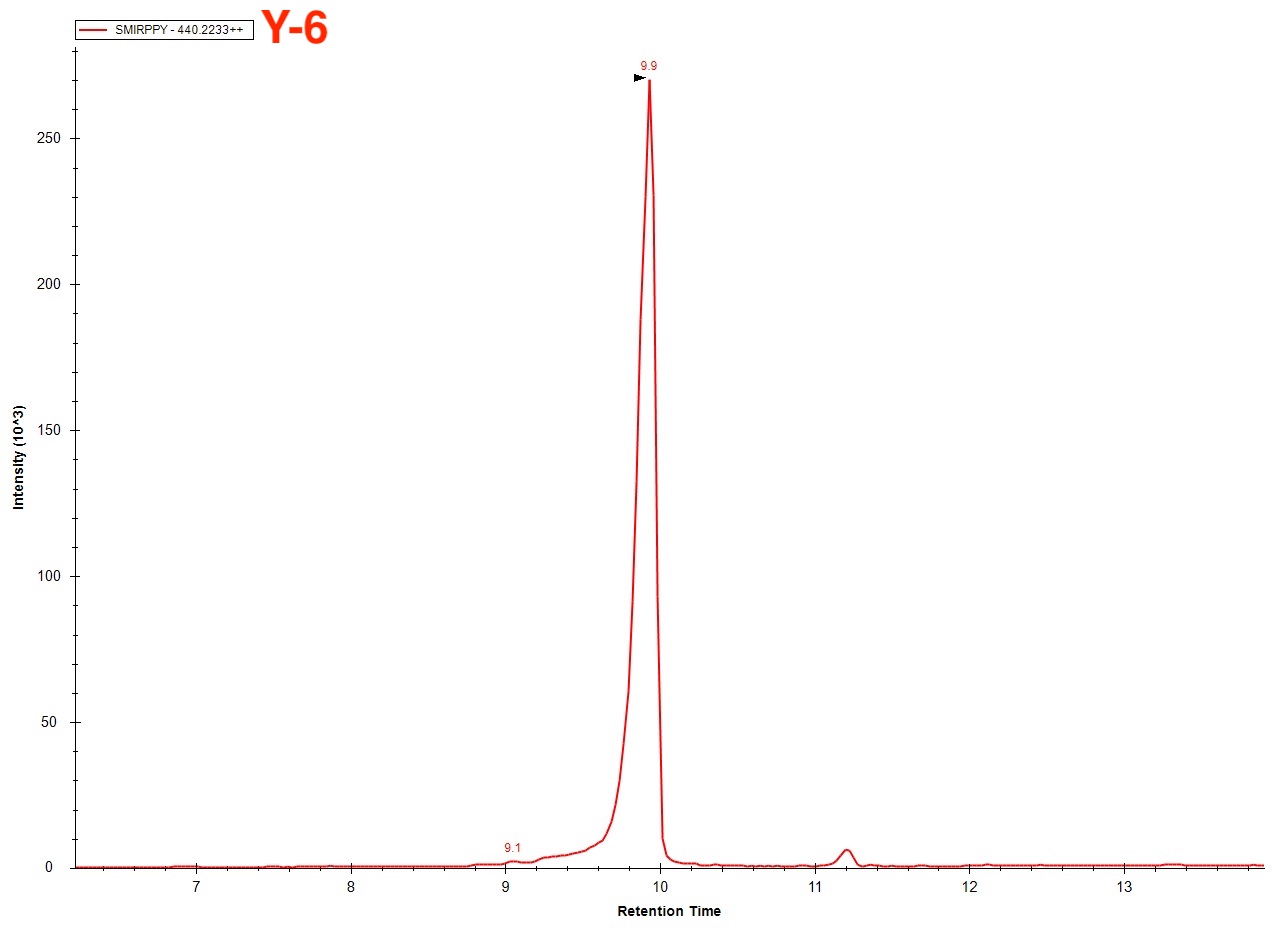


**Figure 31. Ion chromatogram of amino acid sequence SMIRPPY - 440.2233++ (AMELY), individual 7.**


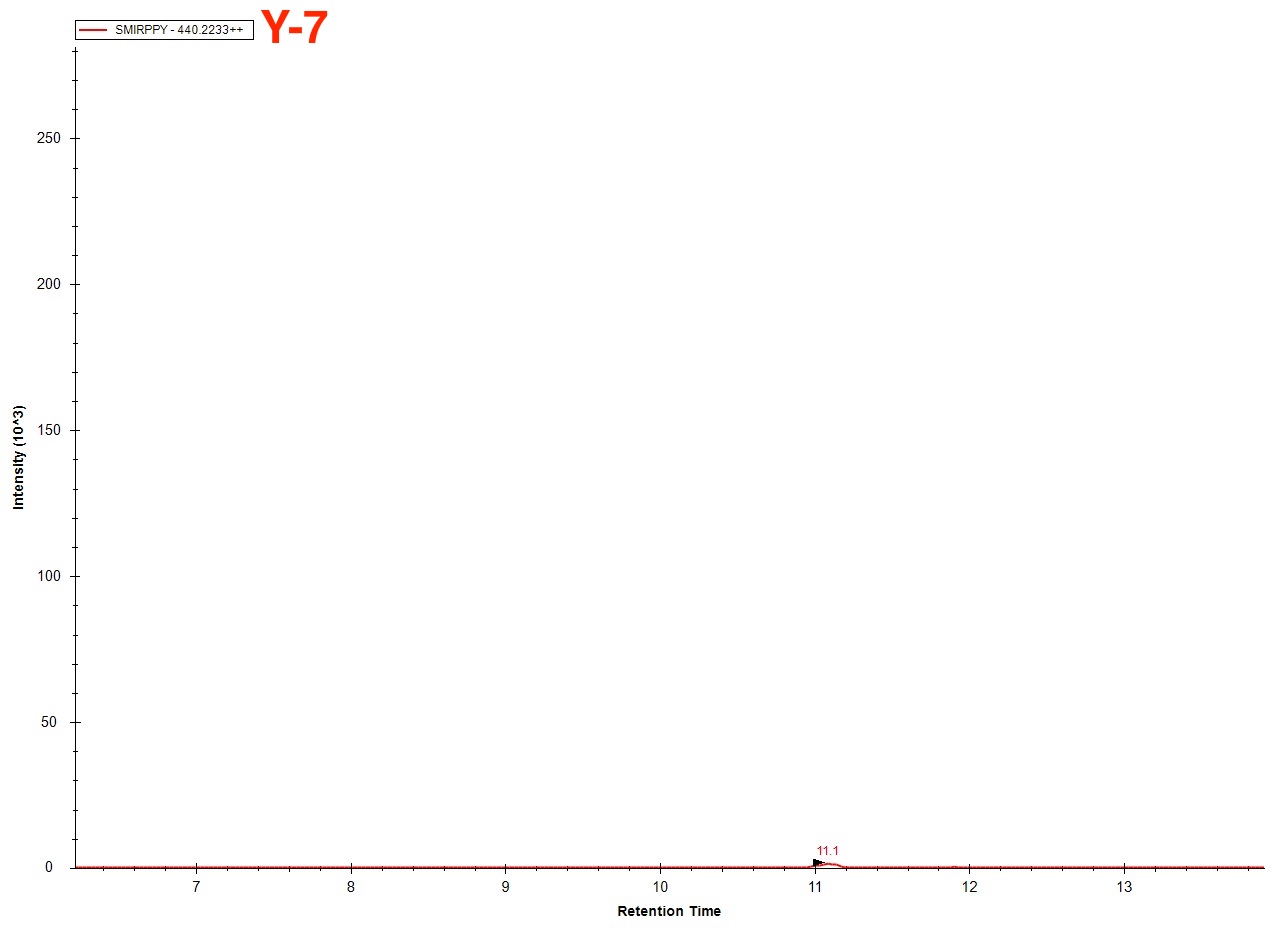


**Figure 32. Ion chromatogram of amino acid sequence SMIRPPY - 440.2233++ (AMELY), individual 9.**


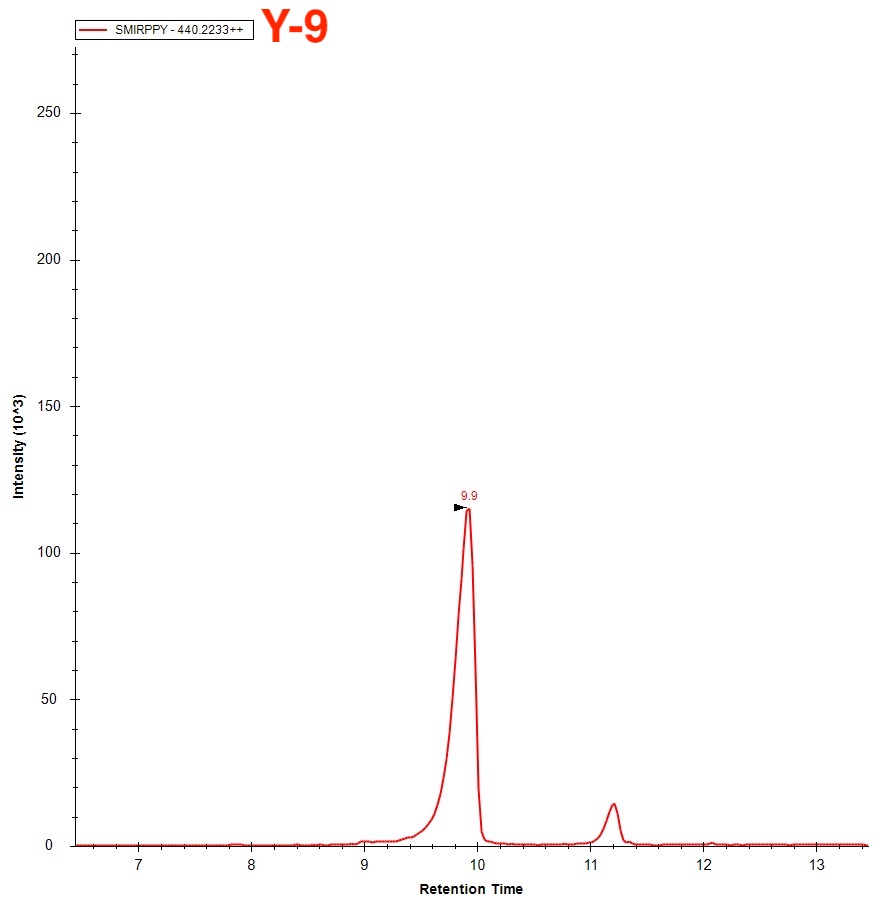


**Figure 33. Ion chromatogram of amino acid sequence SMIRPPY - 440.2233++ (AMELY), individual 13.**


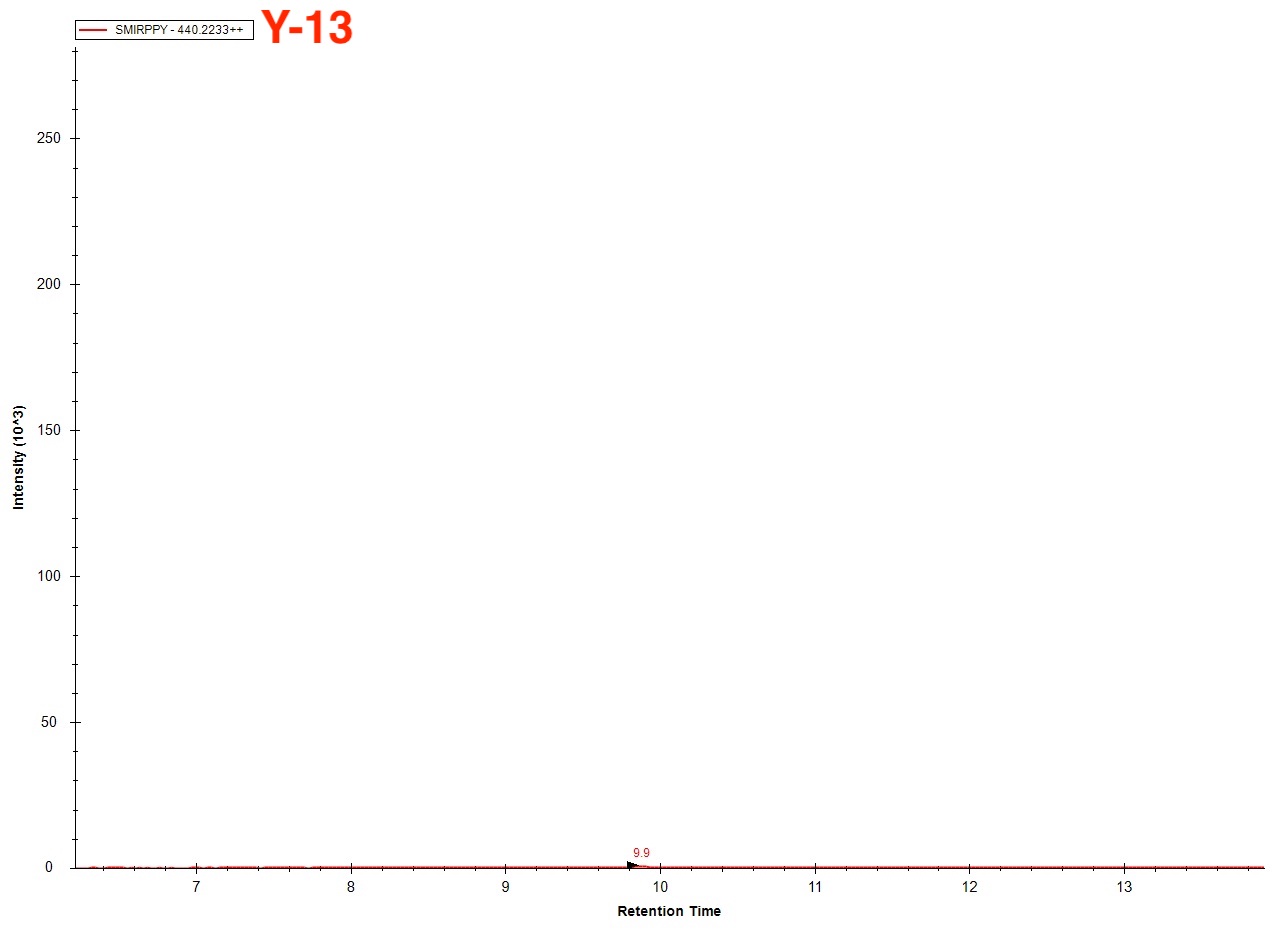


**Figure 34. Ion chromatogram of amino acid sequence SMIRPPY - 440.2233++ (AMELY), individual 14.**


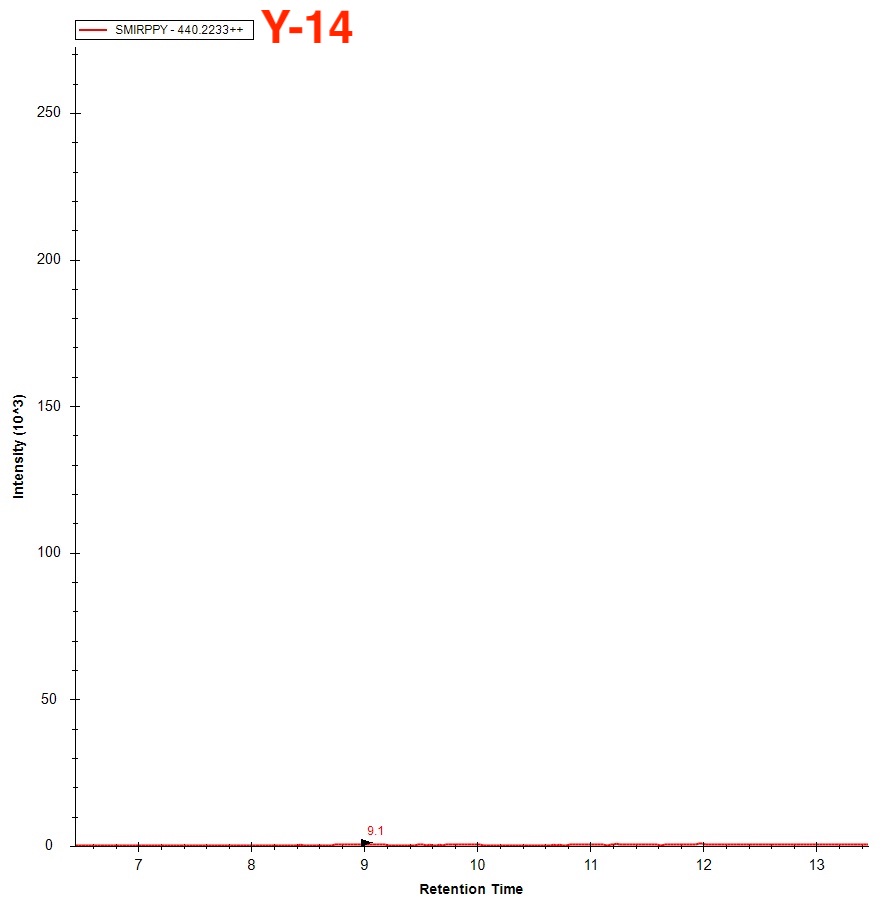


**Figure 35. Ion chromatogram of amino acid sequence SMIRPPY - 440.2233++ (AMELY), individual 15.**


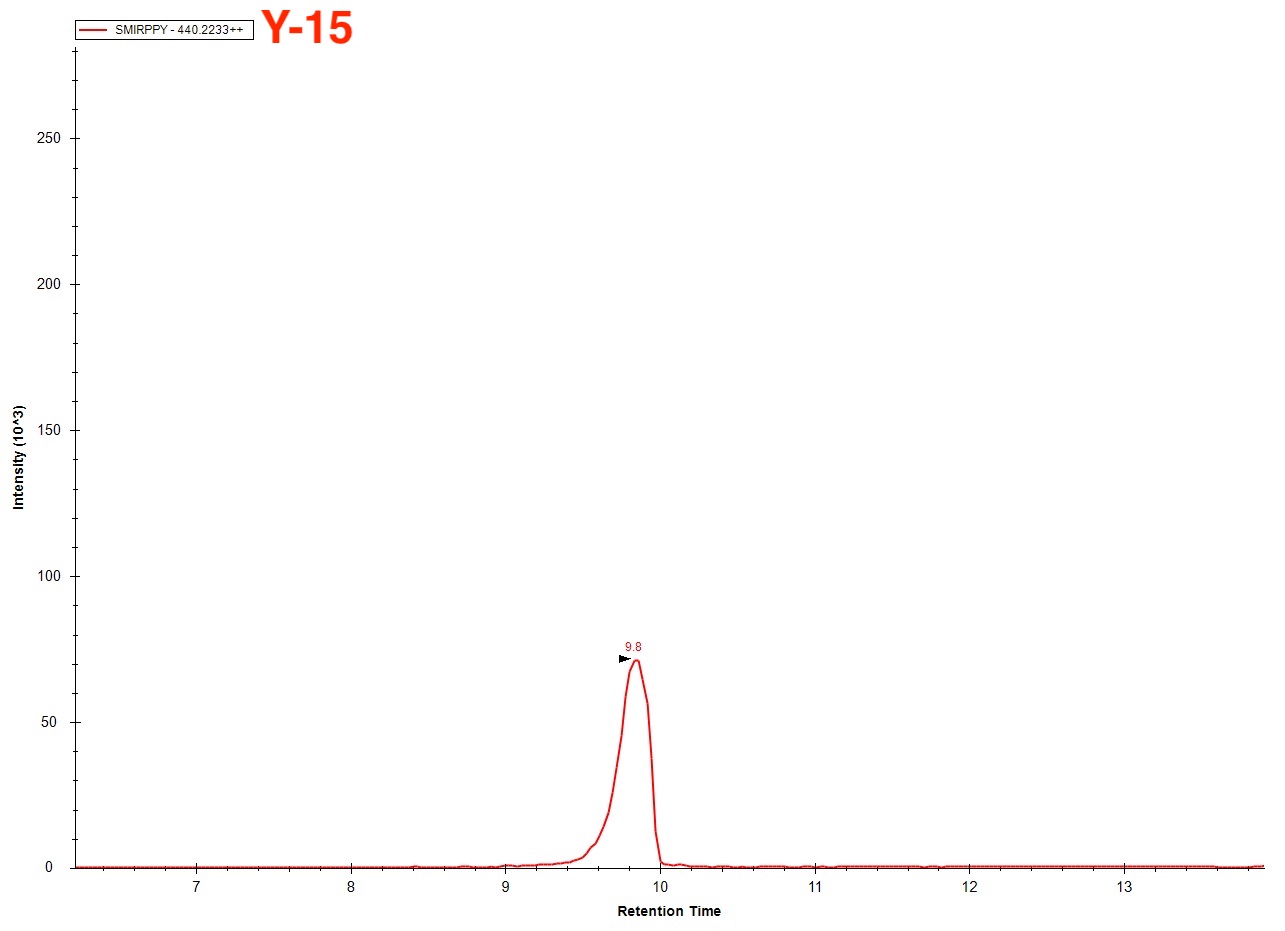


**Figure 36. Ion chromatogram of amino acid sequence SMIRPPY - 440.2233++ (AMELY), individual 16.**
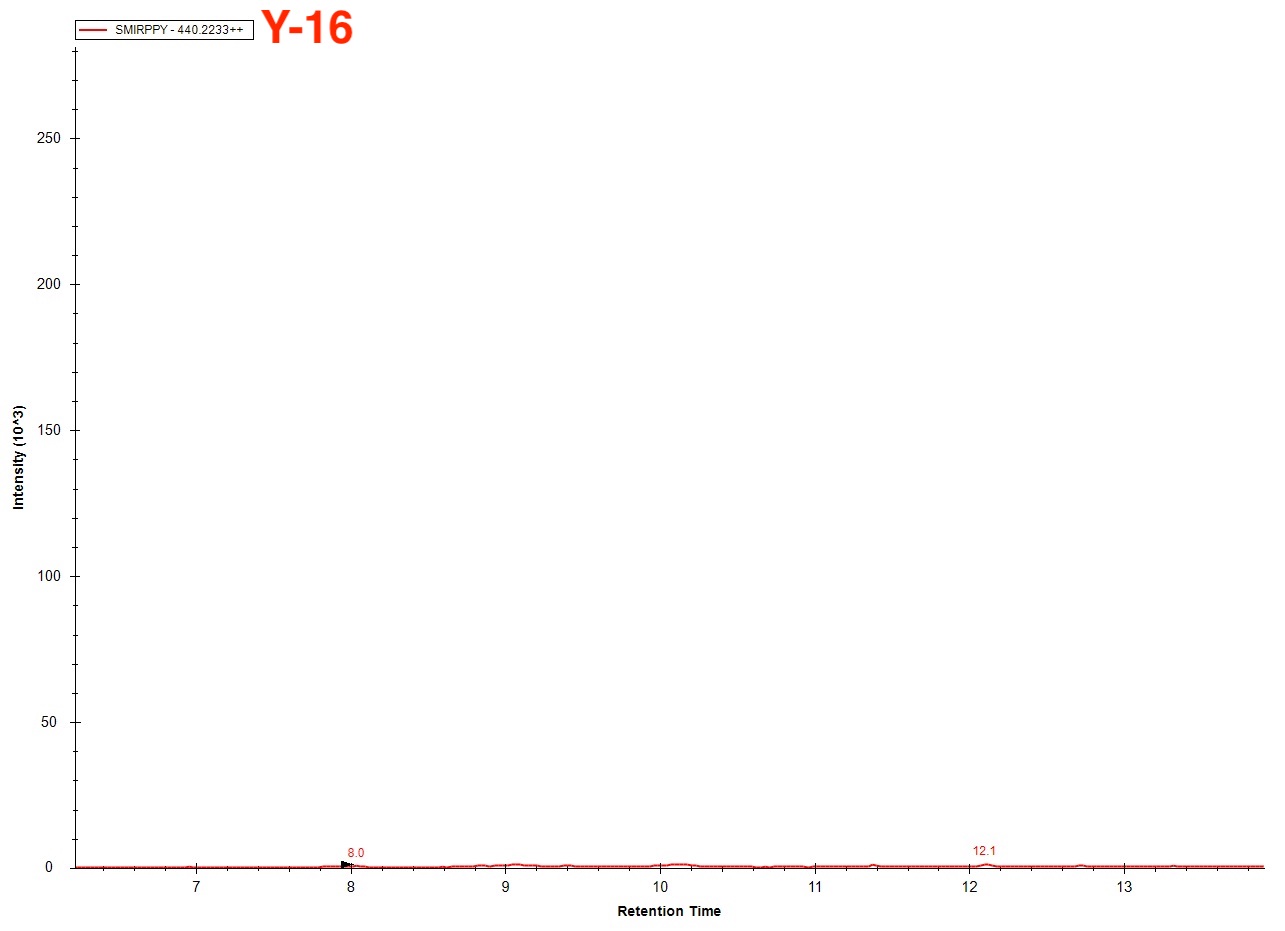


**Figure 37. Ion chromatogram of amino acid sequence SMIRPPY - 440.2233++ (AMELY), individual 17.**


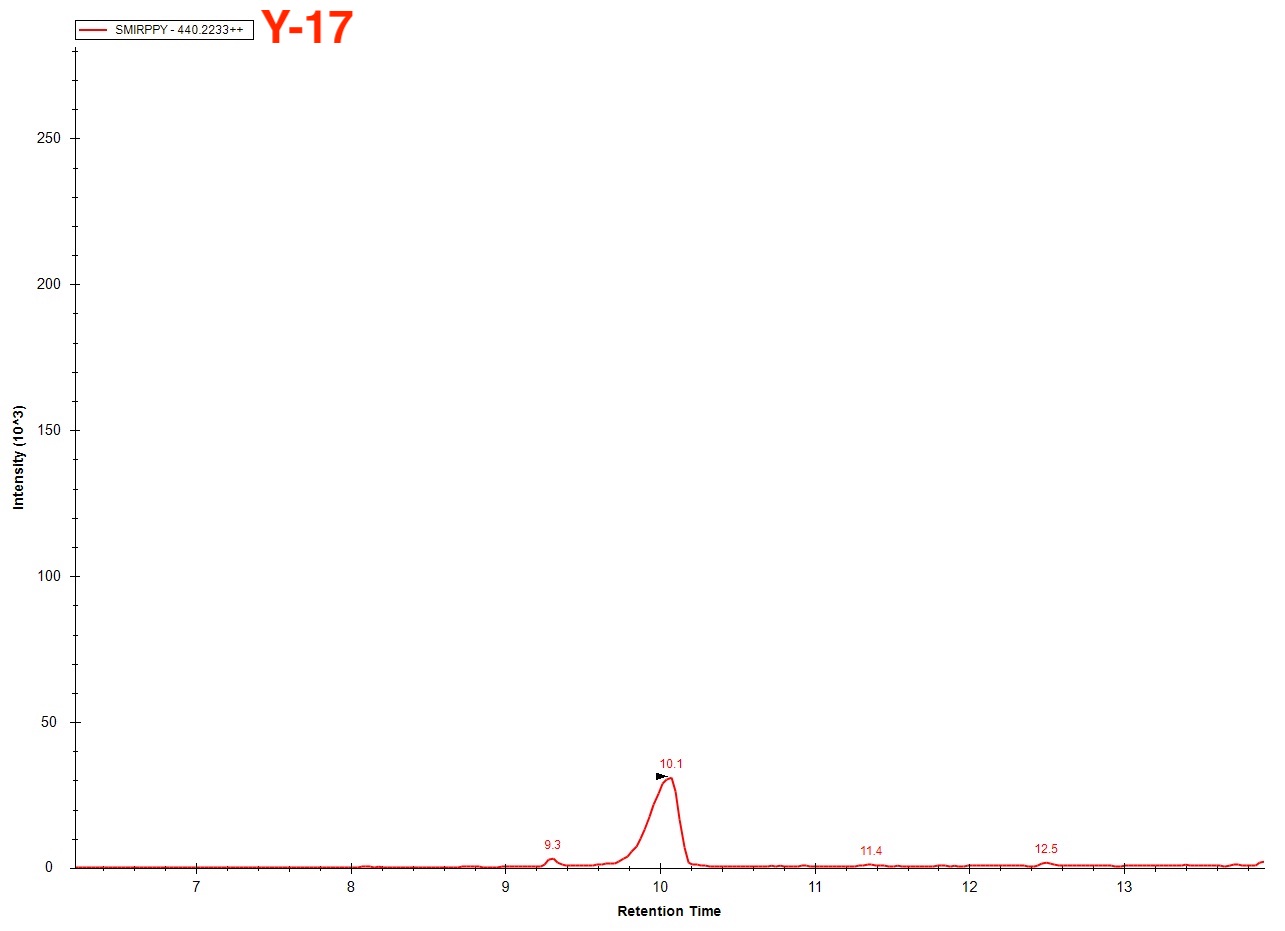


**Figure 38. Ion chromatogram of amino acid sequence SMIRPPY - 440.2233++ (AMELY), individual 20.**
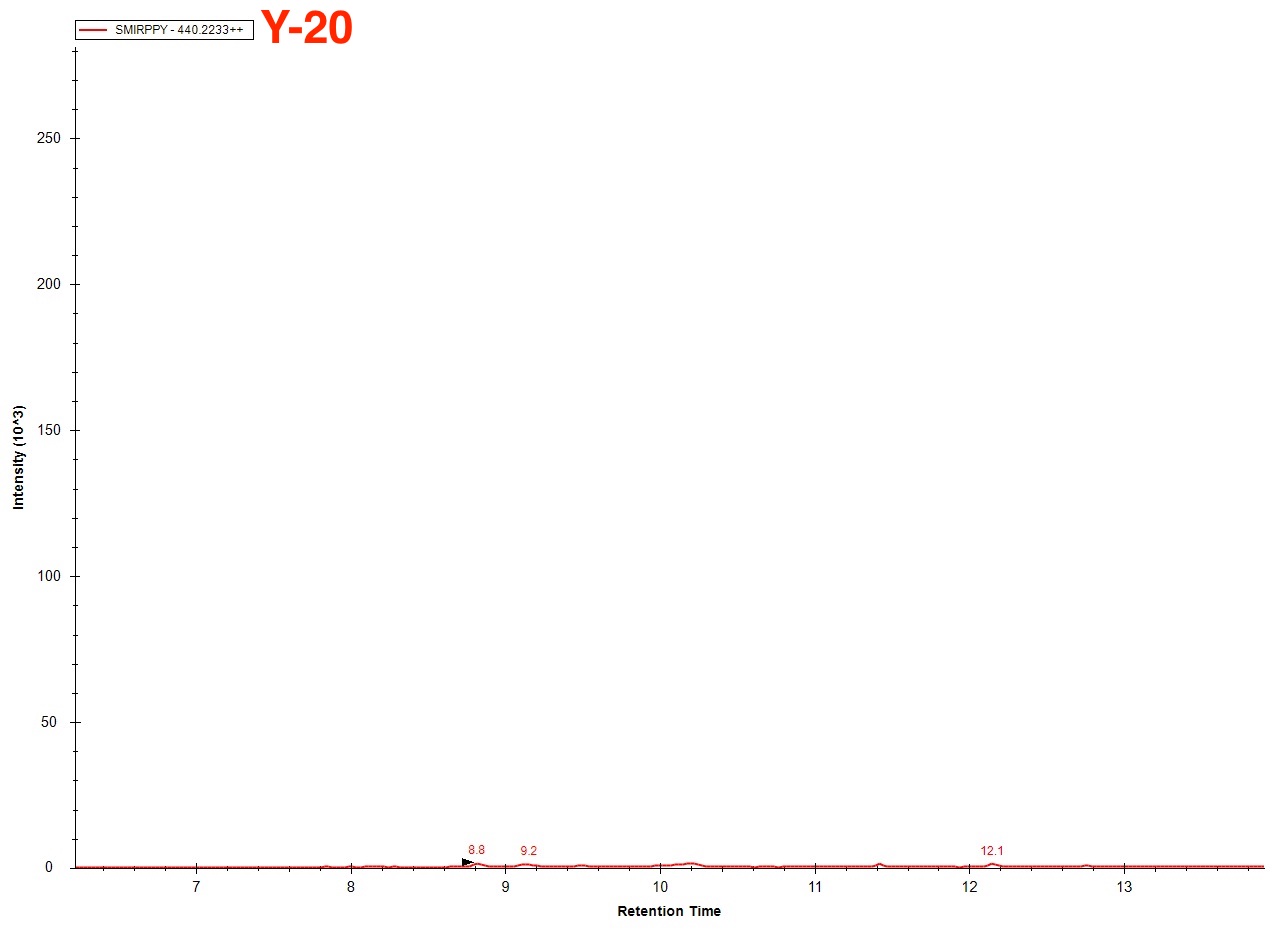


**Figure 39. Ion chromatogram of amino acid sequence SMIRPPY - 440.2233++ (AMELY), individual 21.**


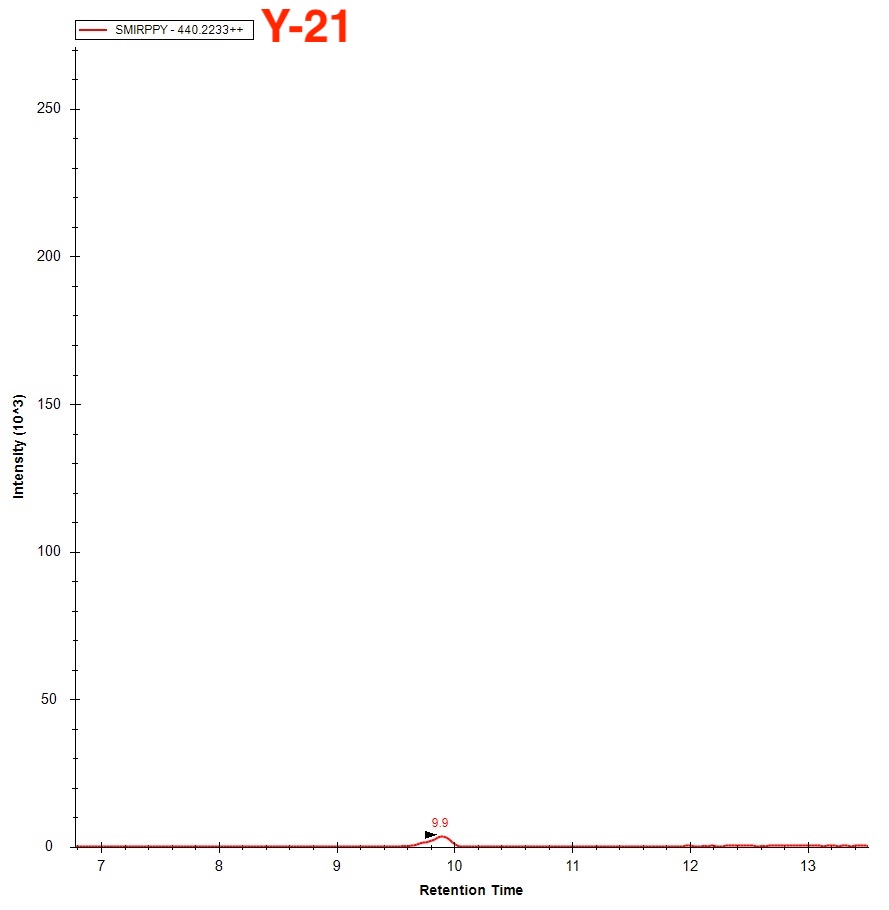


**Figure 40. Ion chromatogram of amino acid sequence SMIRPPY - 440.2233++ (AMELY), individual 22.**


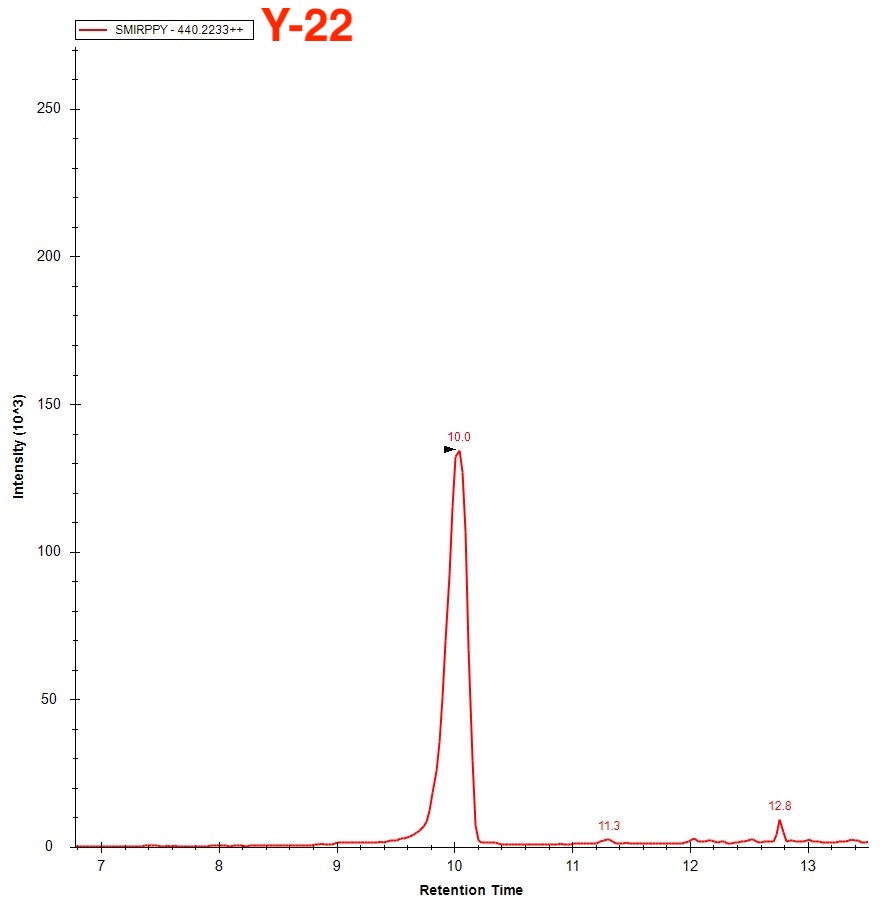


**Figure 41. Ion chromatogram of amino acid sequence SMIRPPY - 440.2233++ (AMELY), individual 26.**


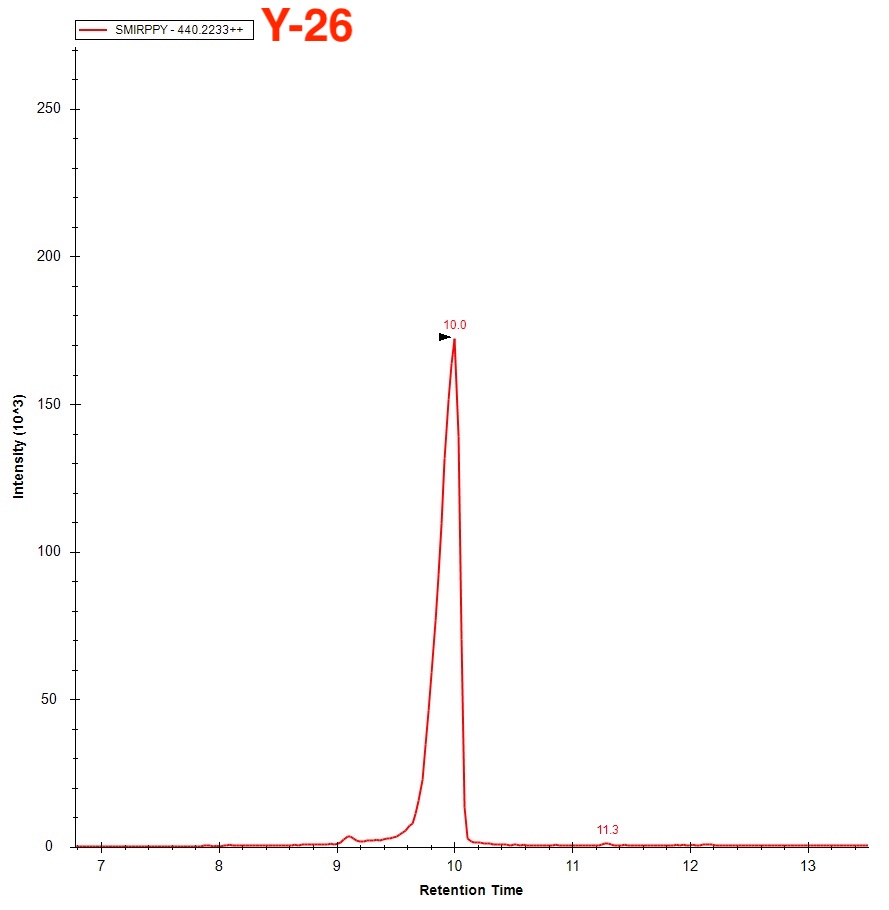


**Figure 42. Ion chromatogram of amino acid sequence SMIRPPY - 440.2233++ (AMELY), individual 36.**


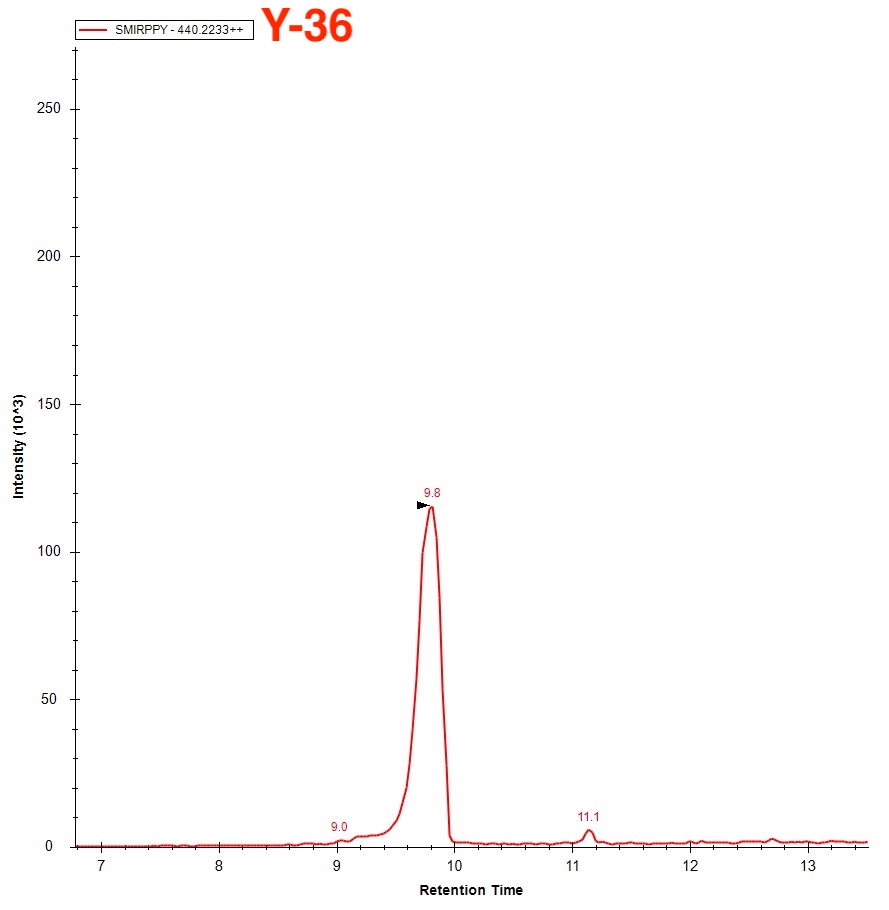


**Figure 43. Ion chromatogram of amino acid sequence SMIRPPY - 440.2233++ (AMELY), individual 38.**


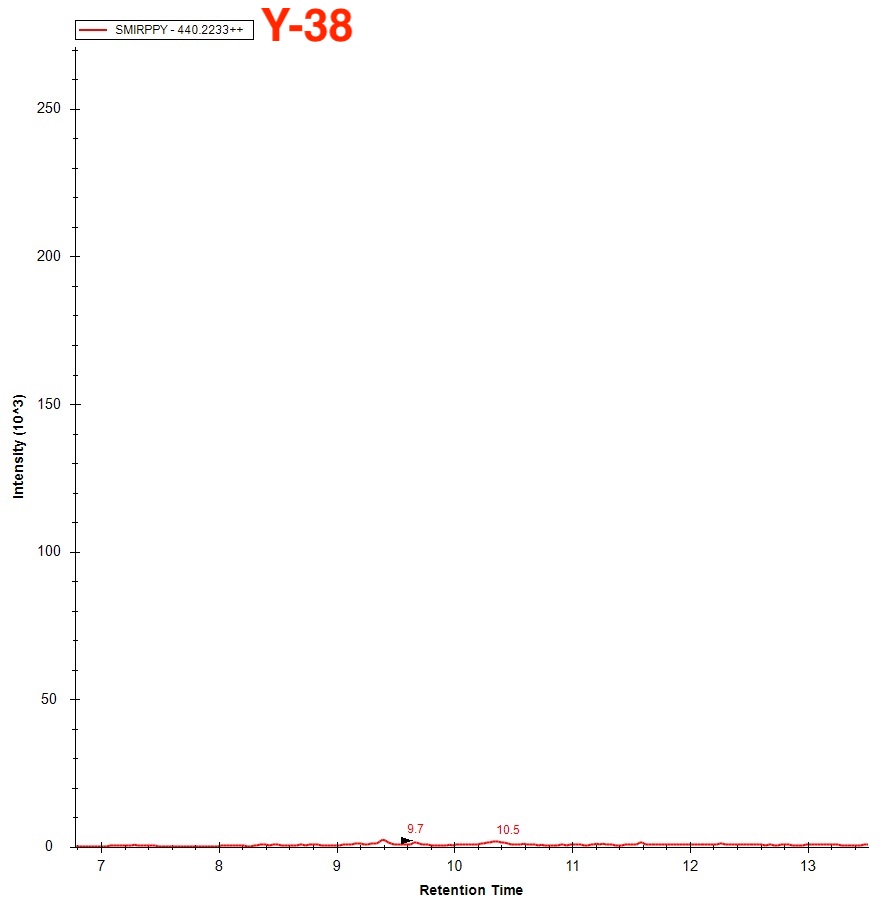


**Figure 44. Ion chromatogram of amino acid sequence SMIRPPY - 440.2233++ (AMELY), individual 39.**


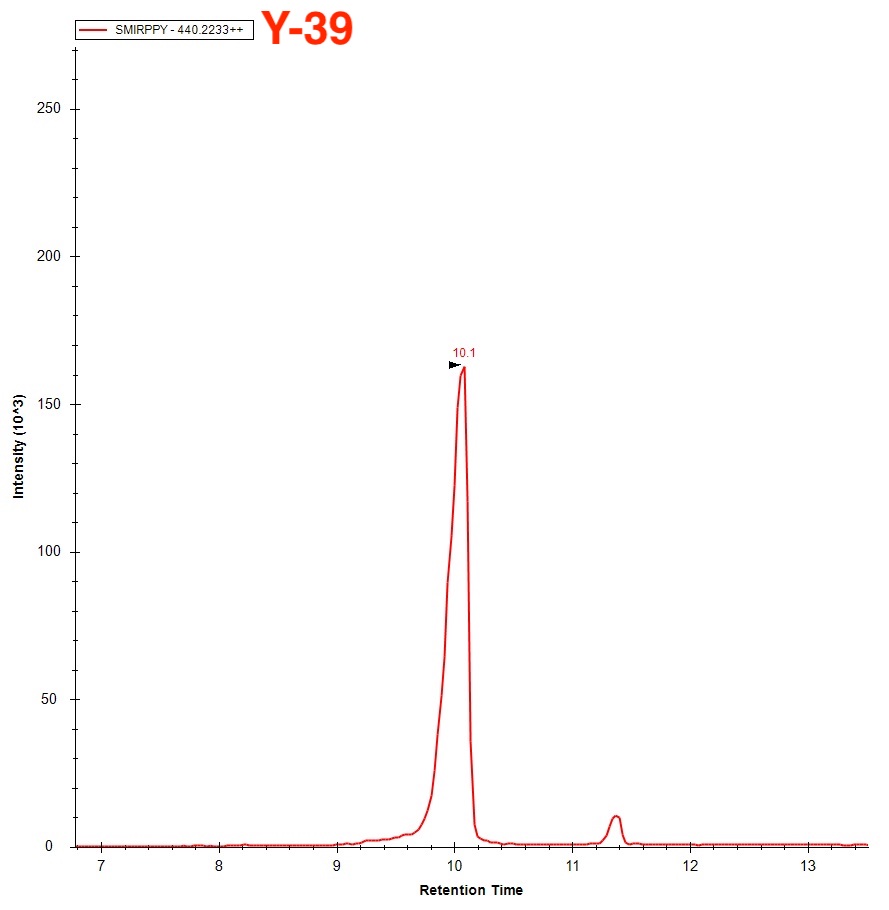


**Figure 45. Ion chromatogram of amino acid sequence SMIRPPY - 440.2233++ (AMELY), individual 40.**


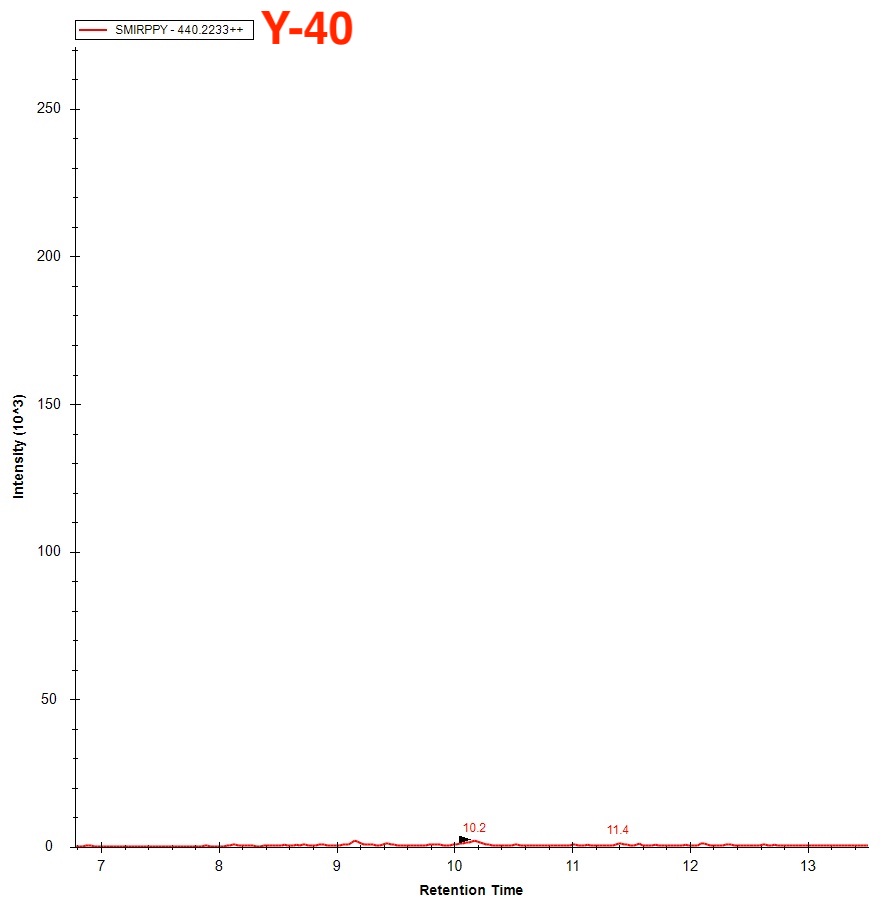


**Figure 46. Ion chromatogram of amino acid sequence SMIRPPY - 440.2233++ (AMELY), individual 41.**


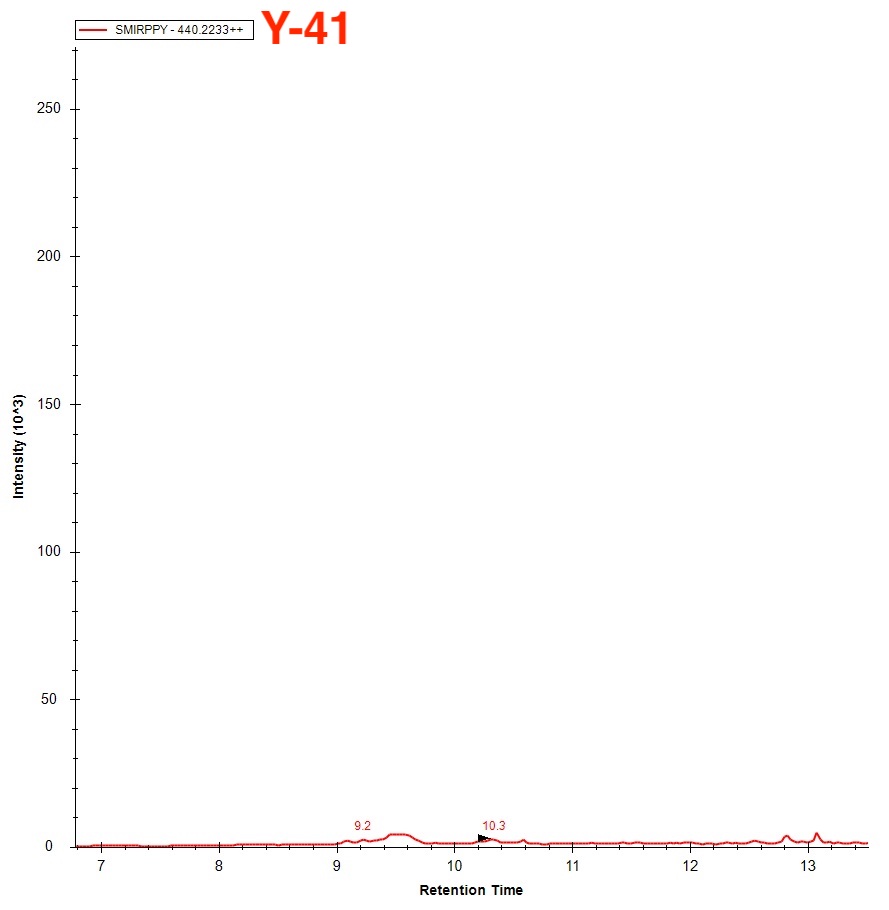


**Figure 47. Ion chromatogram of amino acid sequence SMIRPPY - 440.2233++ (AMELY), individual 42.**


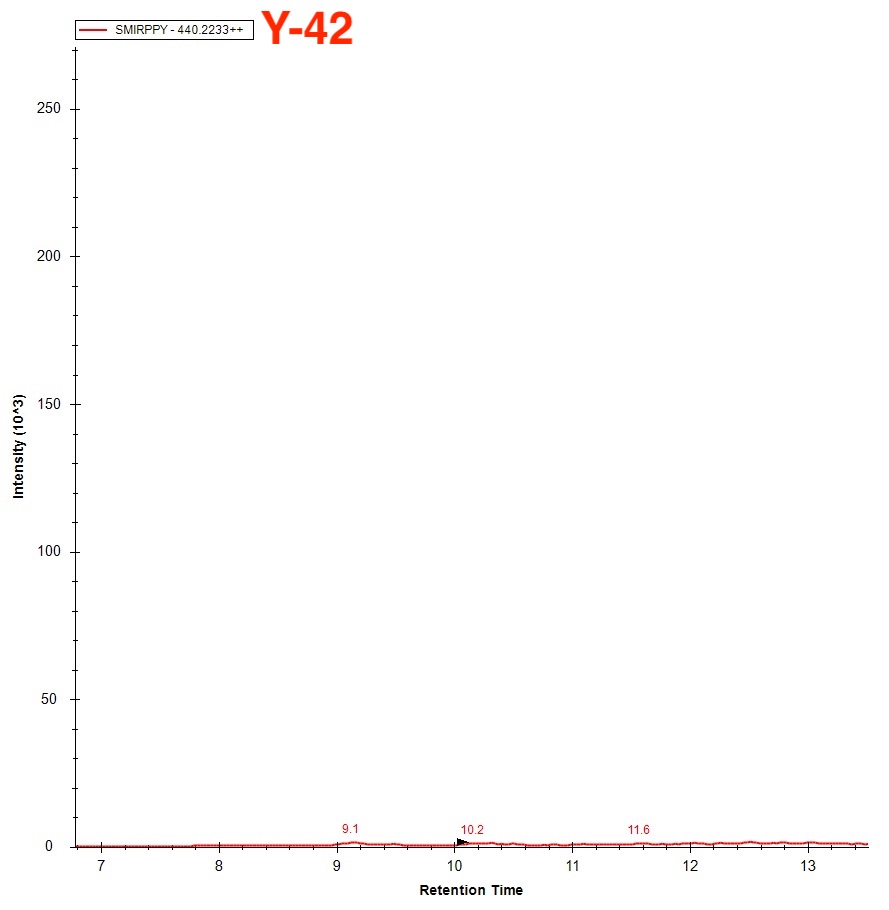


**Figure 48. Ion chromatogram of amino acid sequence SMIRPPY - 440.2233++ (AMELY), individual 44.**


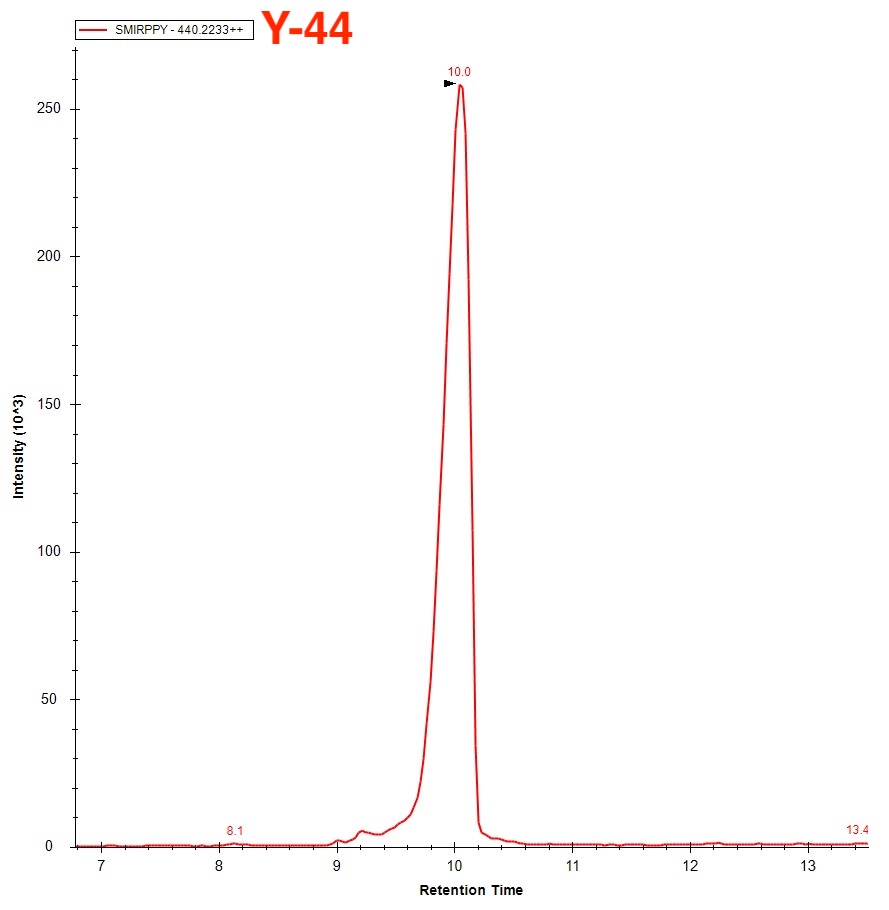


**Figure 49. Ion chromatogram of amino acid sequence SMIRPPY - 440.2233++ (AMELY), individual 46.**


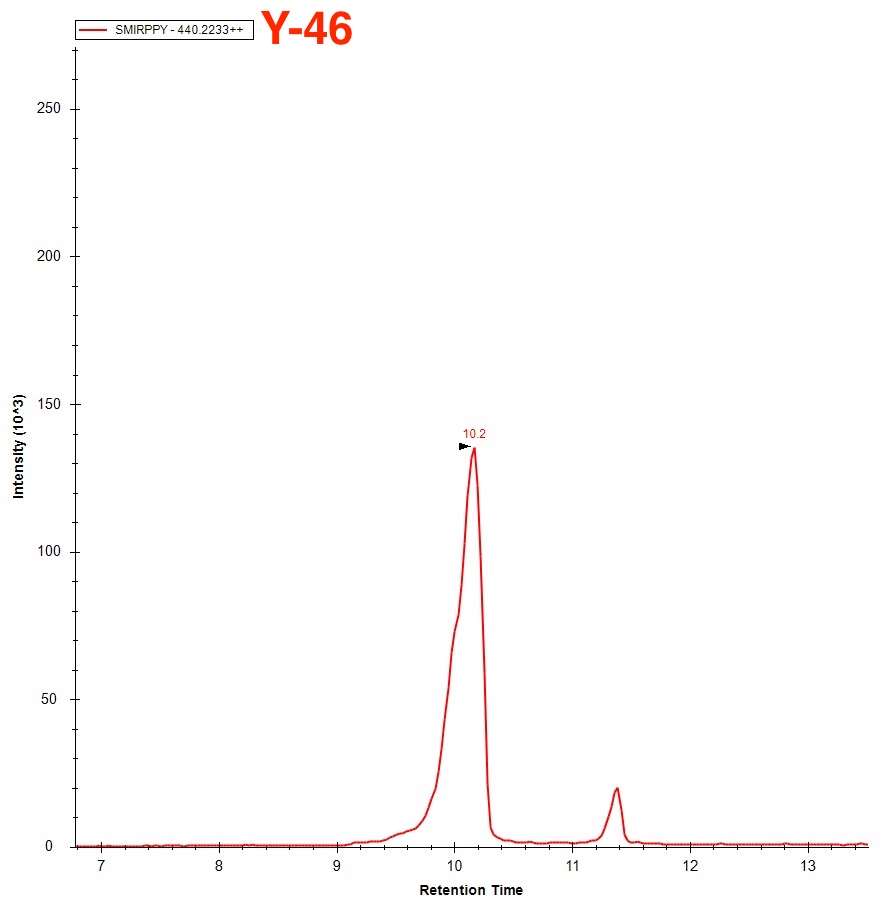


**Figure 50. Ion chromatogram of amino acid sequence SMIRPPY - 440.2233++ (AMELY), individual 54.5.**


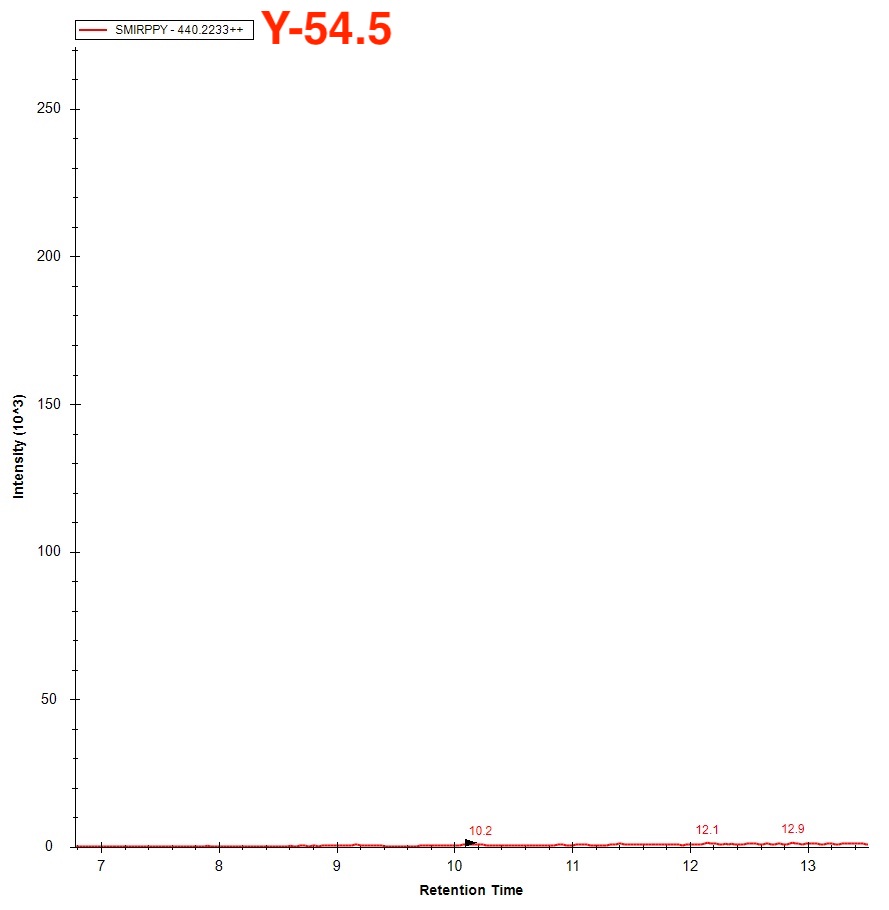


**Figure 51. Ion chromatogram of amino acid sequence SMIRPPY - 440.2233++ (AMELY), individual SLR.**


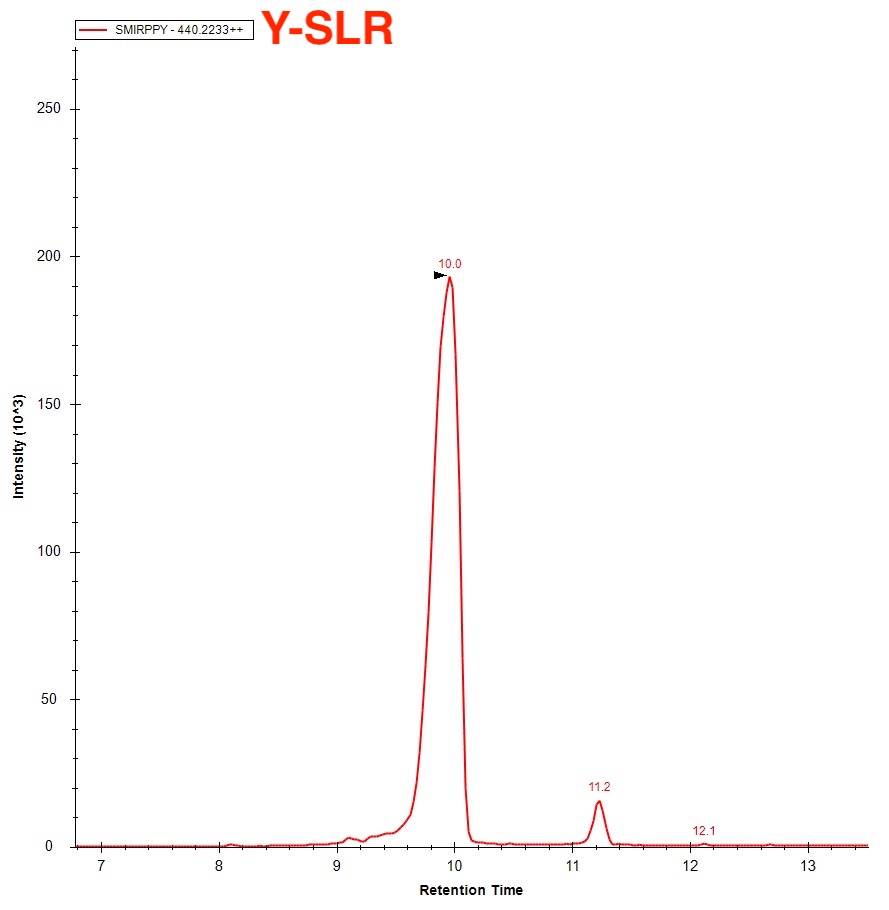


**Figure 52. Ion chromatogram of amino acid sequence SMIRPPY - 440.2233++ (AMELY), individual SSJ.**


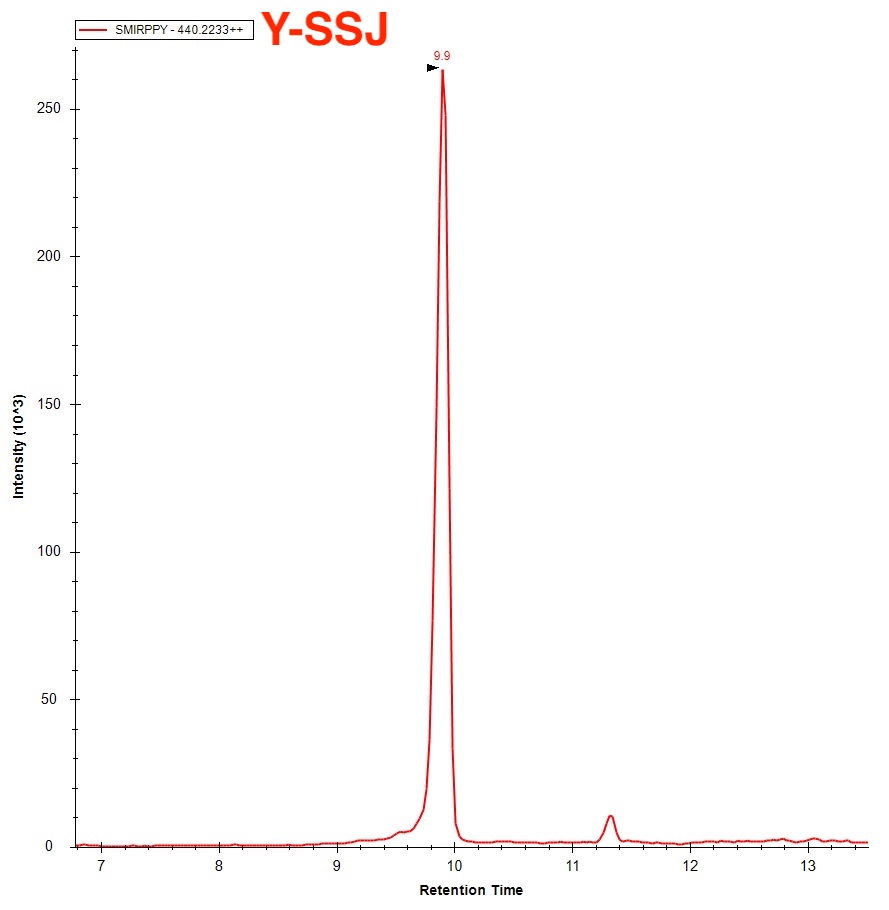


**AMELX ion chromatograms:**

**Figure 53. Ion chromatogram of amino acid sequence SIRPPYPSY - 540.2796++ (AMELX), individual 1.**


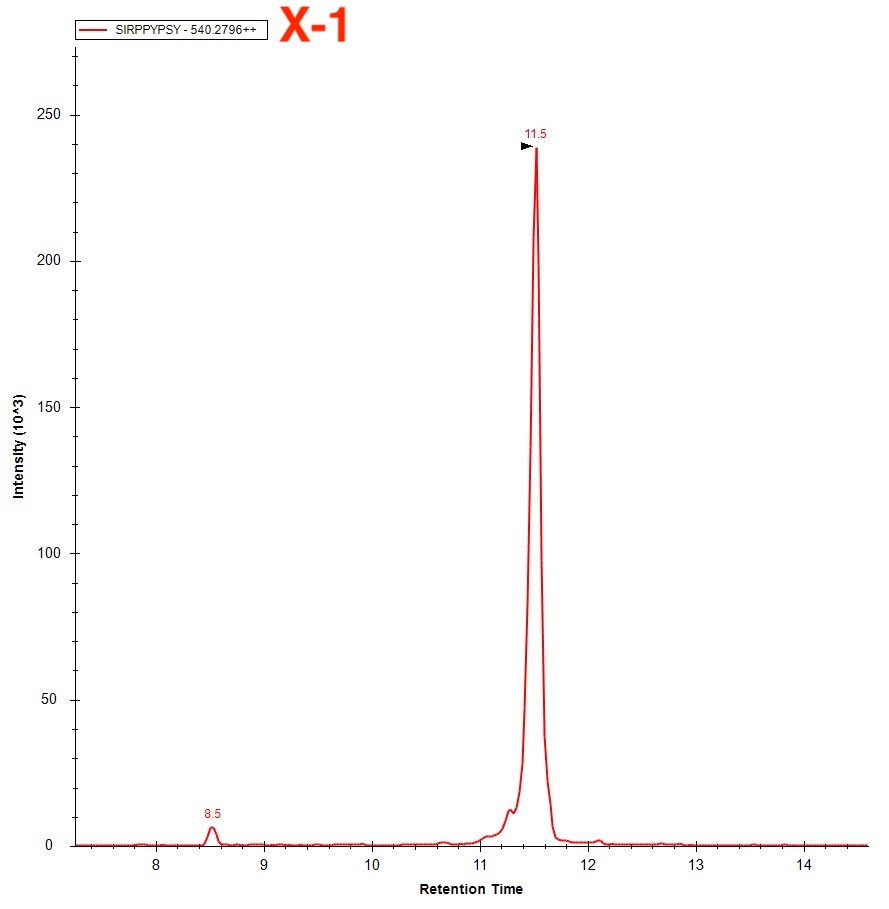


**Figure 54. Ion chromatogram of amino acid sequence SIRPPYPSY - 540.2796++ (AMELX), individual 2.**
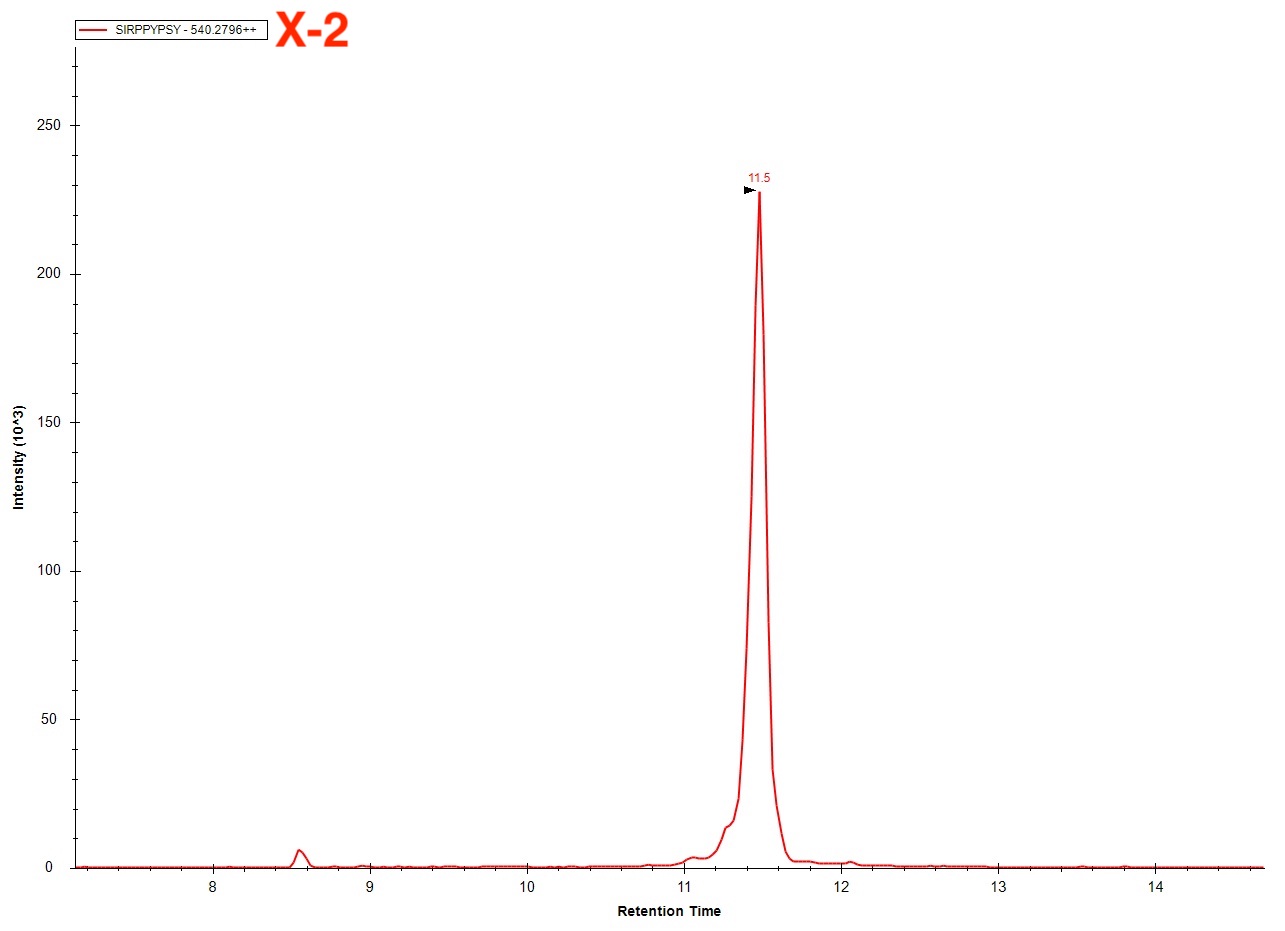


**Figure 55. Ion chromatogram of amino acid sequence SIRPPYPSY - 540.2796++ (AMELX), individual 4.**


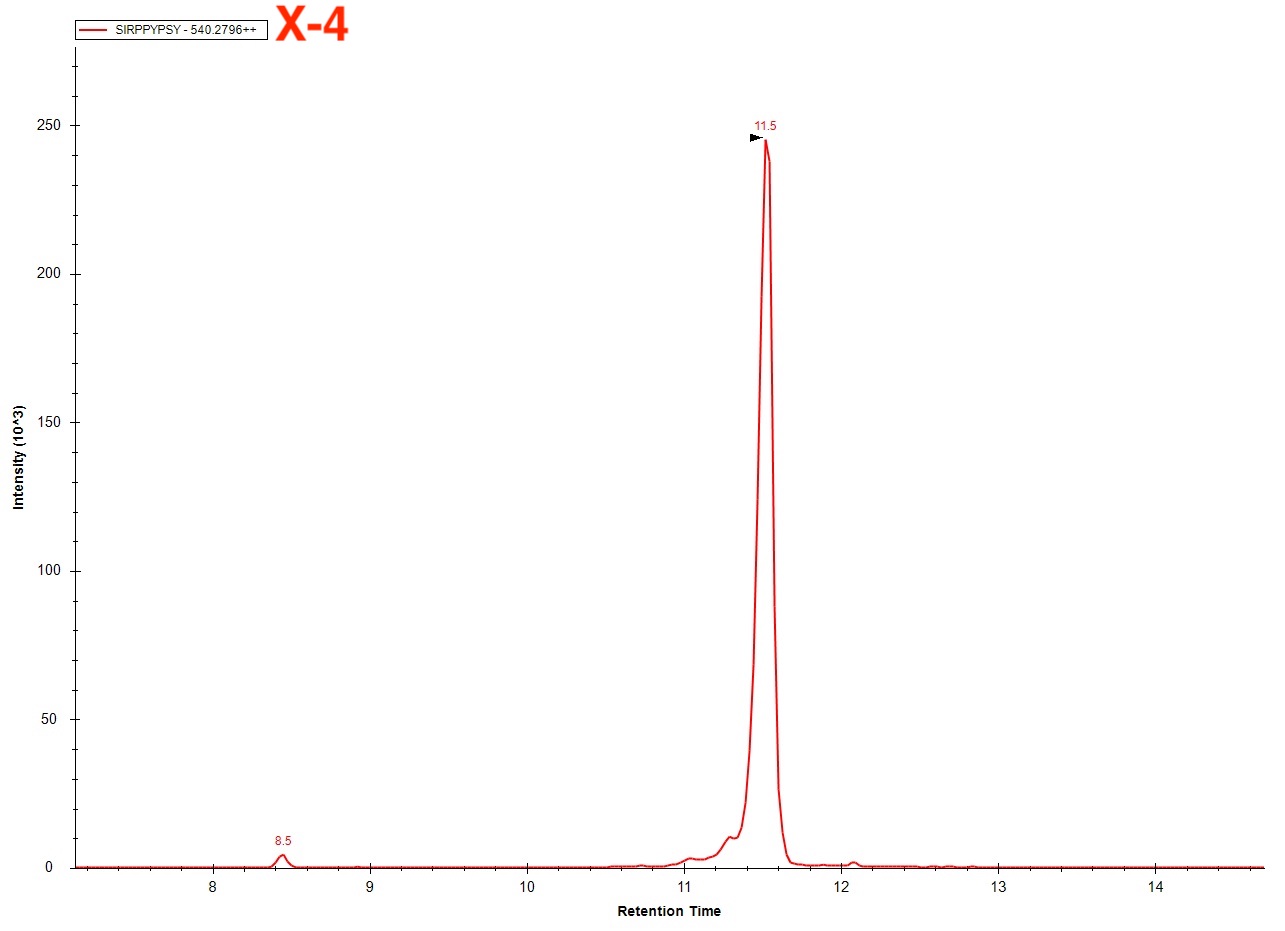


**Figure 56. Ion chromatogram of amino acid sequence SIRPPYPSY - 540.2796++ (AMELX), individual 6.**
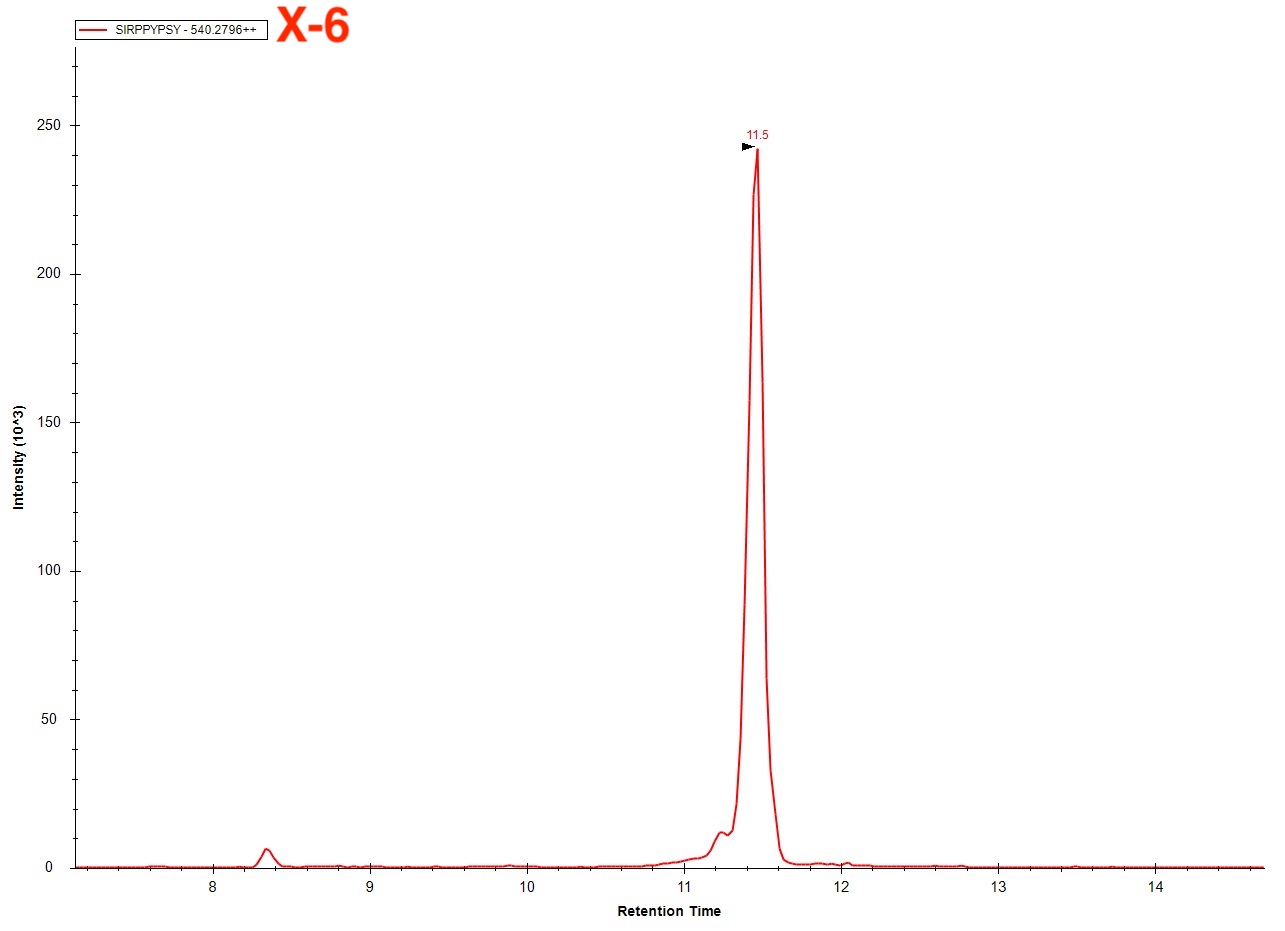


**Figure 57. Ion chromatogram of amino acid sequence SIRPPYPSY - 540.2796++ (AMELX), individual 7.**


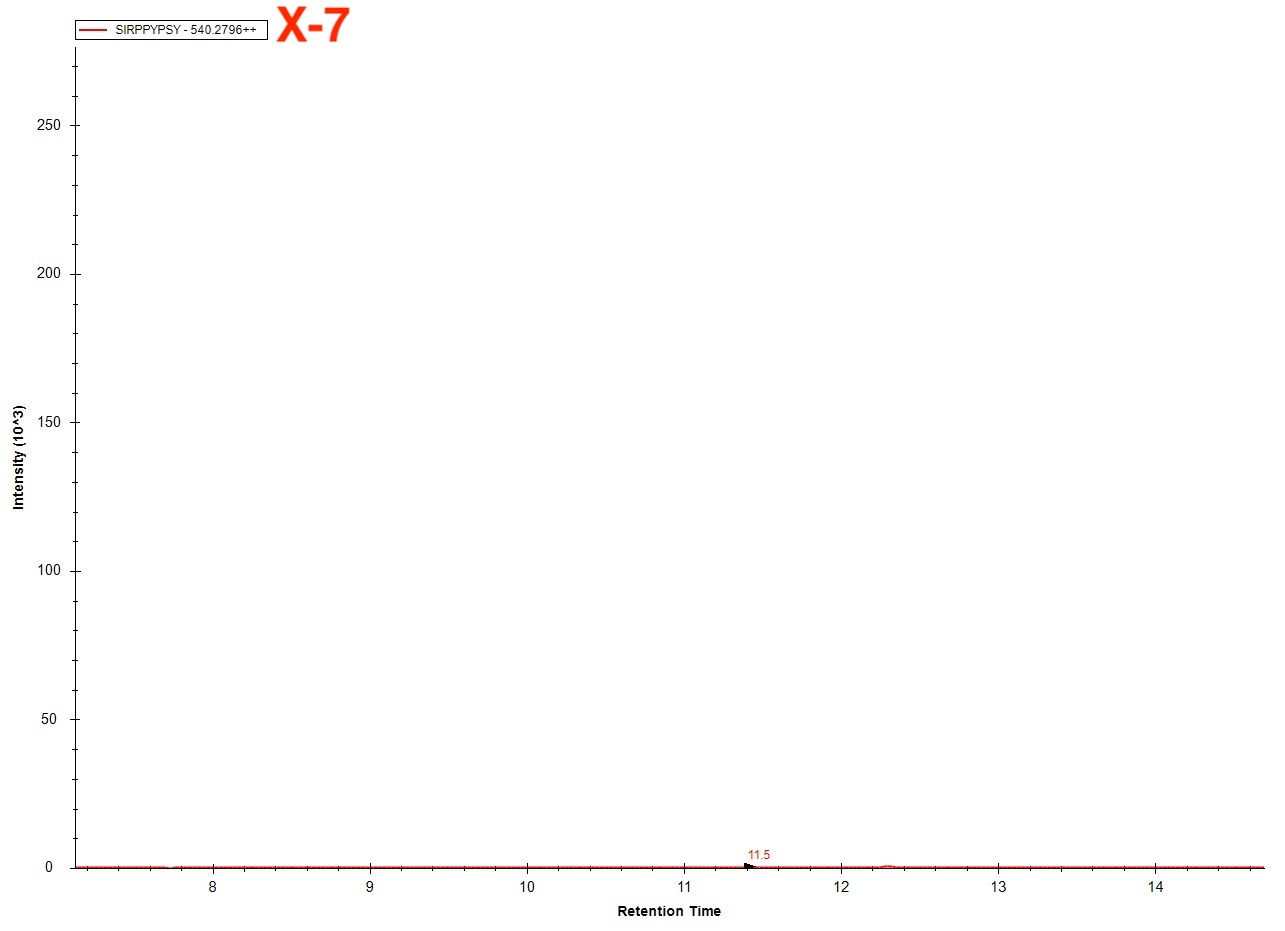


**Figure 58. Ion chromatogram of amino acid sequence SIRPPYPSY - 540.2796++ (AMELX), individual 9.**


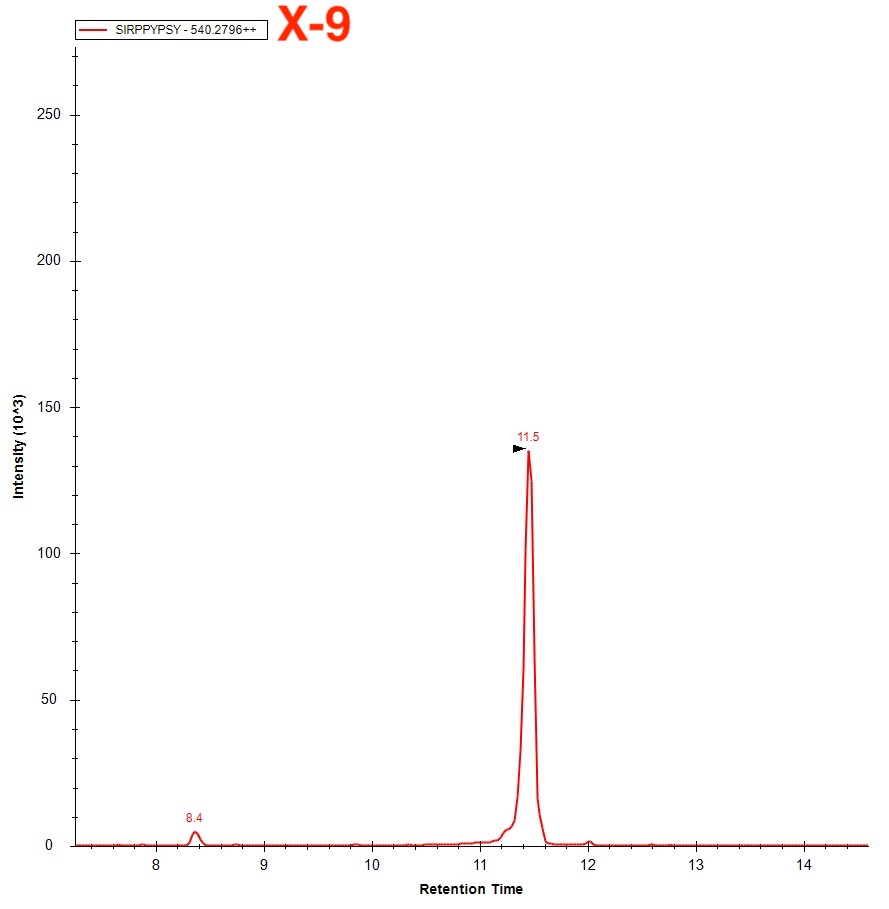


**Figure 59. Ion chromatogram of amino acid sequence SIRPPYPSY - 540.2796++ (AMELX), individual 13.**
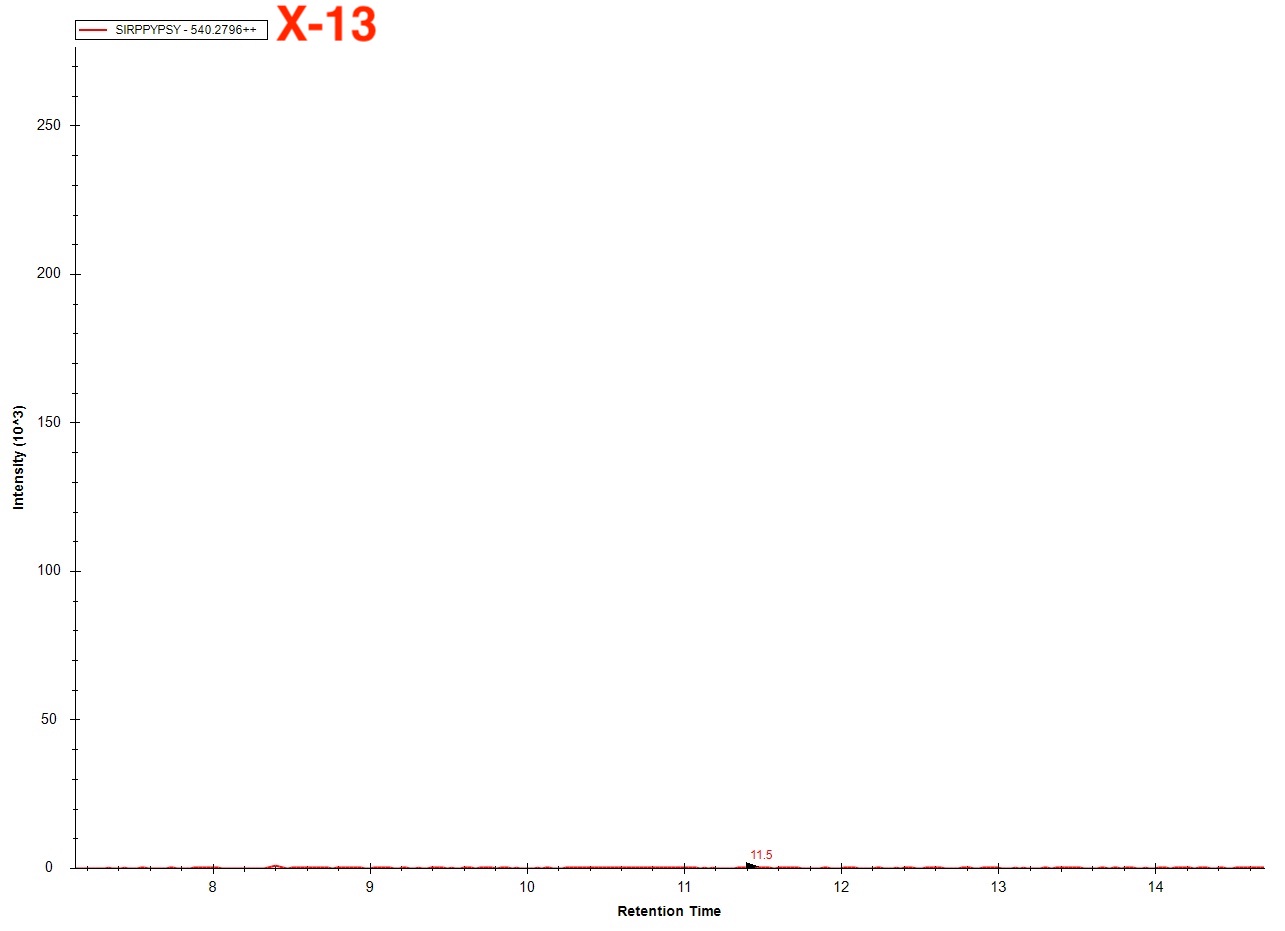


**Figure 60. Ion chromatogram of amino acid sequence SIRPPYPSY - 540.2796++ (AMELX), individual 14.**


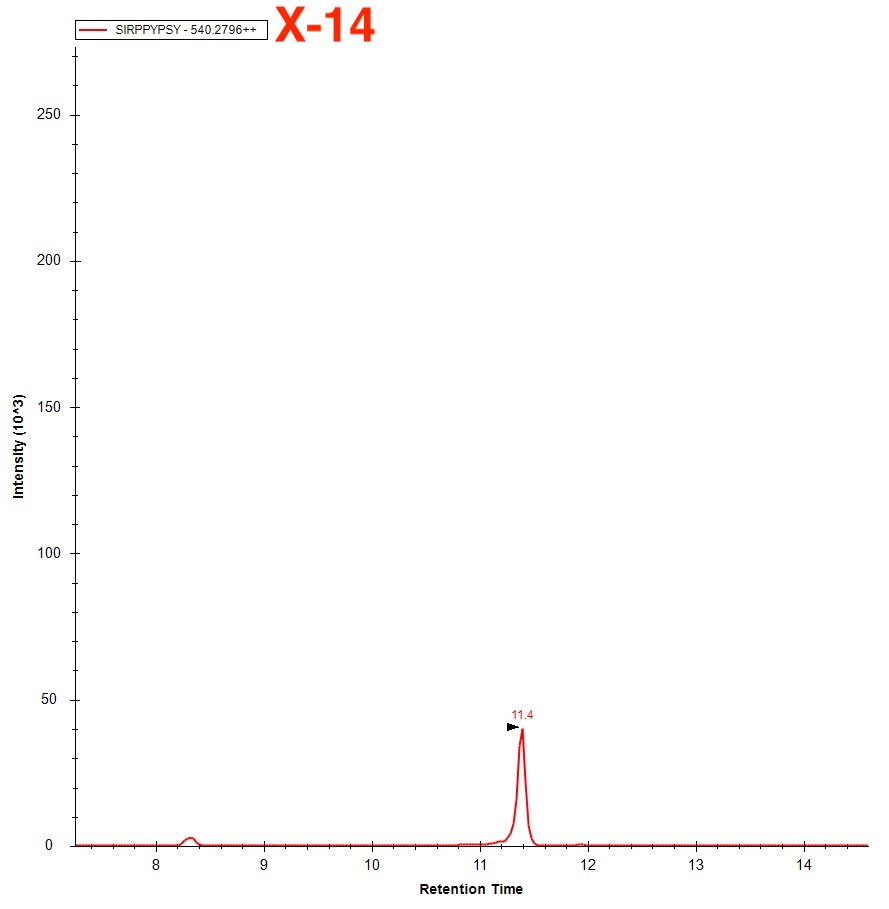


**Figure 61. Ion chromatogram of amino acid sequence SIRPPYPSY - 540.2796++ (AMELX), individual 15.**


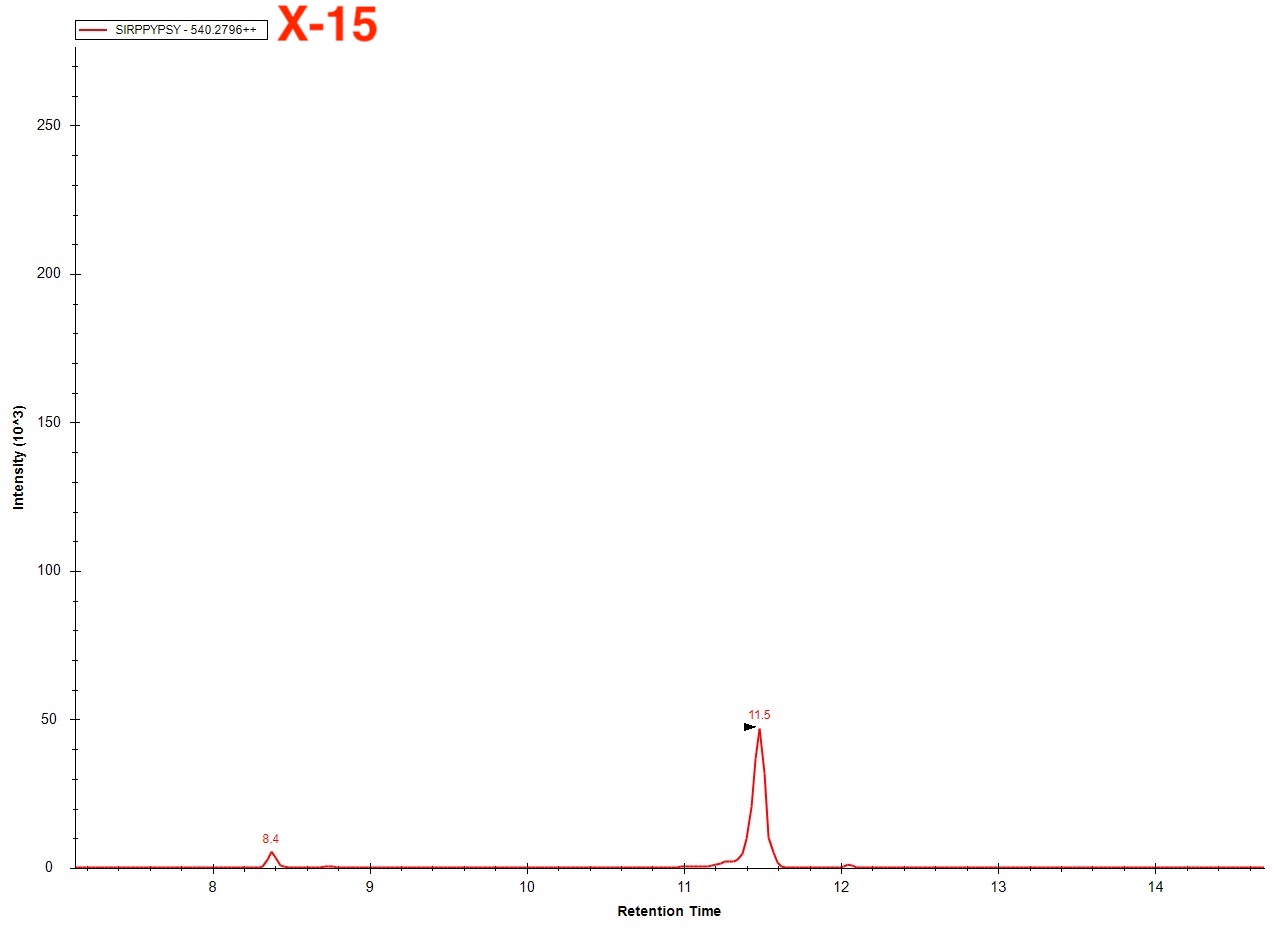


**Figure 62. Ion chromatogram of amino acid sequence SIRPPYPSY - 540.2796++ (AMELX), individual 16.**


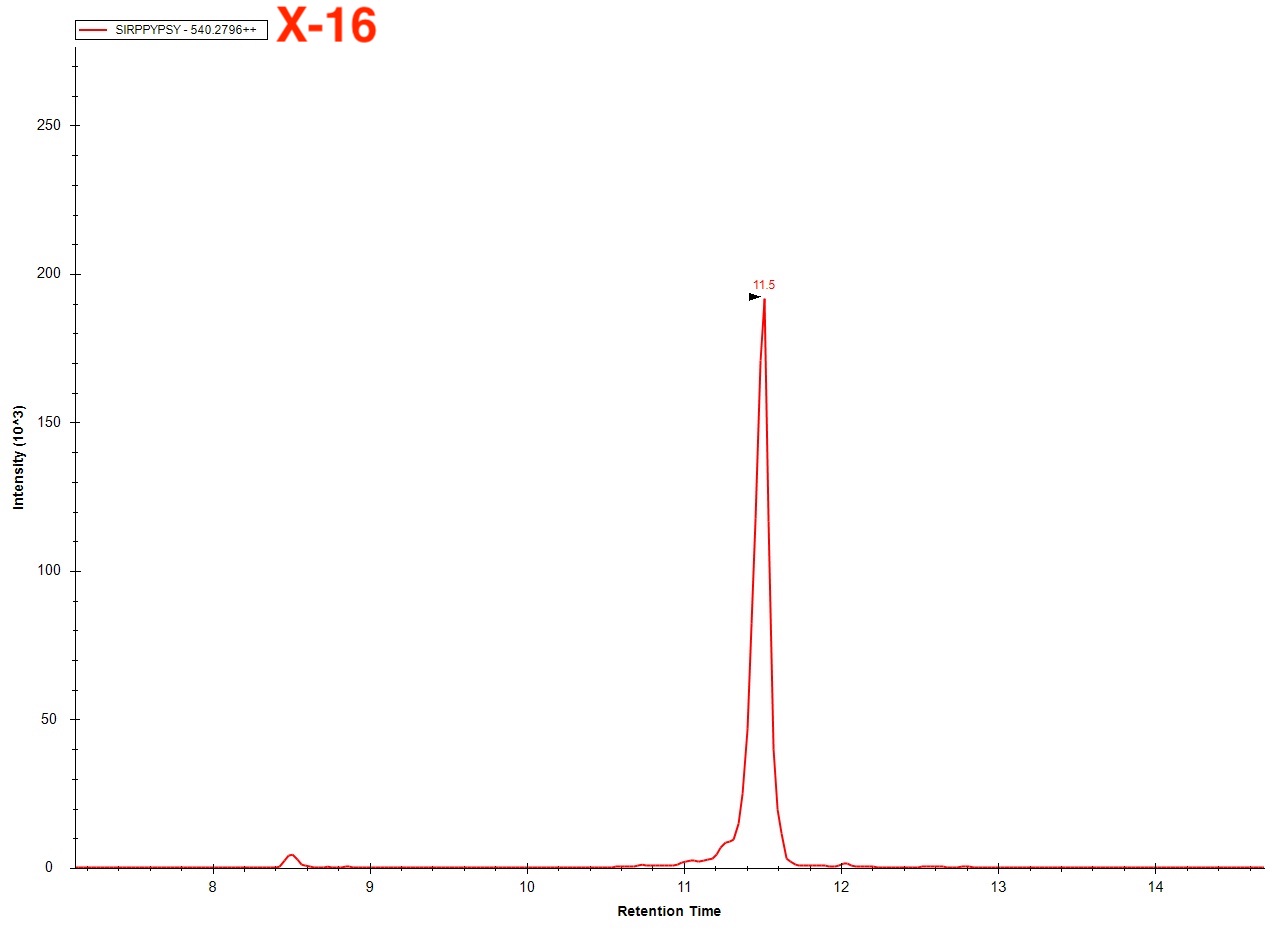


**Figure 63. Ion chromatogram of amino acid sequence SIRPPYPSY - 540.2796++ (AMELX), individual 17.**


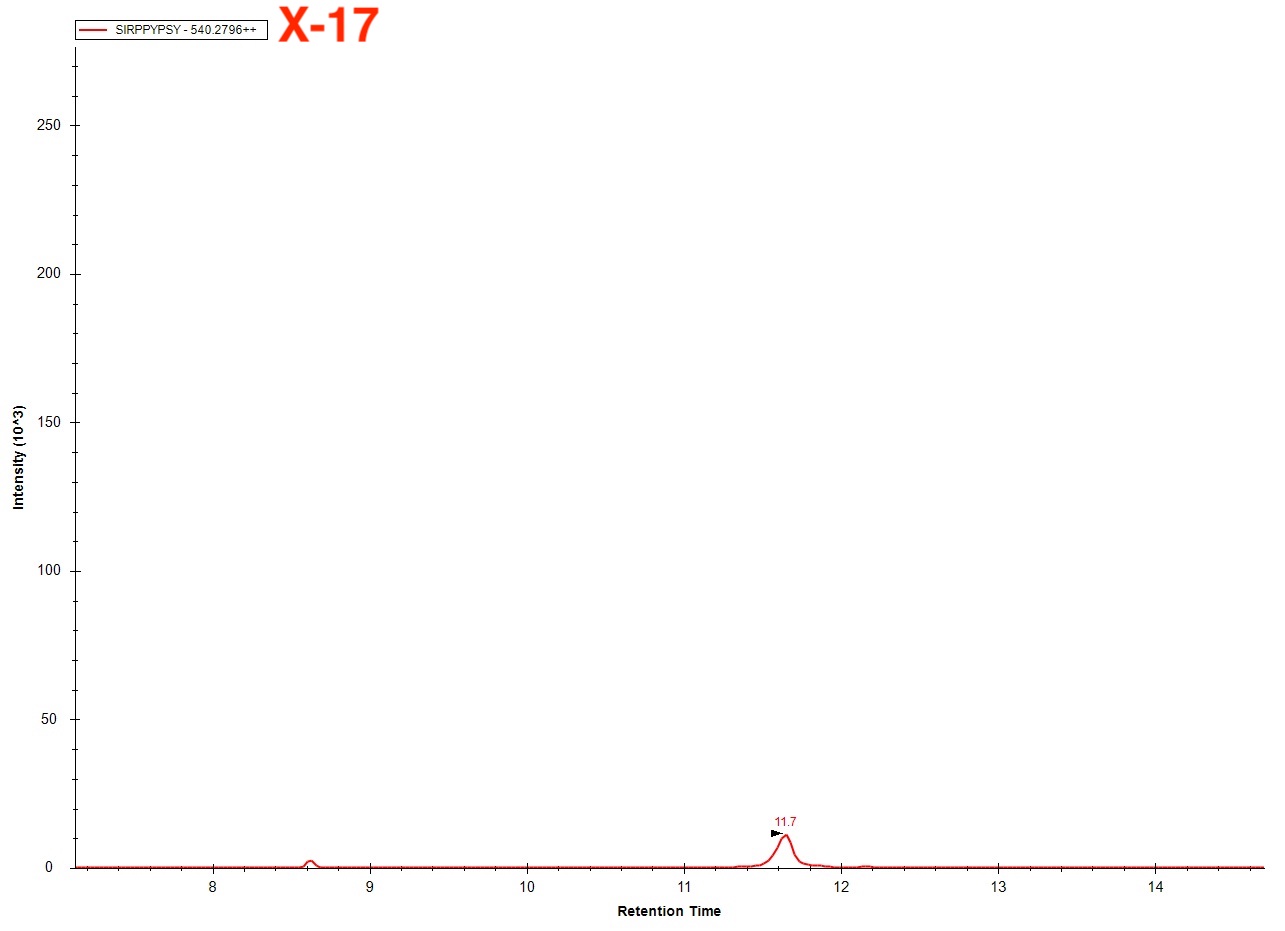


**Figure 64. Ion chromatogram of amino acid sequence SIRPPYPSY - 540.2796++ (AMELX), individual 20.**
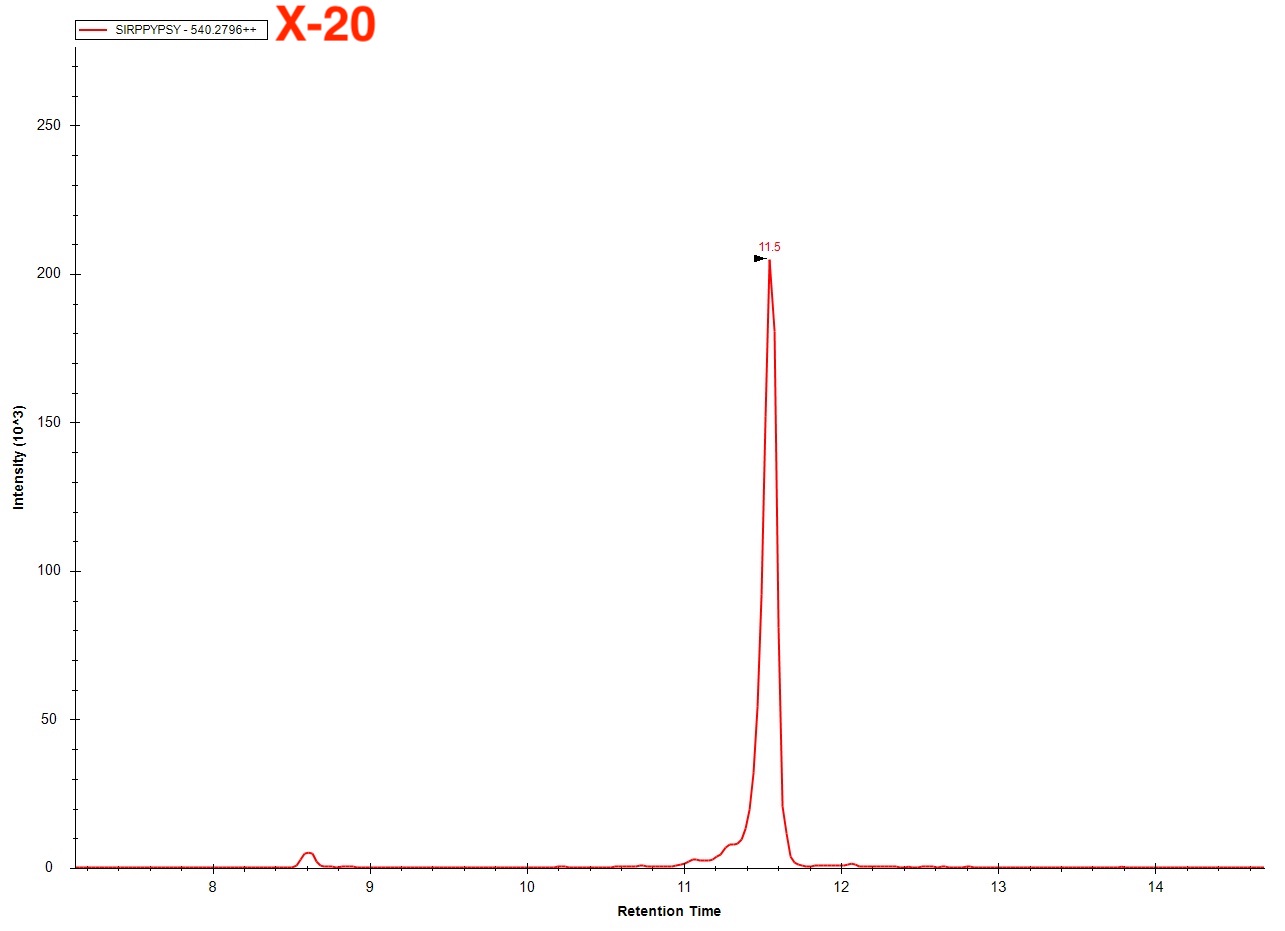


**Figure 65. Ion chromatogram of amino acid sequence SIRPPYPSY - 540.2796++ (AMELX), individual 21.**


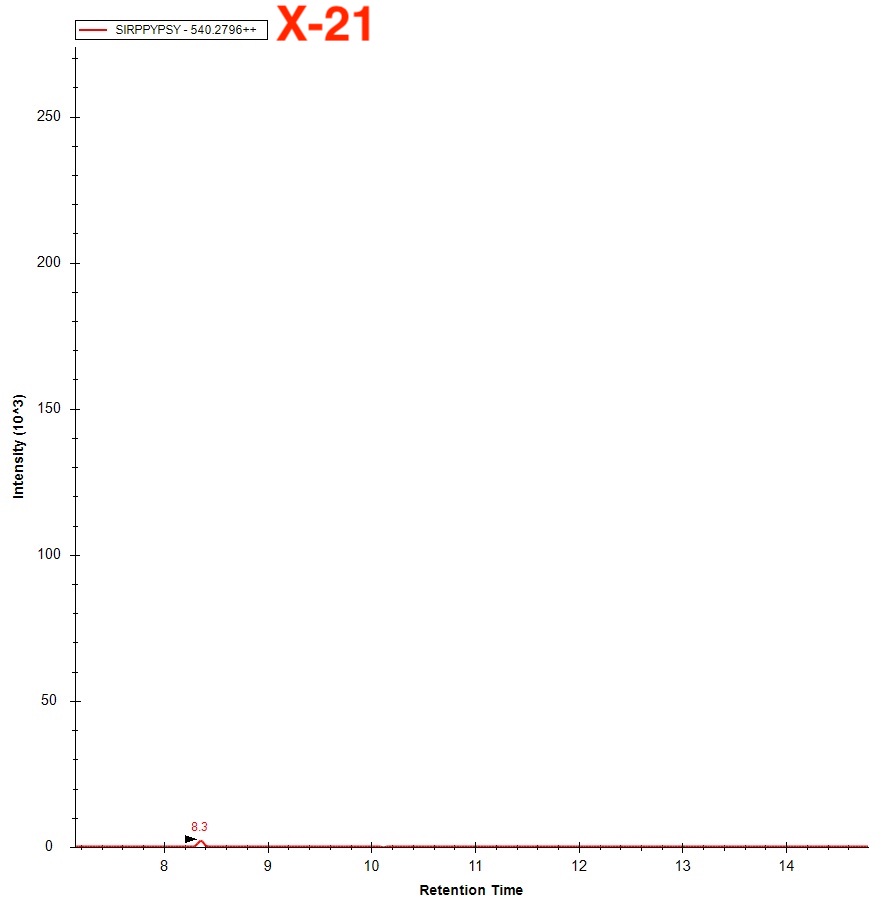


**Figure 66. Ion chromatogram of amino acid sequence SIRPPYPSY - 540.2796++ (AMELX), individual 22.**


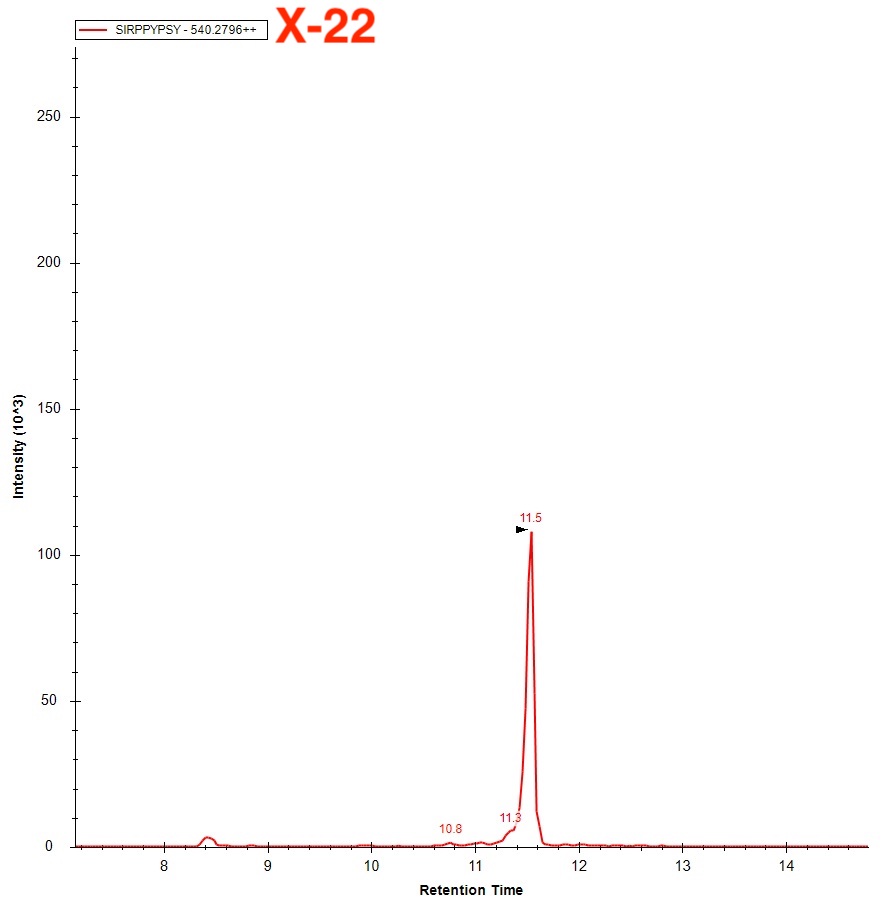


**Figure 67. Ion chromatogram of amino acid sequence SIRPPYPSY - 540.2796++ (AMELX), individual 26.**


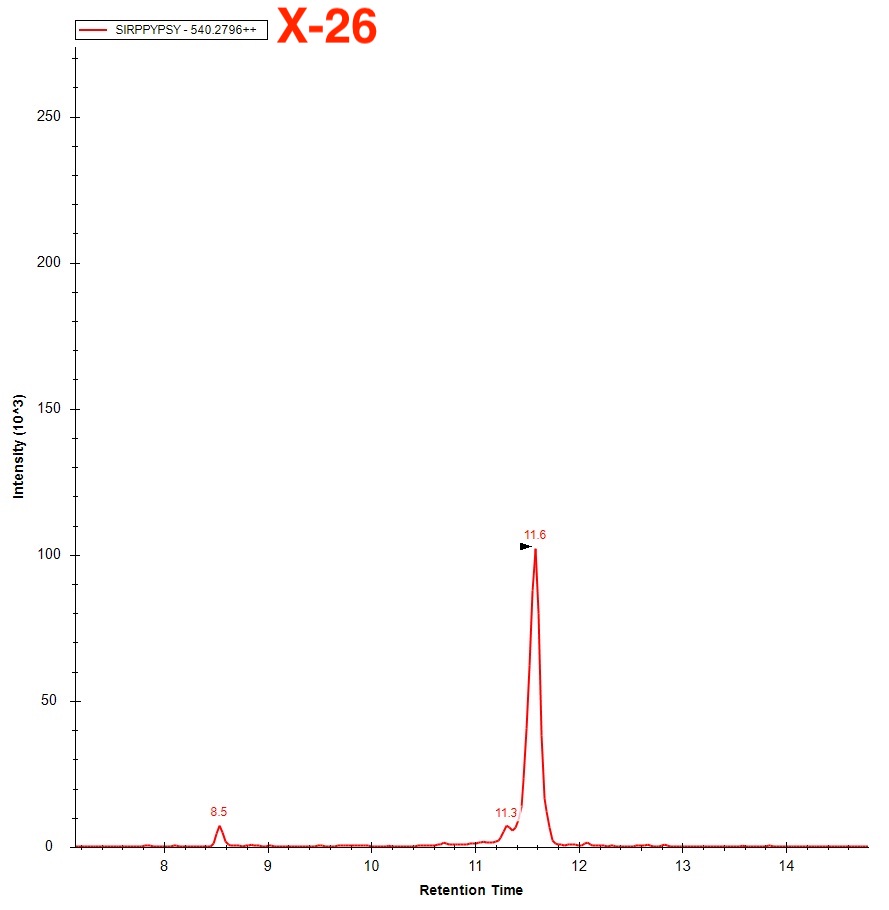


**Figure 68. Ion chromatogram of amino acid sequence SIRPPYPSY - 540.2796++ (AMELX), individual 36.**


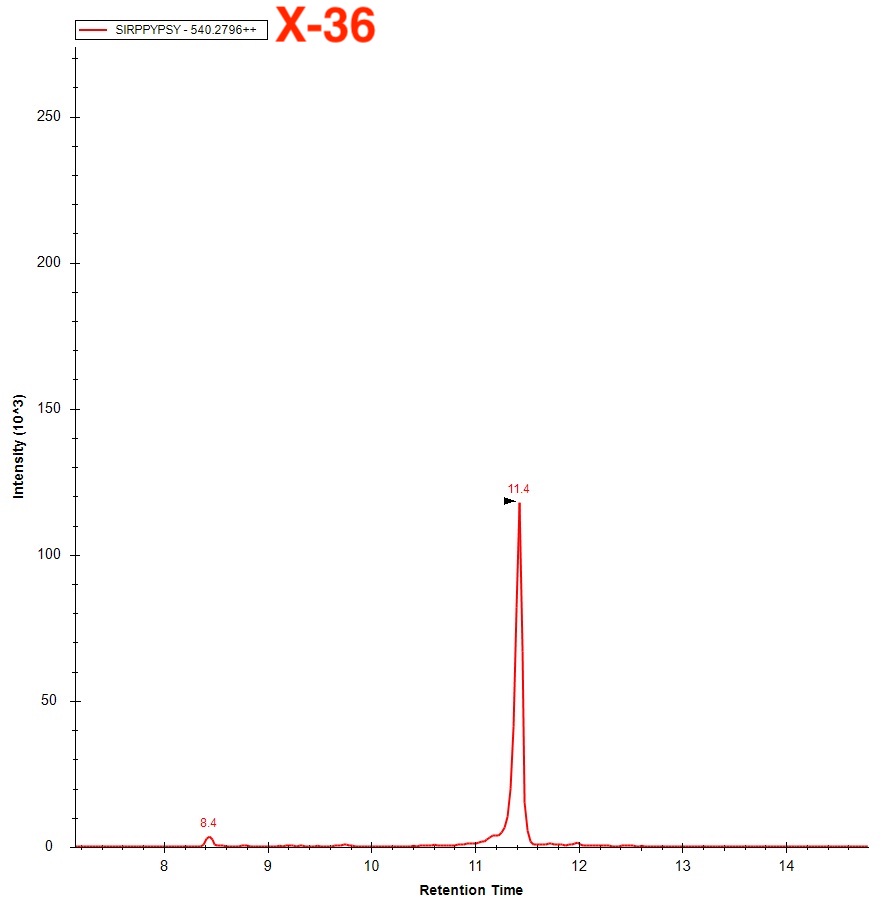


**Figure 69. Ion chromatogram of amino acid sequence SIRPPYPSY - 540.2796++ (AMELX), individual 38.**


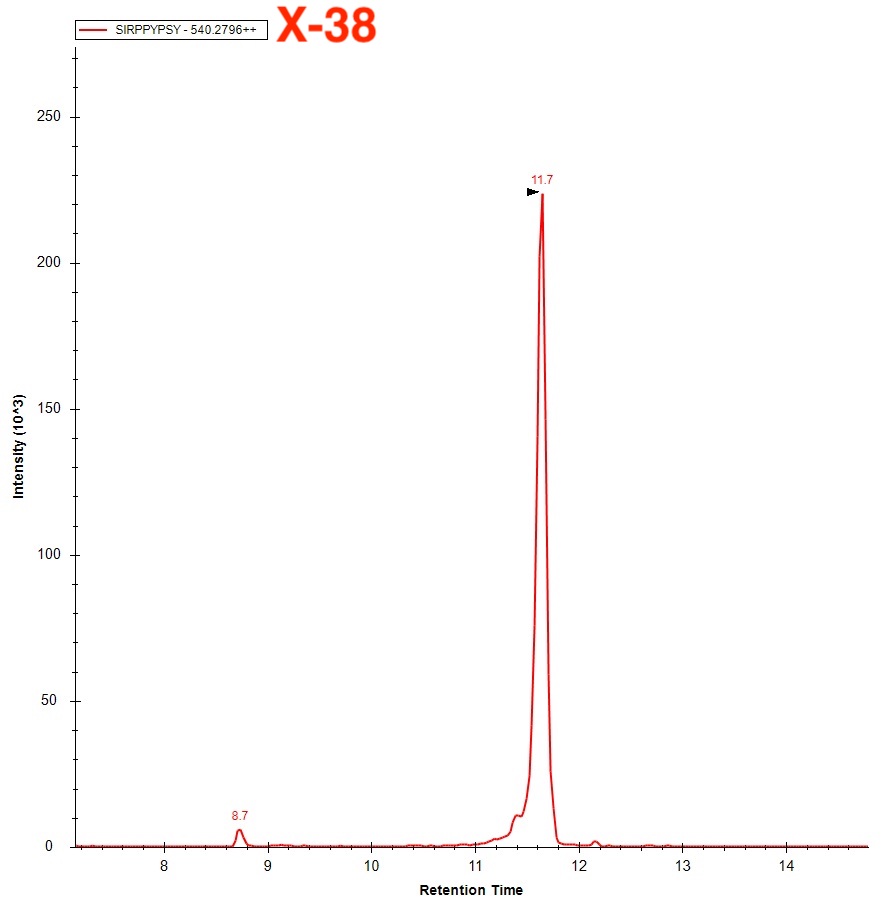


**Figure 70. Ion chromatogram of amino acid sequence SIRPPYPSY - 540.2796++ (AMELX), individual 39.**


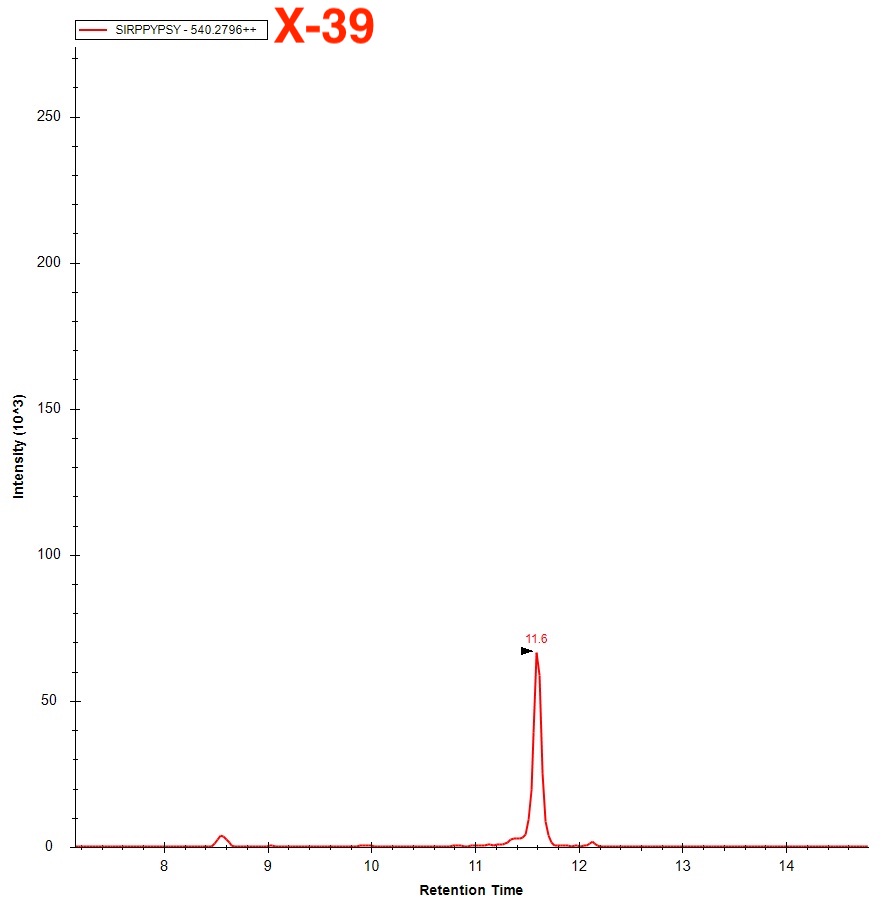


**Figure 71. Ion chromatogram of amino acid sequence SIRPPYPSY - 540.2796++ (AMELX), individual 40.**


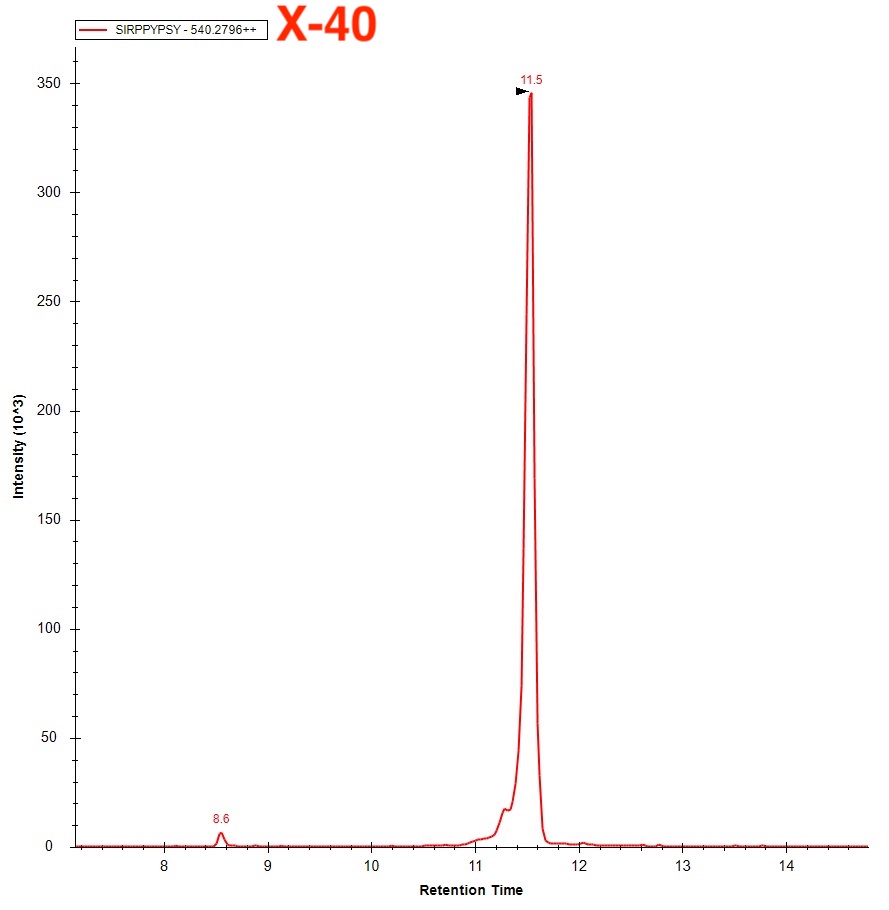


**Figure 72. Ion chromatogram of amino acid sequence SIRPPYPSY - 540.2796++ (AMELX), individual 41.**


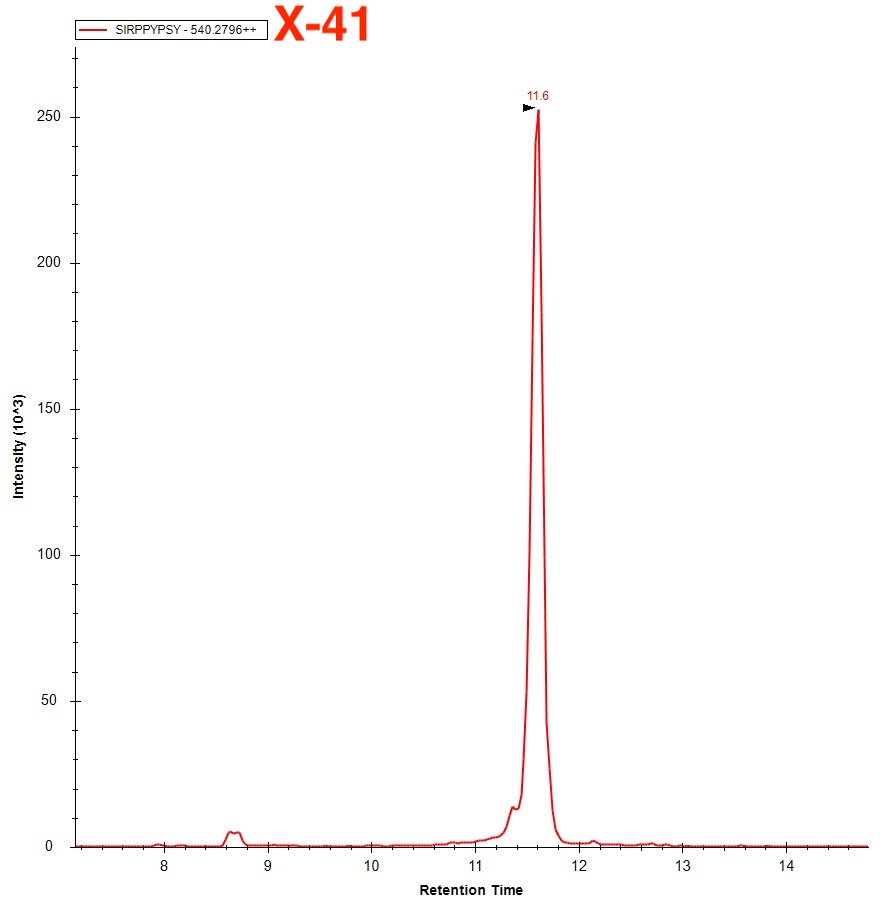


**Figure 73. Ion chromatogram of amino acid sequence SIRPPYPSY - 540.2796++ (AMELX), individual 42.**


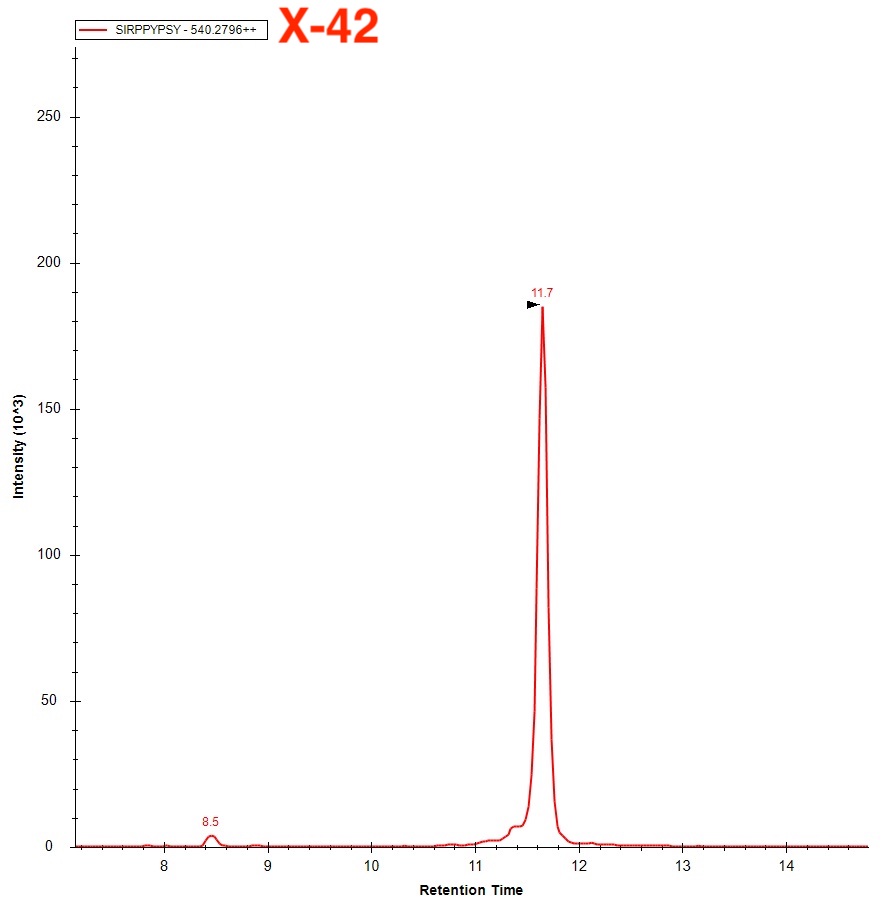


**Figure 74. Ion chromatogram of amino acid sequence SIRPPYPSY - 540.2796++ (AMELX), individual 44.**


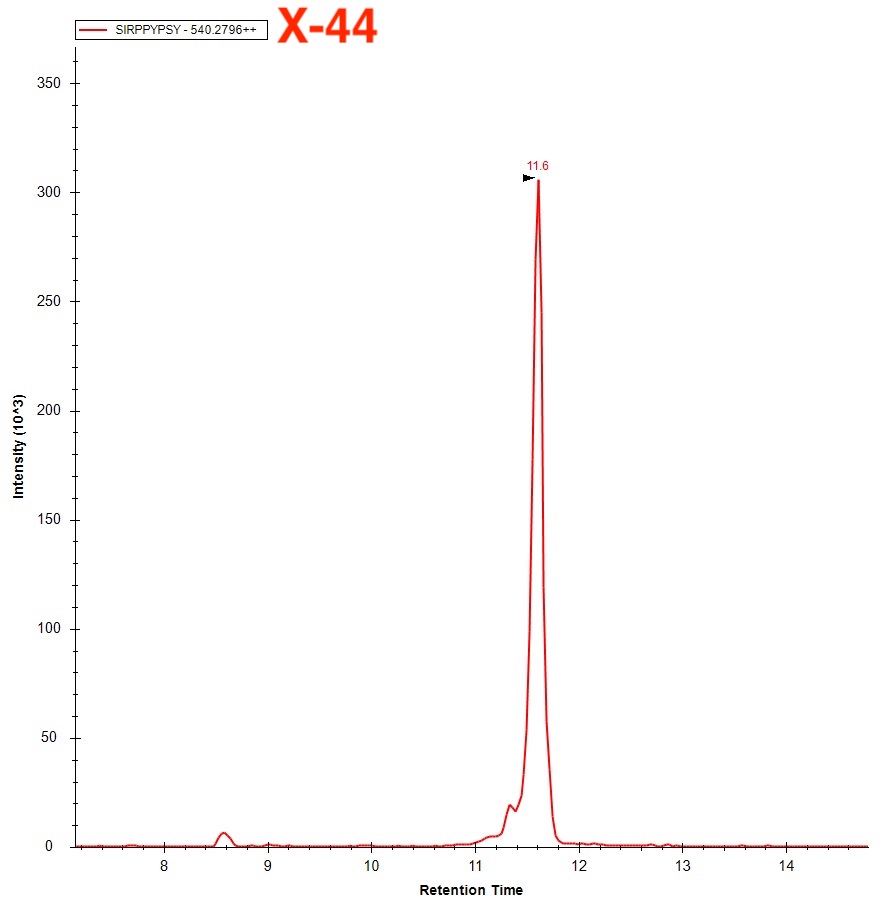


**Figure 75. Ion chromatogram of amino acid sequence SIRPPYPSY - 540.2796++ (AMELX), individual 46.**


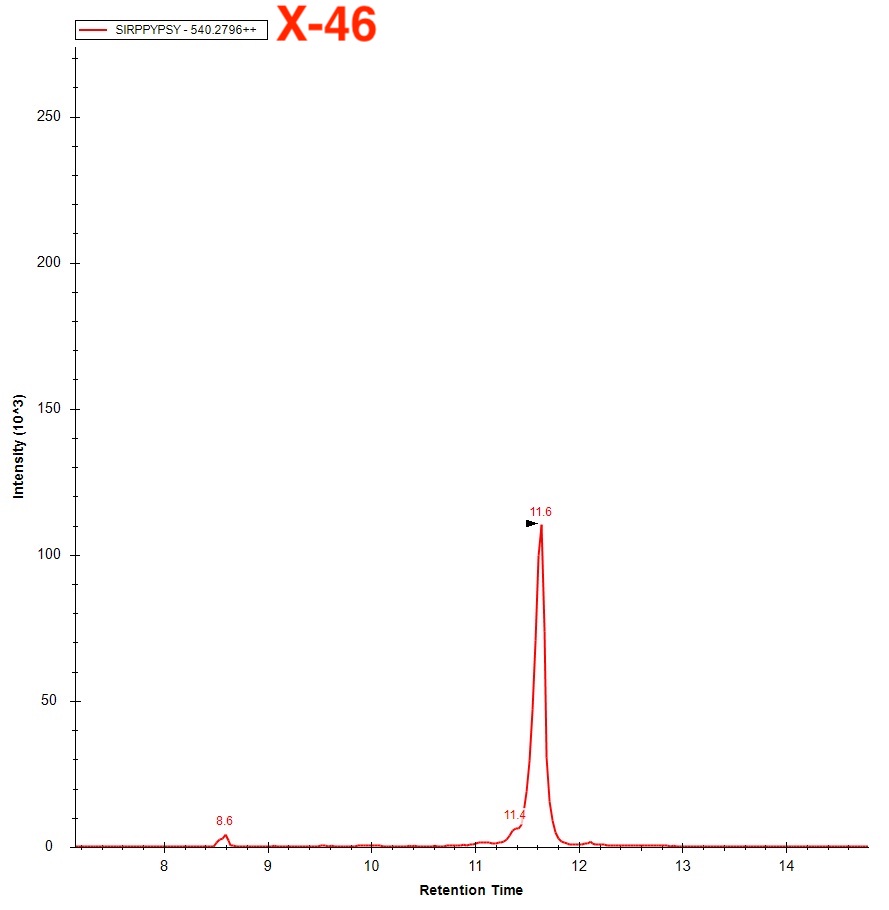


**Figure 76. Ion chromatogram of amino acid sequence SIRPPYPSY - 540.2796++ (AMELX), individual 54.5.**


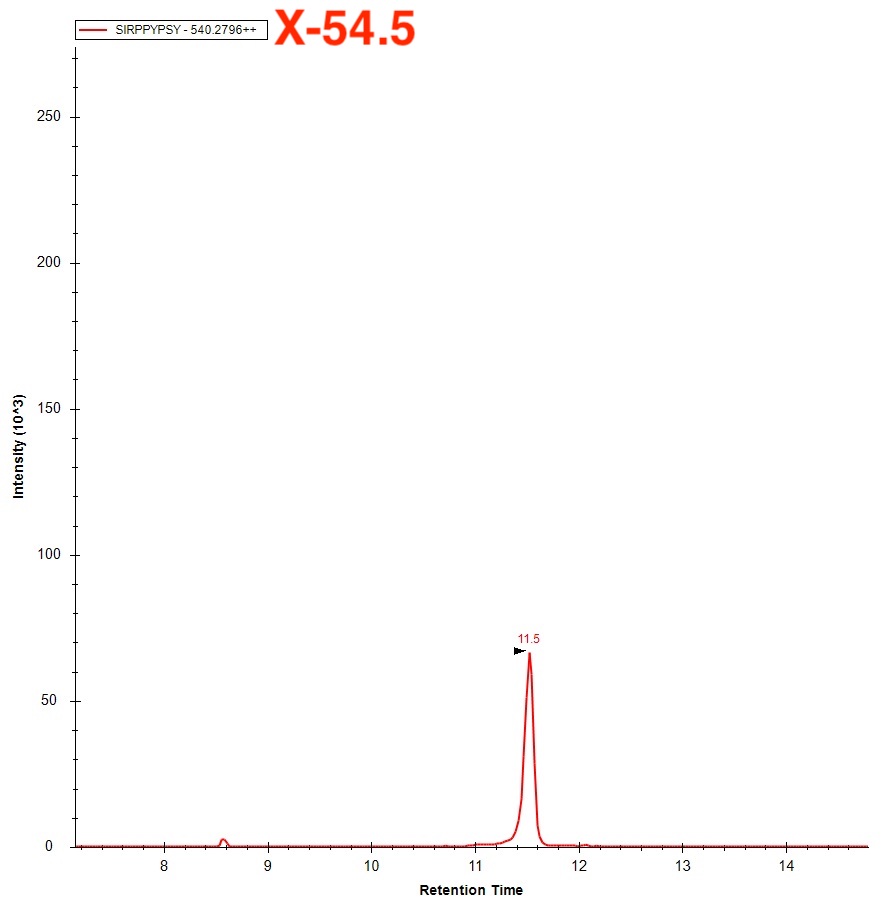


**Figure 77. Ion chromatogram of amino acid sequence SIRPPYPSY - 540.2796++ (AMELX), individual SLR.**


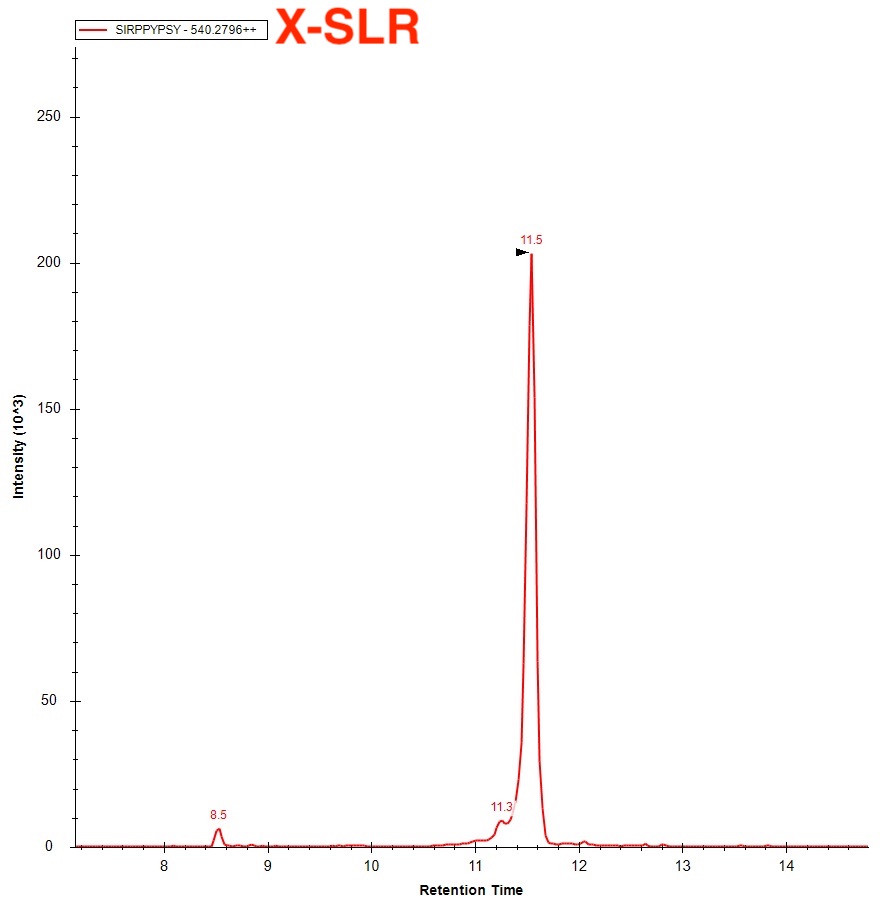


**Figure 78. Ion chromatogram of amino acid sequence SIRPPYPSY - 540.2796++ (AMELX), individual SSJ.**


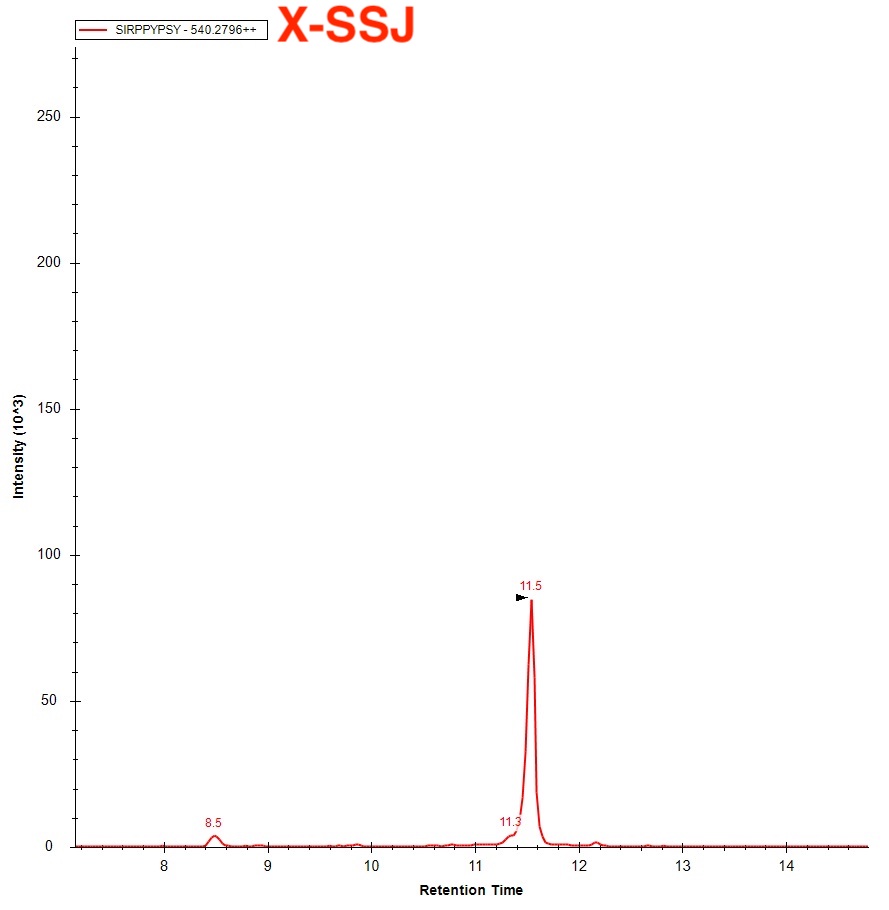


**Figure 79. Ion chromatogram of amino acid sequence SIRPPYP - 415.2320++ (AMELX), individual 1.**

**
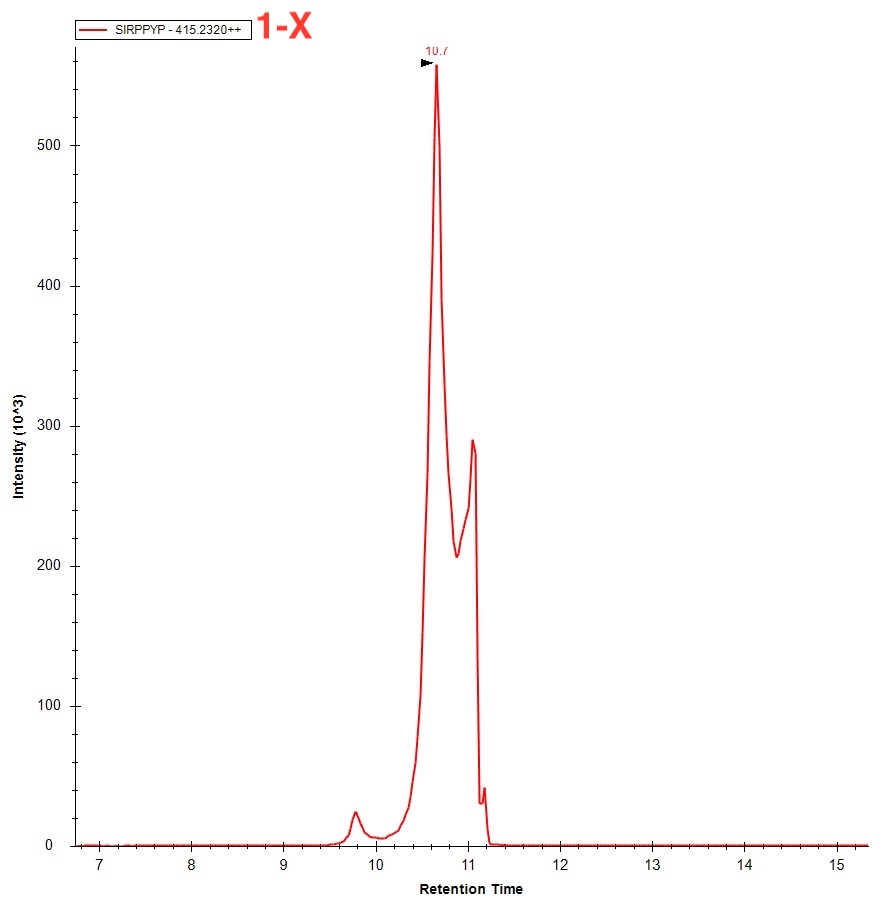
**

**Figure 80. Ion chromatogram of amino acid sequence SIRPPYP - 415.2320++ (AMELX), individual 2.**

**
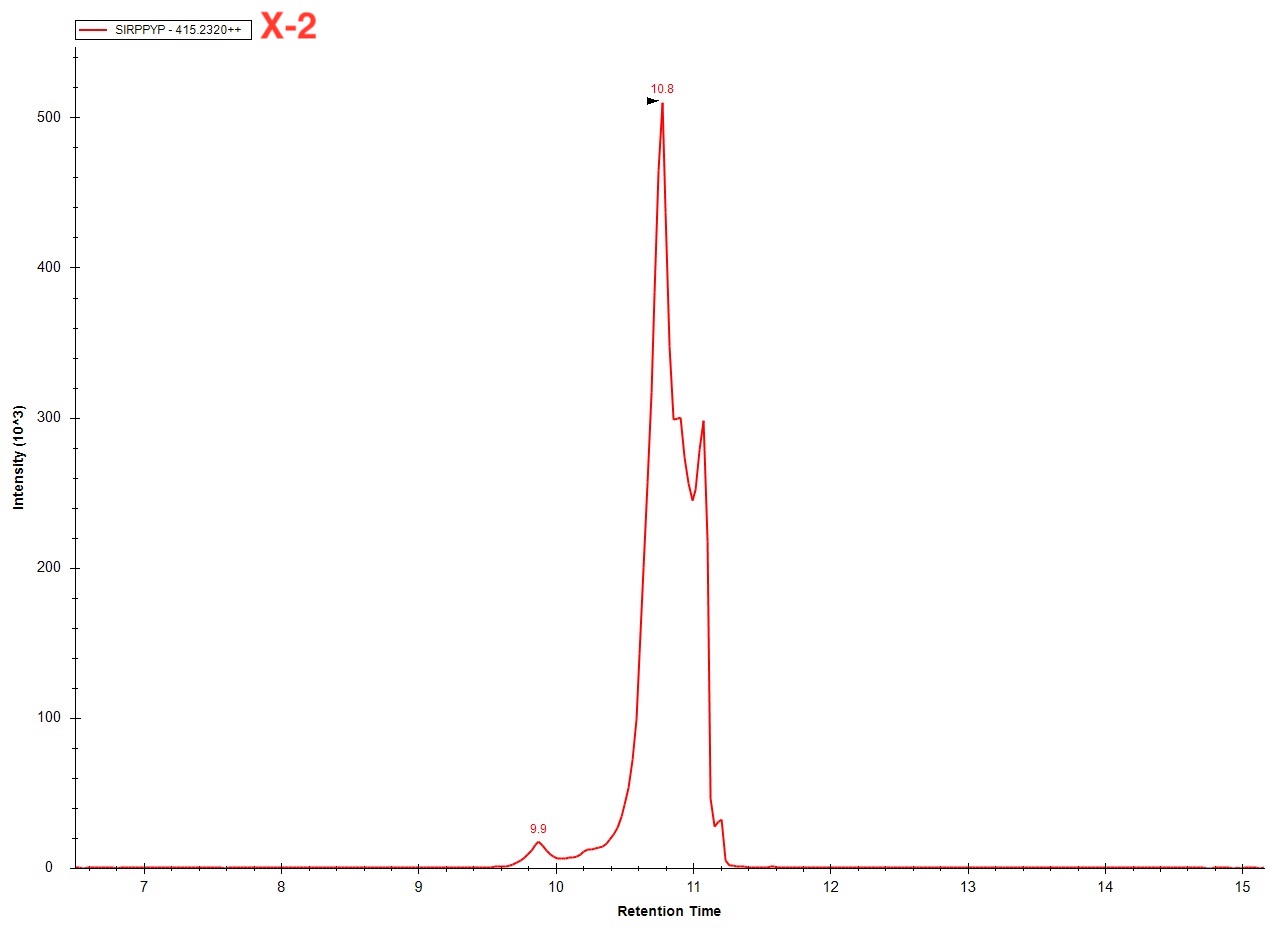
**

**Figure 81. Ion chromatogram of amino acid sequence SIRPPYP - 415.2320++ (AMELX), individual 4.**

**
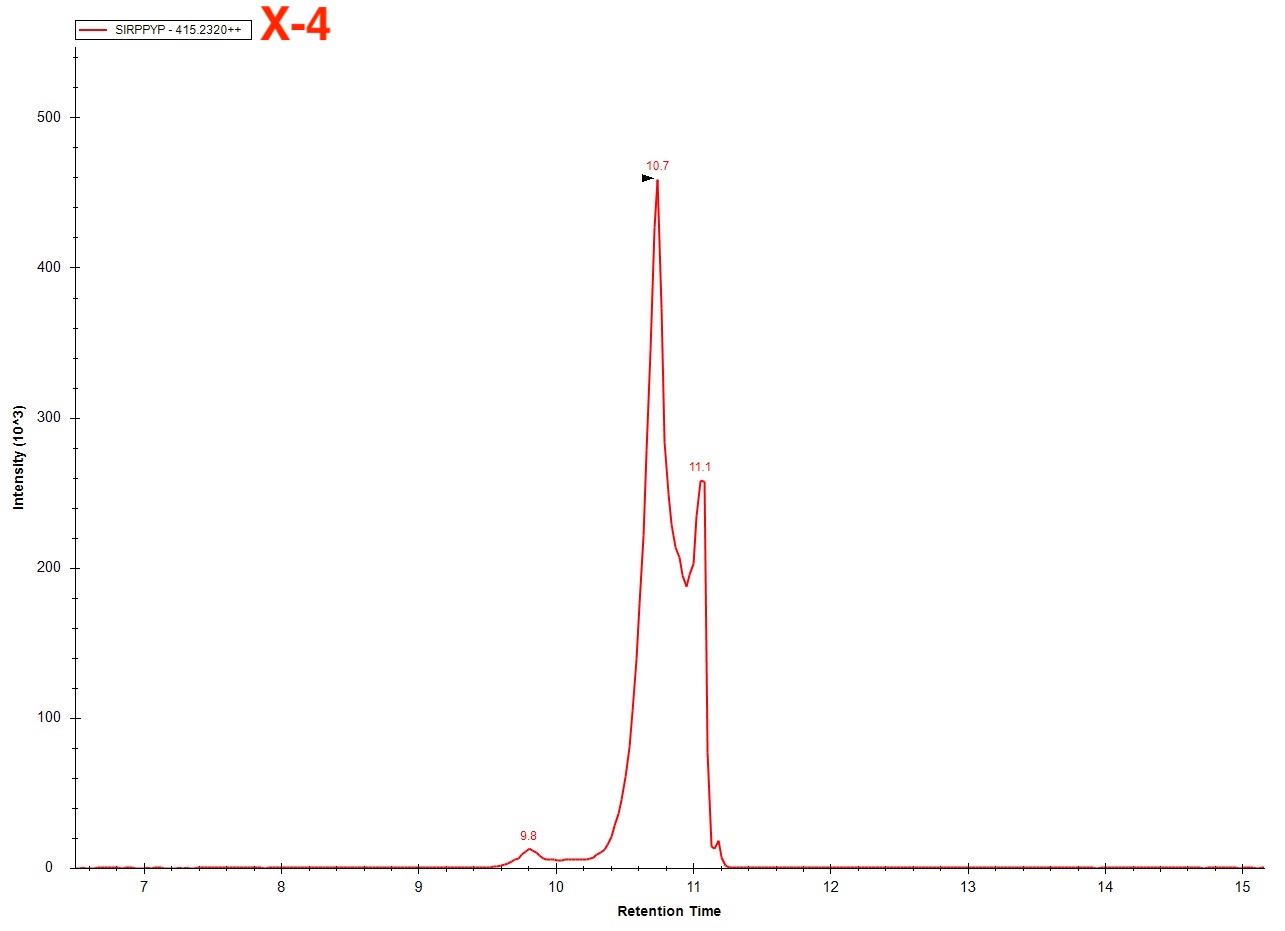
**

**Figure 82. Ion chromatogram of amino acid sequence SIRPPYP - 415.2320++ (AMELX), individual 6.**

**
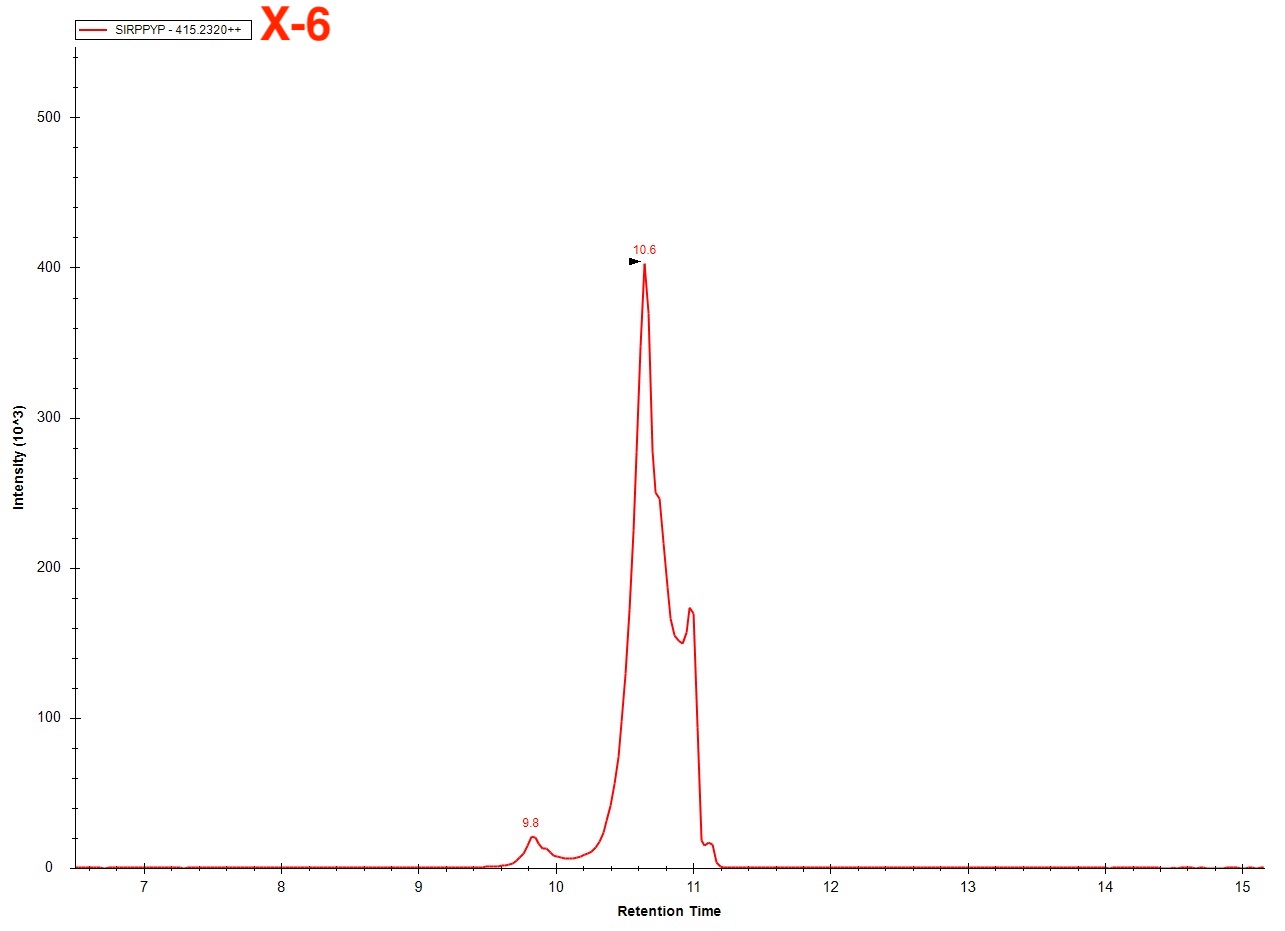
**

**Figure 83. Ion chromatogram of amino acid sequence SIRPPYP - 415.2320++ (AMELX), individual 7.**

**
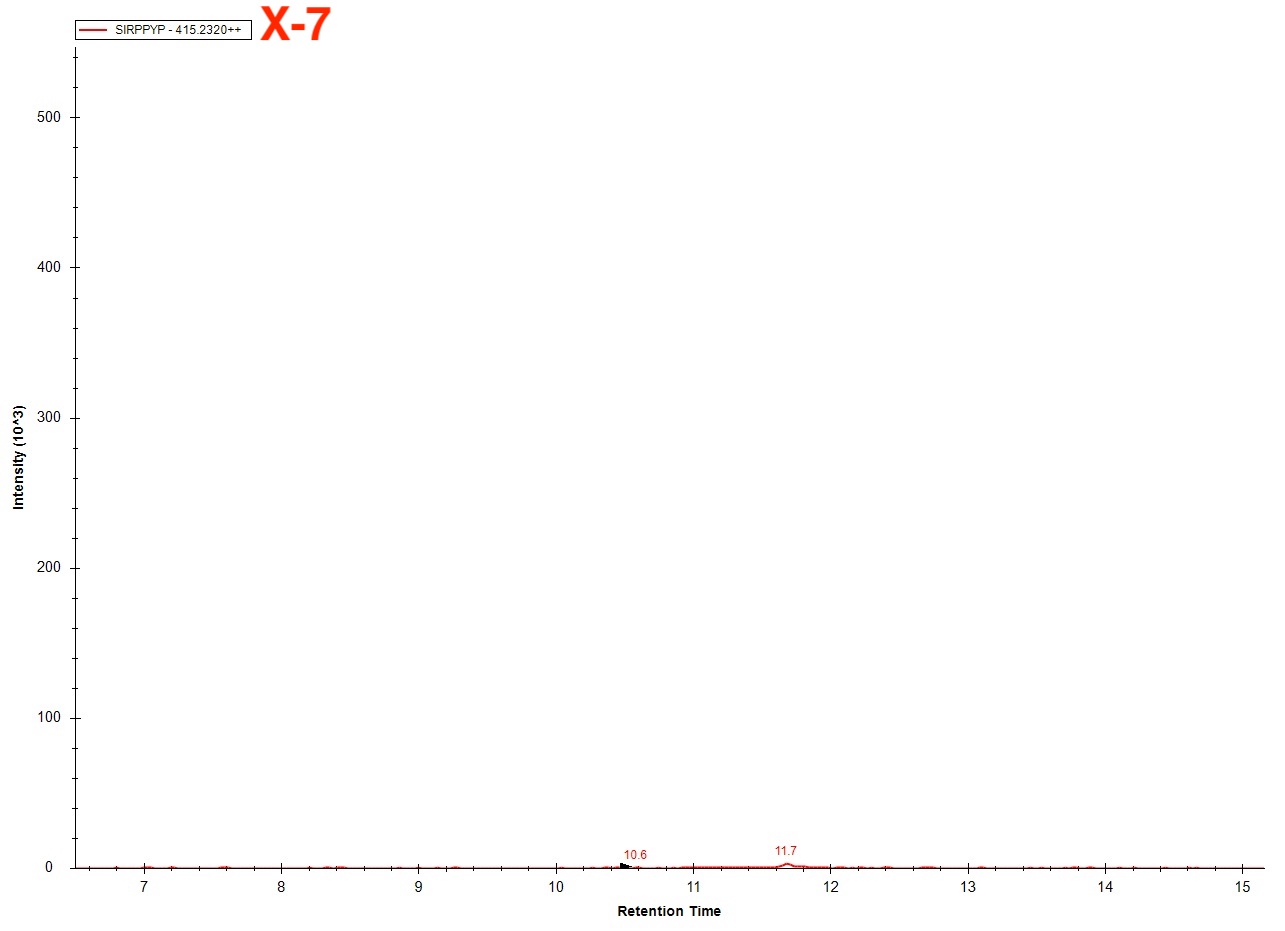
**

**Figure 84. Ion chromatogram of amino acid sequence SIRPPYP - 415.2320++ (AMELX), individual 9.**

**
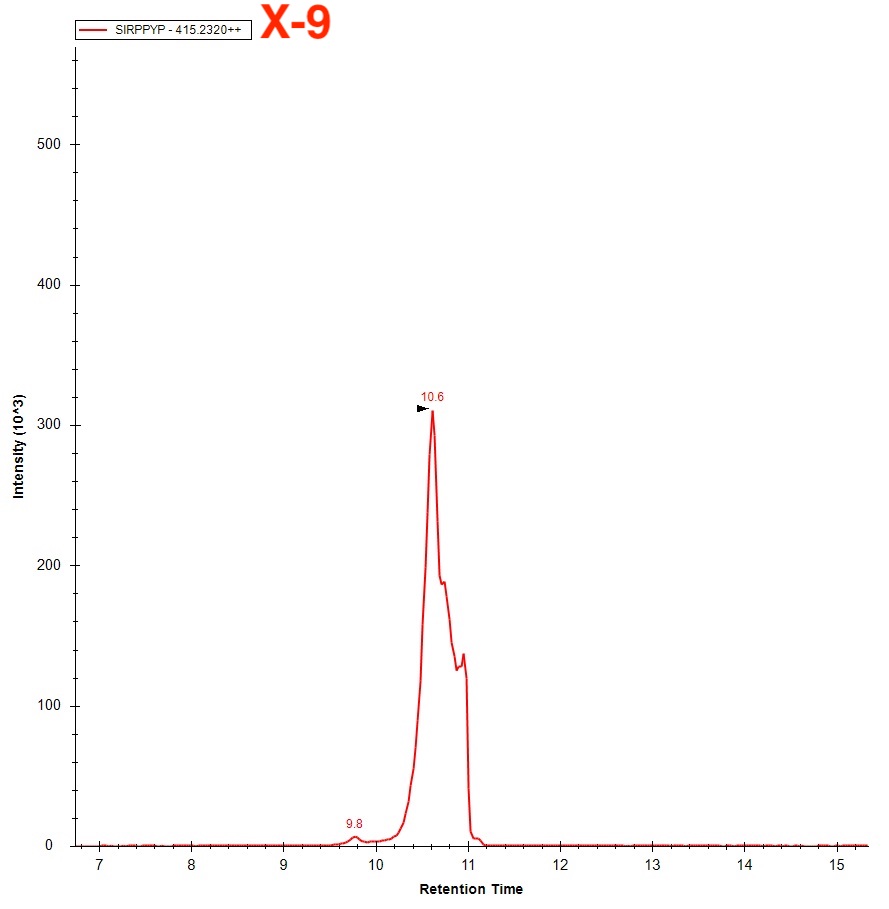
**

**Figure 85. Ion chromatogram of amino acid sequence SIRPPYP - 415.2320++ (AMELX), individual 13.**

**
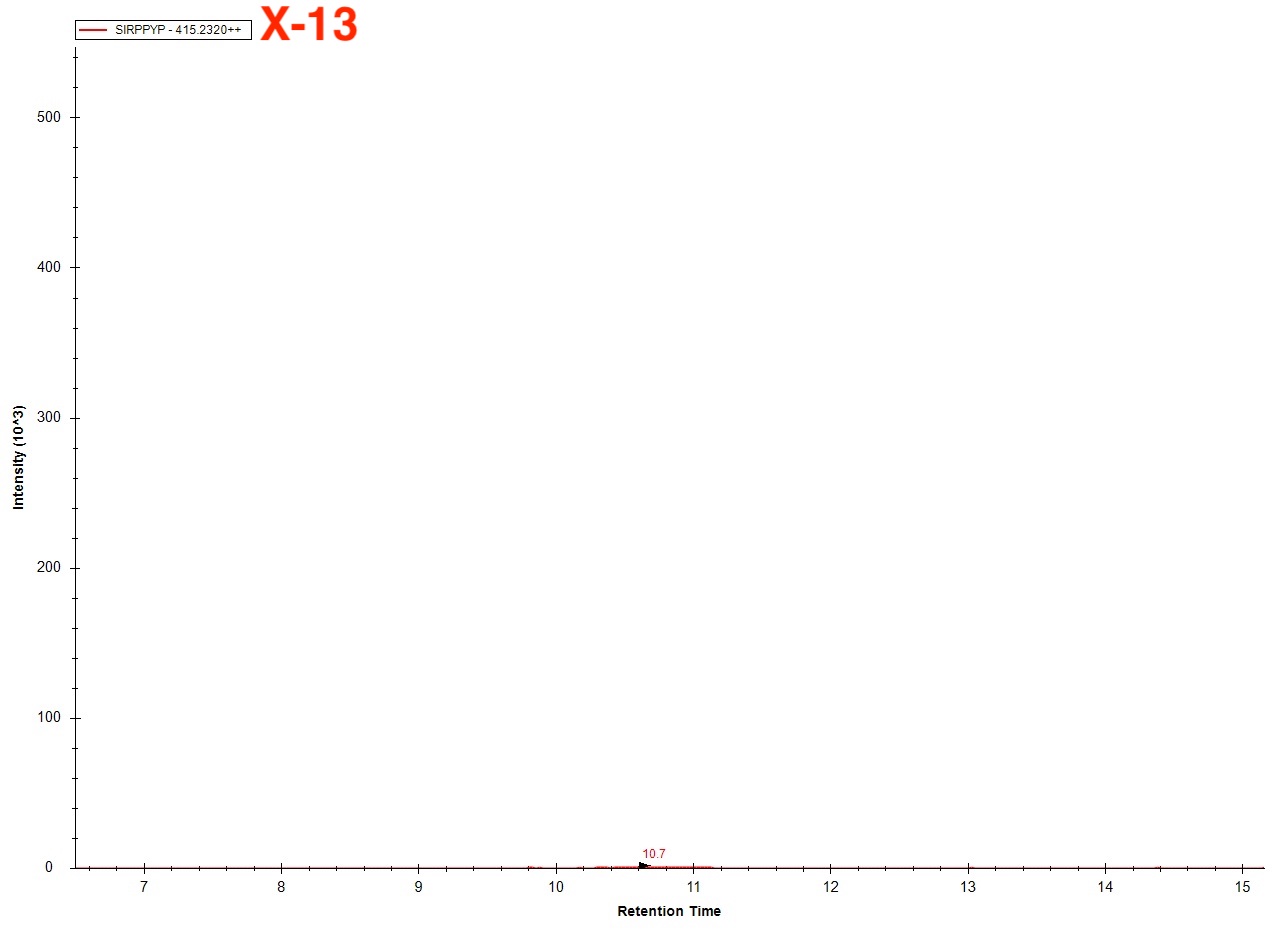
**

**Figure 86. Ion chromatogram of amino acid sequence SIRPPYP - 415.2320++ (AMELX), individual 14.**

**
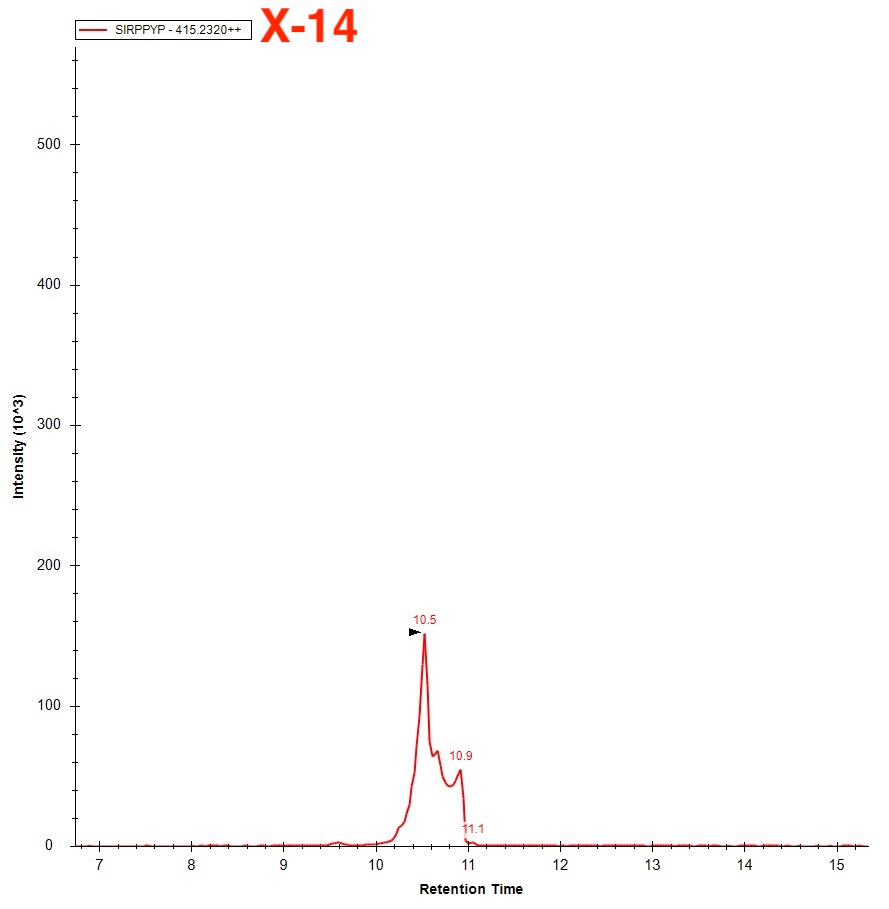
**

**Figure 87. Ion chromatogram of amino acid sequence SIRPPYP - 415.2320++ (AMELX), individual 15.**

**
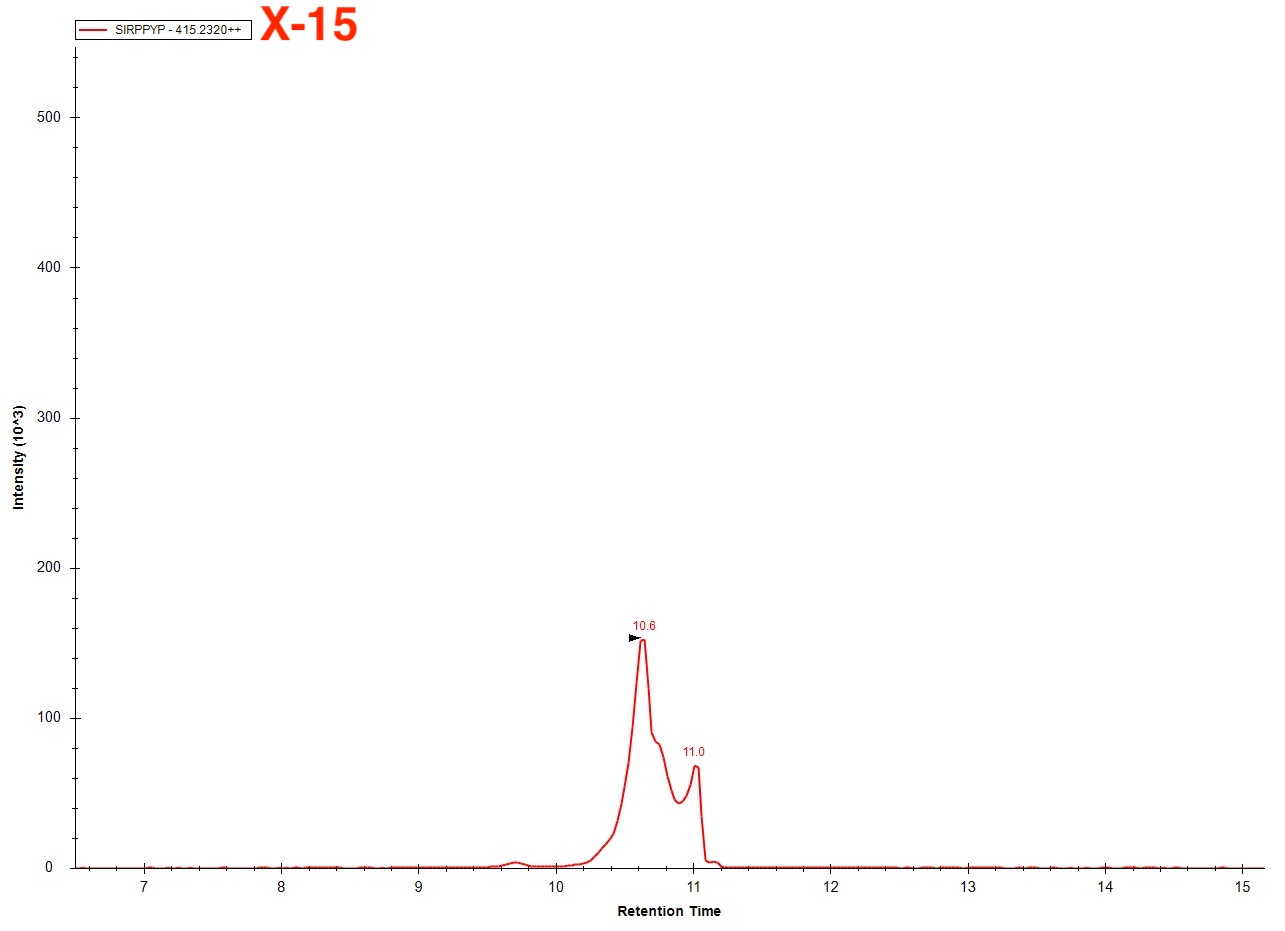
**

**Figure 88. Ion chromatogram of amino acid sequence SIRPPYP - 415.2320++ (AMELX), individual 16.**

**
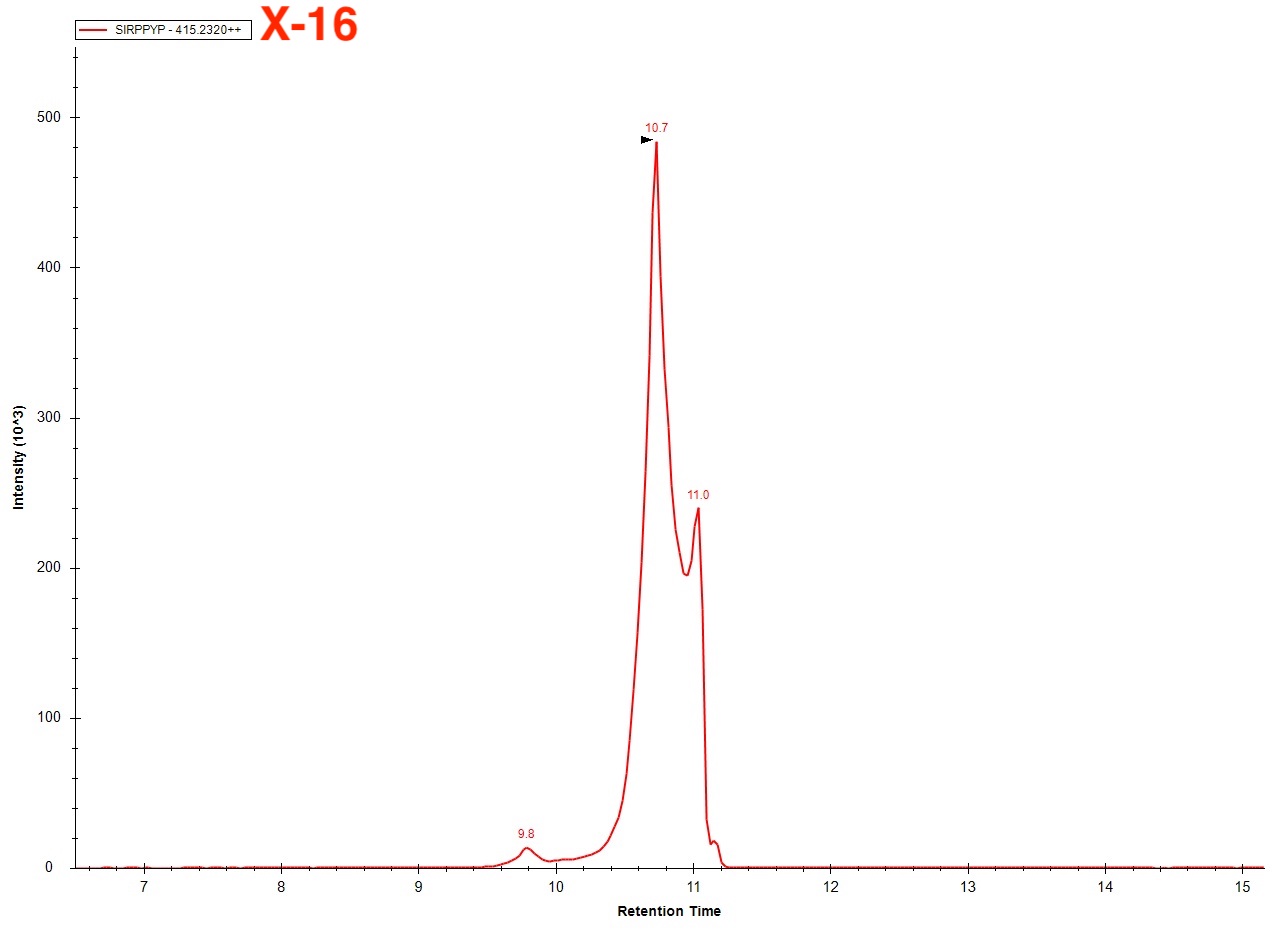
**

**Figure 89. Ion chromatogram of amino acid sequence SIRPPYP - 415.2320++ (AMELX), individual 17.**

**
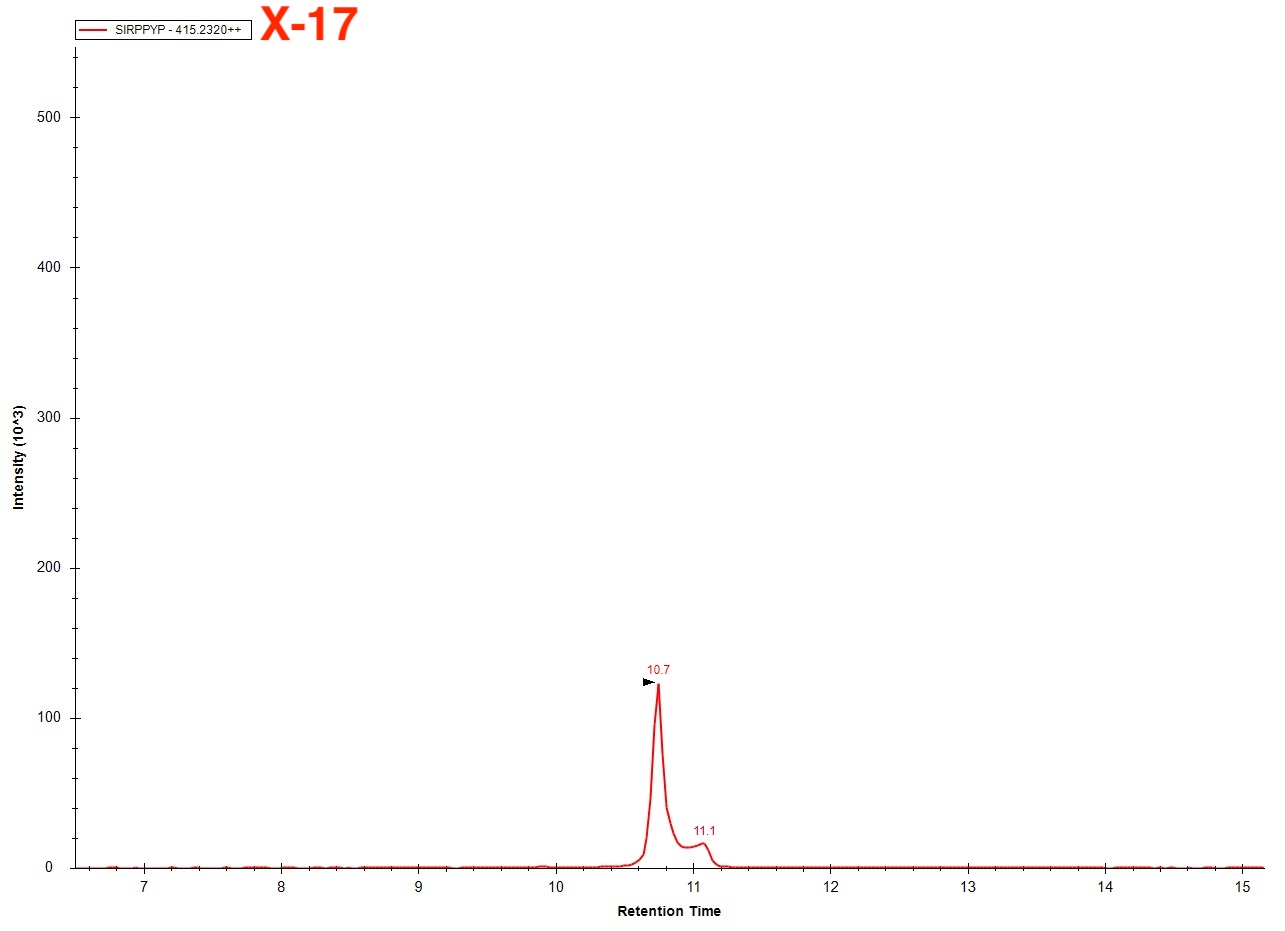
**

**Figure 90. Ion chromatogram of amino acid sequence SIRPPYP - 415.2320++ (AMELX), individual 20.**

**
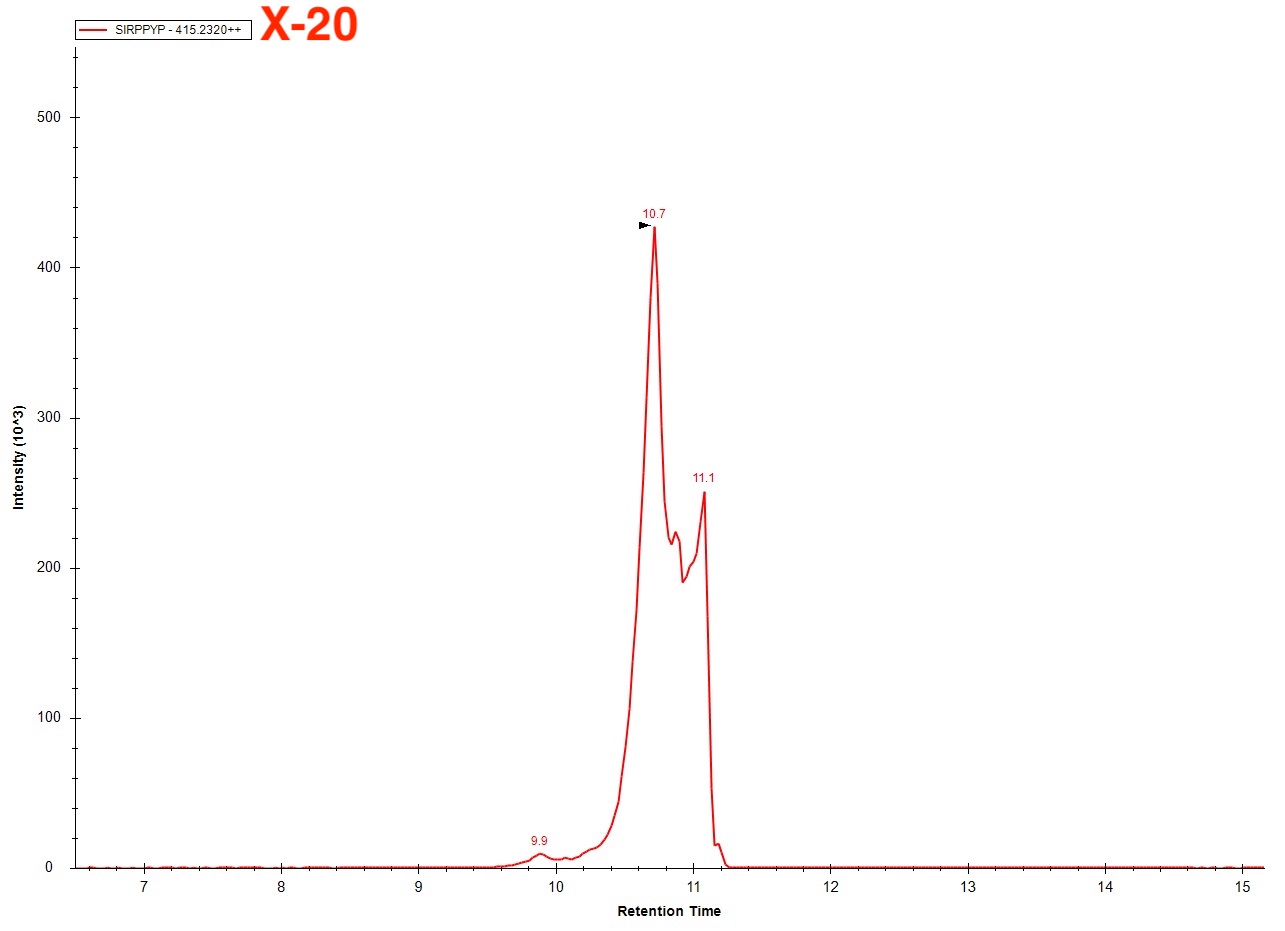
**

**Figure 91. Ion chromatogram of amino acid sequence SIRPPYP - 415.2320++ (AMELX), individual 21.**

**
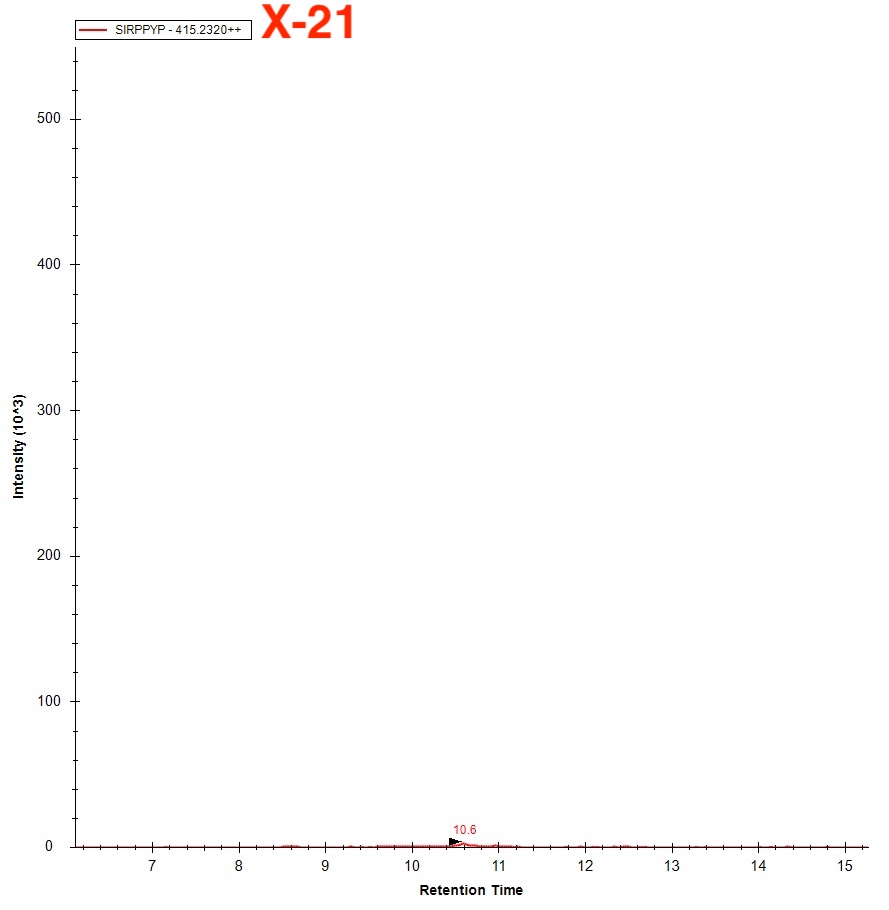
**

**Figure 92. Ion chromatogram of amino acid sequence SIRPPYP - 415.2320++ (AMELX), individual 22.**

**
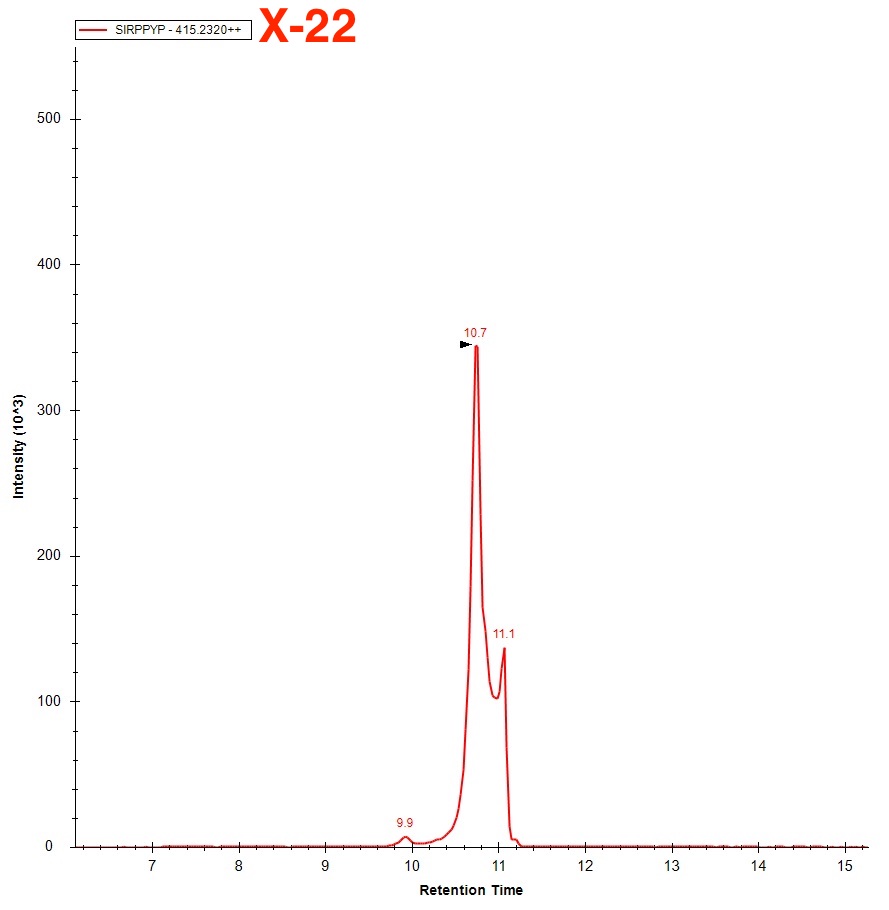
**

**Figure 93. Ion chromatogram of amino acid sequence SIRPPYP - 415.2320++ (AMELX), individual 26.**

**
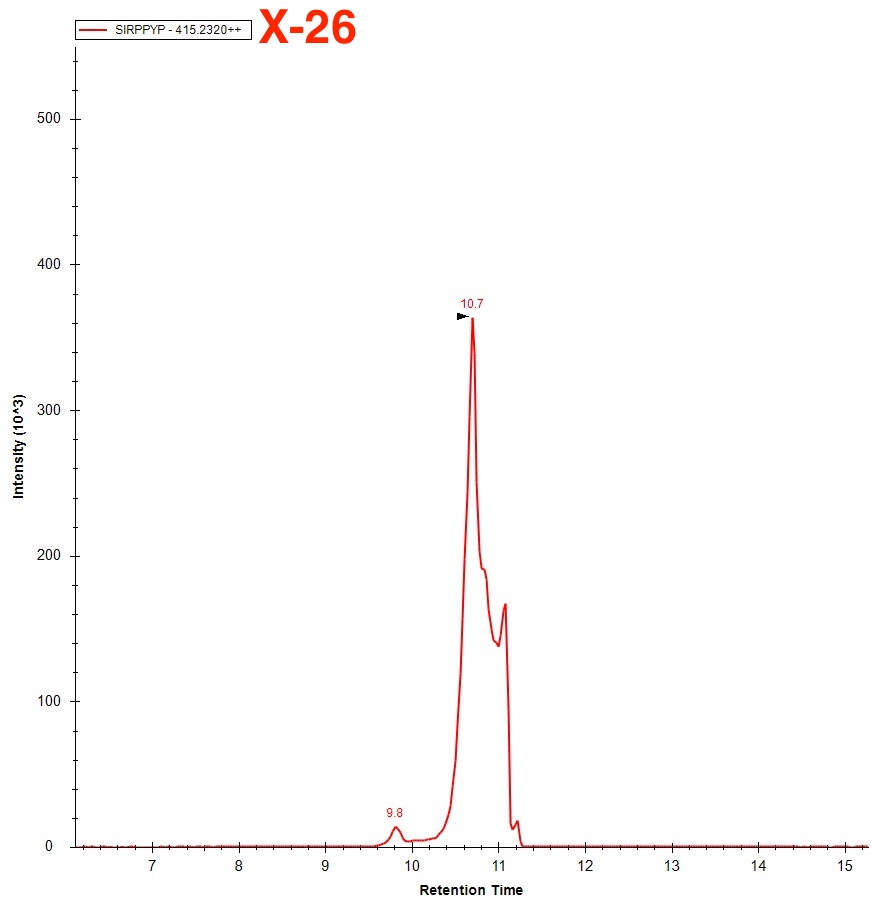
**

**Figure 94. Ion chromatogram of amino acid sequence SIRPPYP - 415.2320++ (AMELX), individual 36.**

**
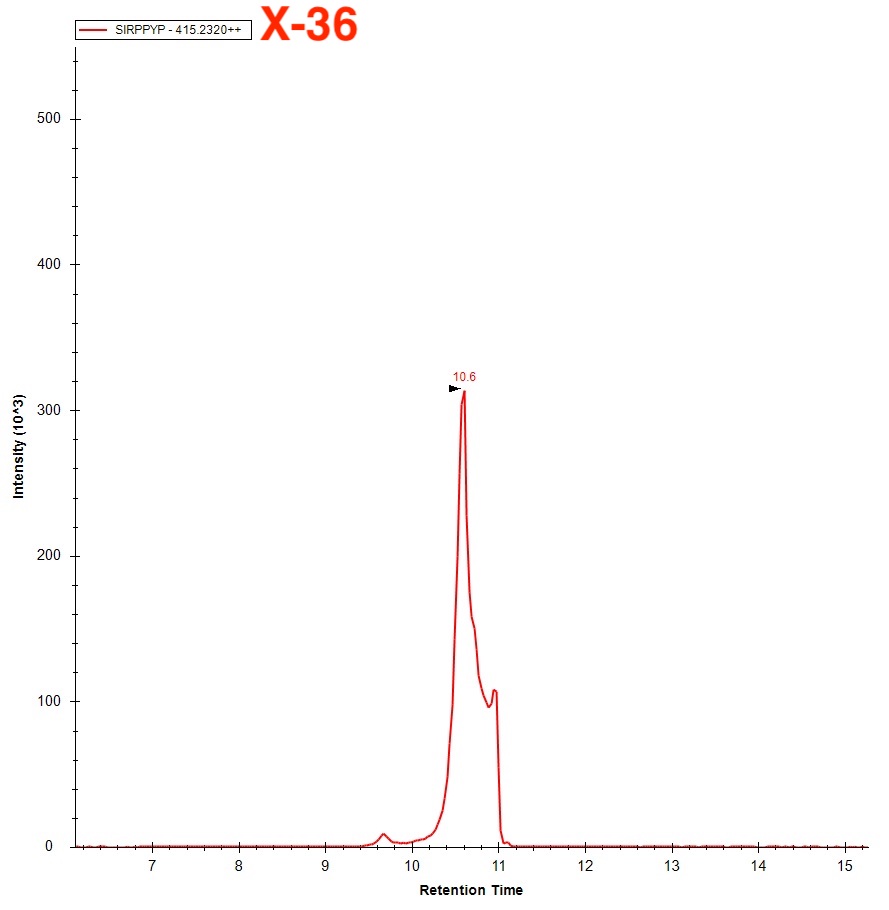
**

**Figure 95. Ion chromatogram of amino acid sequence SIRPPYP - 415.2320++ (AMELX), individual 38.**

**
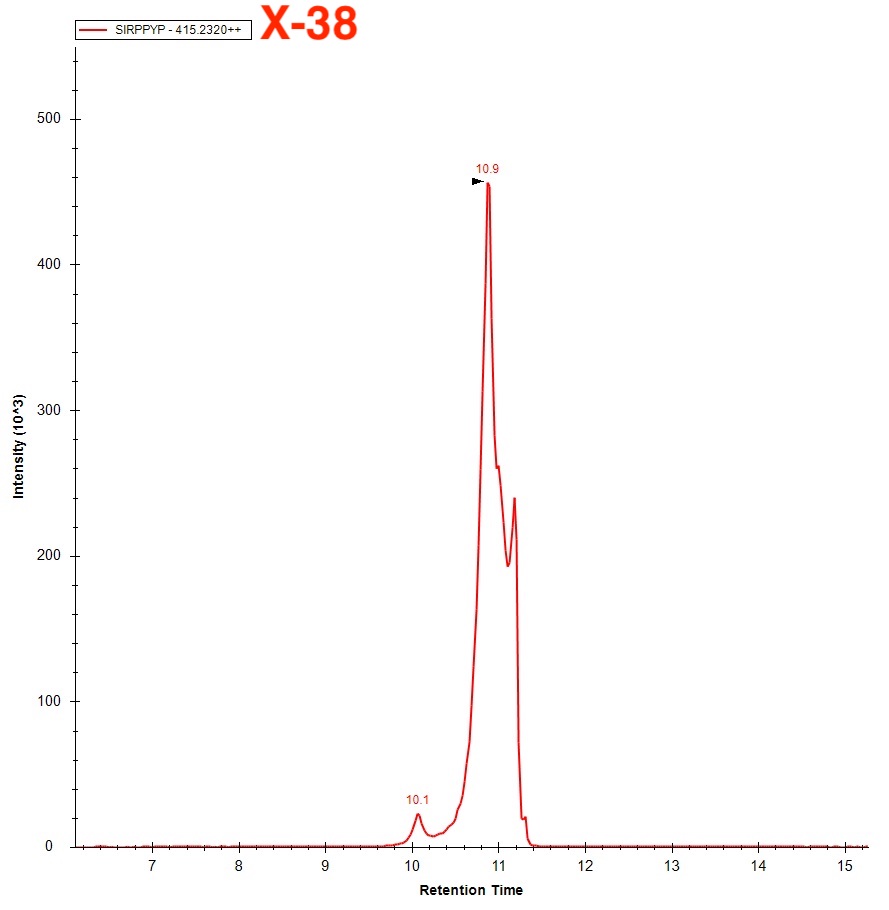
**

**Figure 96. Ion chromatogram of amino acid sequence SIRPPYP - 415.2320++ (AMELX), individual 39.**

**
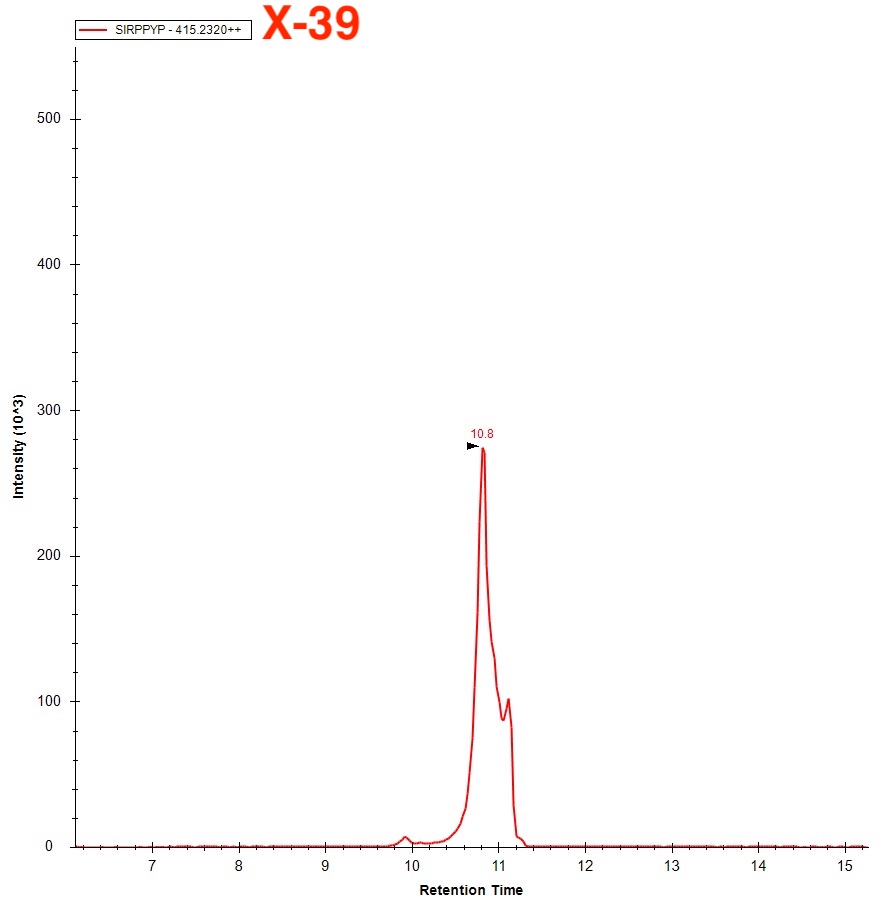
**

**Figure 97. Ion chromatogram of amino acid sequence SIRPPYP - 415.2320++ (AMELX), individual 40.**

**
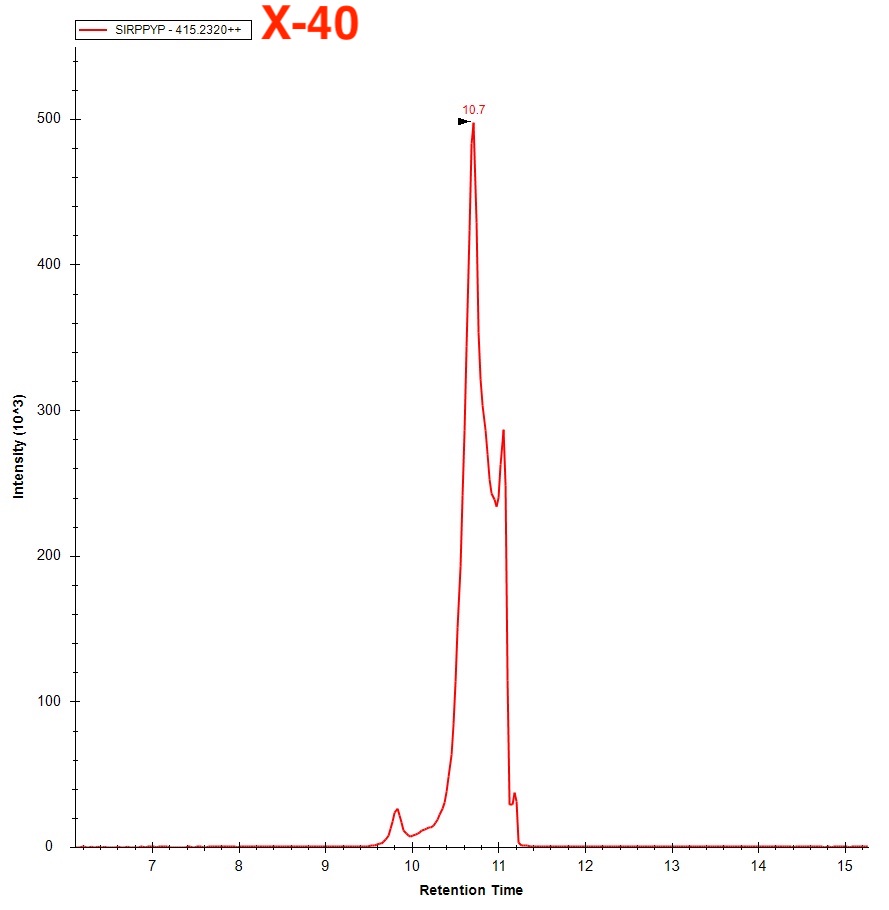
**

**Figure 98. Ion chromatogram of amino acid sequence SIRPPYP - 415.2320++ (AMELX), individual 41.**

**
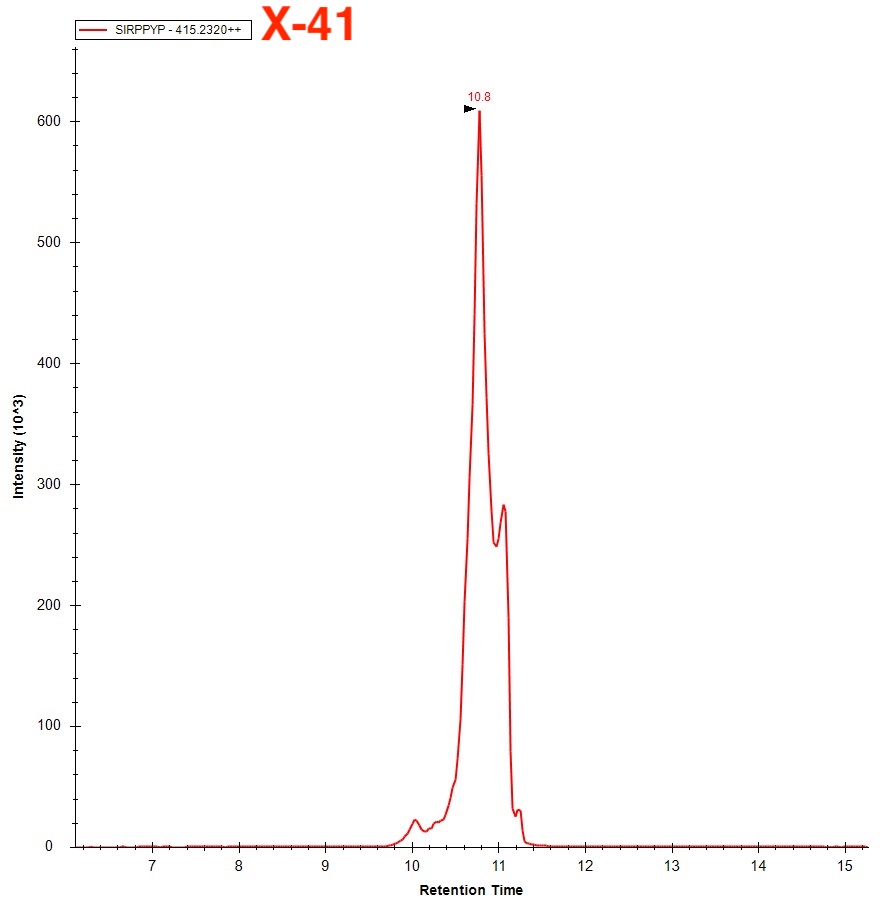
**

**Figure 99. Ion chromatogram of amino acid sequence SIRPPYP - 415.2320++ (AMELX), individual 42.**

**
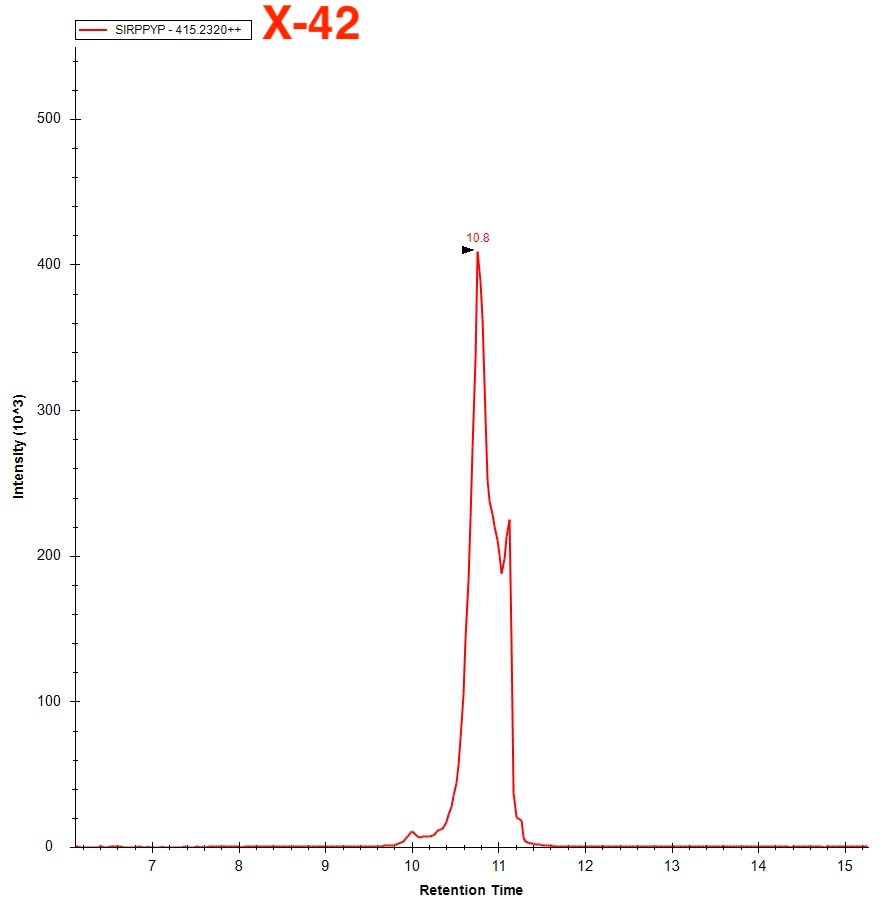
**

**Figure 100. Ion chromatogram of amino acid sequence SIRPPYP - 415.2320++ (AMELX), individual 44.**

**
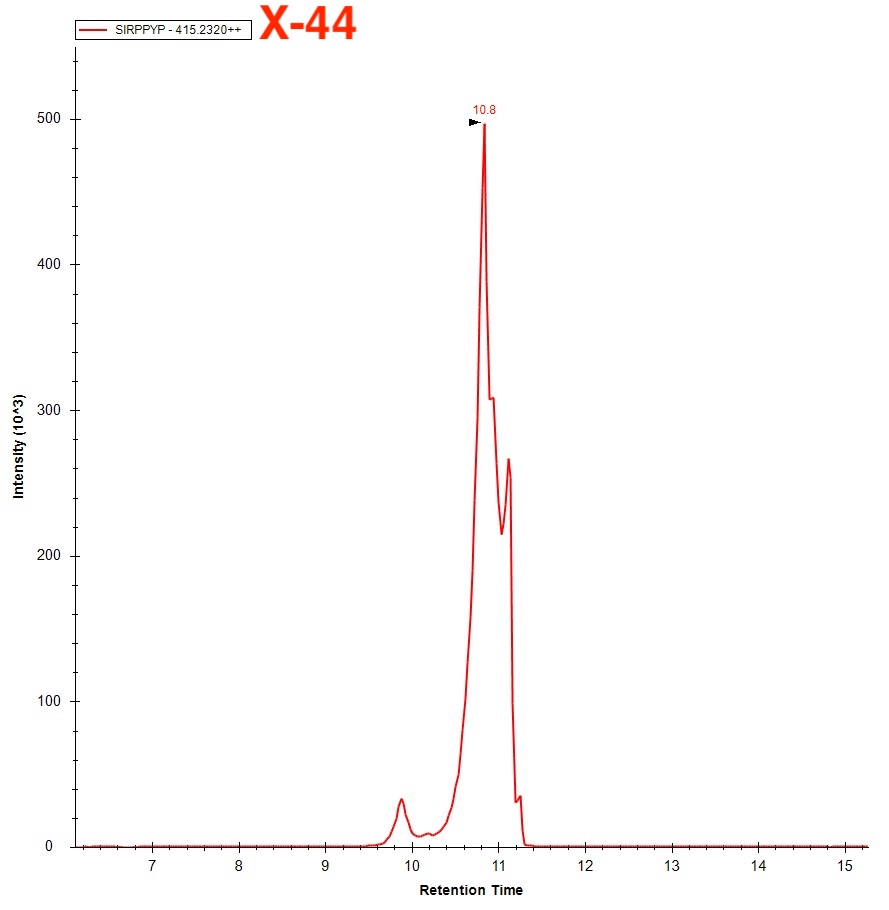
**

**Figure 101. Ion chromatogram of amino acid sequence SIRPPYP - 415.2320++ (AMELX), individual 46.**

**Figure 102. Ion chromatogram of amino acid sequence SIRPPYP - 415.2320++ (AMELX), individual 54.5.**

**Figure 103. Ion chromatogram of amino acid sequence SIRPPYP - 415.2320++ (AMELX), individual SLR.**

**Figure 104. Ion chromatogram of amino acid sequence SIRPPYP - 415.2320++ (AMELX), individual SSJ.**

**Figure 105. Ion chromatogram of amino acid sequence YEVLTPLK - 481.7815++ (AMELX), individual 1.**

**Figure 106. Ion chromatogram of amino acid sequence YEVLTPLK - 481.7815++ (AMELX), individual 2.**

**Figure 107. Ion chromatogram of amino acid sequence YEVLTPLK - 481.7815++ (AMELX), individual 4.**

**Figure 108. Ion chromatogram of amino acid sequence YEVLTPLK - 481.7815++ (AMELX), individual 6.**

**Figure 109. Ion chromatogram of amino acid sequence YEVLTPLK - 481.7815++ (AMELX), individual 7.**

**Figure 110. Ion chromatogram of amino acid sequence YEVLTPLK - 481.7815++ (AMELX), individual 9.**

**Figure 111. Ion chromatogram of amino acid sequence YEVLTPLK - 481.7815++ (AMELX), individual 13.**

**Figure 112. Ion chromatogram of amino acid sequence YEVLTPLK - 481.7815++ (AMELX), individual 14.**

**Figure 113. Ion chromatogram of amino acid sequence YEVLTPLK - 481.7815++ (AMELX), individual 15.**

**Figure 114. Ion chromatogram of amino acid sequence YEVLTPLK - 481.7815++ (AMELX), individual 16.**

**Figure 115. Ion chromatogram of amino acid sequence YEVLTPLK - 481.7815++ (AMELX), individual 17.**

**Figure 116. Ion chromatogram of amino acid sequence YEVLTPLK - 481.7815++ (AMELX), individual 20.**

**Figure 117. Ion chromatogram of amino acid sequence YEVLTPLK - 481.7815++ (AMELX), individual 21.**

**Figure 118. Ion chromatogram of amino acid sequence YEVLTPLK - 481.7815++ (AMELX), individual 22.**

**Figure 119. Ion chromatogram of amino acid sequence YEVLTPLK - 481.7815++ (AMELX), individual 26.**

**Figure 120. Ion chromatogram of amino acid sequence YEVLTPLK - 481.7815++ (AMELX), individual 36.**

**Figure 121. Ion chromatogram of amino acid sequence YEVLTPLK - 481.7815++ (AMELX), individual 38.**

**Figure 122. Ion chromatogram of amino acid sequence YEVLTPLK - 481.7815++ (AMELX), individual 39.**

**Figure 123. Ion chromatogram of amino acid sequence YEVLTPLK - 481.7815++ (AMELX), individual 41.**

**Figure 124. Ion chromatogram of amino acid sequence YEVLTPLK - 481.7815++ (AMELX), individual 42.**

**Figure 125. Ion chromatogram of amino acid sequence YEVLTPLK - 481.7815++ (AMELX), individual 44.**

**Figure 126. Ion chromatogram of amino acid sequence YEVLTPLK - 481.7815++ (AMELX), individual 46.**

**Figure 127. Ion chromatogram of amino acid sequence YEVLTPLK - 481.7815++ (AMELX), individual 54.5.**

**Figure 128. Ion chromatogram of amino acid sequence YEVLTPLK - 481.7815++ (AMELX), individual SLR.**

**Figure 129. Ion chromatogram of amino acid sequence YEVLTPLK - 481.7815++ (AMELX), individual SSJ.**

**AMELXY Ion chromatograms**

**Figure 130. Ion chromatogram of amino acid sequence LPPHPGHPGYINF - 723.8619++**

**(AMELXY), individual 1.**

**Figure 131. Ion chromatogram of amino acid sequence LPPHPGHPGYINF - 723.8619++**

**(AMELXY), individual 2.**

**Figure 132. Ion chromatogram of amino acid sequence LPPHPGHPGYINF - 723.8619++**

**(AMELXY), individual 4.**

**Figure 133. Ion chromatogram of amino acid sequence LPPHPGHPGYINF - 723.8619++**

**(AMELXY), individual 6.**

**Figure 134. Ion chromatogram of amino acid sequence LPPHPGHPGYINF - 723.8619++**

**(AMELXY), individual 7.**

**Figure 135. Ion chromatogram of amino acid sequence LPPHPGHPGYINF - 723.8619++**

**(AMELXY), individual 9.**

**Figure 136. Ion chromatogram of amino acid sequence LPPHPGHPGYINF - 723.8619++**

**(AMELXY), individual 13.**

**Figure 137. Ion chromatogram of amino acid sequence LPPHPGHPGYINF - 723.8619++**

**(AMELXY), individual 14.**

**Figure 138. Ion chromatogram of amino acid sequence LPPHPGHPGYINF - 723.8619++**

**(AMELXY), individual 15.**

**Figure 139. Ion chromatogram of amino acid sequence LPPHPGHPGYINF - 723.8619++**

**(AMELXY), individual 16.**

**Figure 140. Ion chromatogram of amino acid sequence LPPHPGHPGYINF - 723.8619++**

**(AMELXY), individual 17.**

**Figure 141. Ion chromatogram of amino acid sequence LPPHPGHPGYINF - 723.8619++**

**(AMELXY), individual 20.**

**Figure 142. Ion chromatogram of amino acid sequence LPPHPGHPGYINF - 723.8619++**

**(AMELXY), individual 21.**

**Figure 143. Ion chromatogram of amino acid sequence LPPHPGHPGYINF - 723.8619++**

**(AMELXY), individual 22.**

**Figure 144. Ion chromatogram of amino acid sequence LPPHPGHPGYINF - 723.8619++**

**(AMELXY), individual 26.**

**Figure 145. Ion chromatogram of amino acid sequence LPPHPGHPGYINF - 723.8619++**

**(AMELXY), individual 36.**

**Figure 146. Ion chromatogram of amino acid sequence LPPHPGHPGYINF - 723.8619++**

**(AMELXY), individual 38.**

**Figure 147. Ion chromatogram of amino acid sequence LPPHPGHPGYINF - 723.8619++**

**(AMELXY), individual 39.**

**Figure 148. Ion chromatogram of amino acid sequence LPPHPGHPGYINF - 723.8619++**

**(AMELXY), individual 40.**

**Figure 149. Ion chromatogram of amino acid sequence LPPHPGHPGYINF - 723.8619++**

**(AMELXY), individual 41.**

**Figure 150. Ion chromatogram of amino acid sequence LPPHPGHPGYINF - 723.8619++**

**(AMELXY), individual 42.**

**Figure 151. Ion chromatogram of amino acid sequence LPPHPGHPGYINF - 723.8619++**

**(AMELXY), individual 44.**

**Figure 152. Ion chromatogram of amino acid sequence LPPHPGHPGYINF - 723.8619++**

**(AMELXY), individual 46.**

**Figure 153. Ion chromatogram of amino acid sequence LPPHPGHPGYINF - 723.8619++**

**(AMELXY), individual 54.5.**

**Figure 154. Ion chromatogram of amino acid sequence LPPHPGHPGYINF - 723.8619++**

**(AMELXY), individual SLR.**

**Figure 155. Ion chromatogram of amino acid sequence LPPHPGHPGYINF - 723.8619++**

**(AMELXY), individual SSJ.**

**Figure 156. Ion chromatogram of amino acid sequence TPLKWYQ - 468.7449++**

**(AMELXY), individual 1.**

**Figure 157. Ion chromatogram of amino acid sequence TPLKWYQ - 468.7449++**

**(AMELXY), individual 2.**

**Figure 158. Ion chromatogram of amino acid sequence TPLKWYQ - 468.7449++**

**(AMELXY), individual 4.**

**Figure 159. Ion chromatogram of amino acid sequence TPLKWYQ - 468.7449++**

**(AMELXY), individual 6.**

**Figure 160. Ion chromatogram of amino acid sequence TPLKWYQ - 468.7449++**

**(AMELXY), individual 7.**

**Figure 161. Ion chromatogram of amino acid sequence TPLKWYQ - 468.7449++**

**(AMELXY), individual 9.**

**Figure 162. Ion chromatogram of amino acid sequence TPLKWYQ - 468.7449++**

**(AMELXY), individual 13.**

**Figure 163. Ion chromatogram of amino acid sequence TPLKWYQ - 468.7449++**

**(AMELXY), individual 14.**

**Figure 164. Ion chromatogram of amino acid sequence TPLKWYQ - 468.7449++**

**(AMELXY), individual 15.**

**Figure 165. Ion chromatogram of amino acid sequence TPLKWYQ - 468.7449++**

**(AMELXY), individual 16.**

**Figure 166. Ion chromatogram of amino acid sequence TPLKWYQ - 468.7449++**

**(AMELXY), individual 17.**

**Figure 167. Ion chromatogram of amino acid sequence TPLKWYQ - 468.7449++**

**(AMELXY), individual 20.**

**Figure 168. Ion chromatogram of amino acid sequence TPLKWYQ - 468.7449++**

**(AMELXY), individual 21.**

**Figure 169. Ion chromatogram of amino acid sequence TPLKWYQ - 468.7449++**

**(AMELXY), individual 22.**

**Figure 170. Ion chromatogram of amino acid sequence TPLKWYQ - 468.7449++**

**(AMELXY), individual 36.**

**Figure 171. Ion chromatogram of amino acid sequence TPLKWYQ - 468.7449++**

**(AMELXY), individual 38.**

**Figure 172. Ion chromatogram of amino acid sequence TPLKWYQ - 468.7449++**

**(AMELXY), individual 39.**

**Figure 173. Ion chromatogram of amino acid sequence TPLKWYQ - 468.7449++**

**(AMELXY), individual 40.**

**Figure 174. Ion chromatogram of amino acid sequence TPLKWYQ - 468.7449++**

**(AMELXY), individual 41.**

**Figure 175. Ion chromatogram of amino acid sequence TPLKWYQ - 468.7449++**

**(AMELXY), individual 42.**

**Figure 176. Ion chromatogram of amino acid sequence TPLKWYQ - 468.7449++**

**(AMELXY), individual 44.**

**Figure 177. Ion chromatogram of amino acid sequence TPLKWYQ - 468.7449++**

**(AMELXY), individual 46.**

**Figure 178. Ion chromatogram of amino acid sequence TPLKWYQ - 468.7449++**

**(AMELXY), individual 54.5.**

**Figure 179. Ion chromatogram of amino acid sequence TPLKWYQ - 468.7449++**

**(AMELXY), individual SLR.**

**Figure 180. Ion chromatogram of amino acid sequence TPLKWYQ - 468.7449++**

**(AMELXY), individual SSJ.**
